# Supplementary material for: The patatin-like protein PlpD forms structurally dynamic homodimers in the Pseudomonas aeruginosa outer membrane
Source: Nat Commun. 2024 May 23;15:4389. doi: 10.1038/s41467-024-48756-6 (PMC11116518; doi:10.1038/s41467-024-48756-6)
Supplement: Supplementary file 1 — Supplementary Information [file 41467_2024_48756_MOESM1_ESM.pdf]

SUPPLEMENTARY INFORMATION

The patatin-like protein PlpD forms novel structurally dynamic homodimers in the *Pseudomonas aeruginosa* outer membrane

Sarah E. Hanson, Tyrone Dowdy, Mioara Larion, Matthew Thomas Doyle, and Harris D. Bernstein

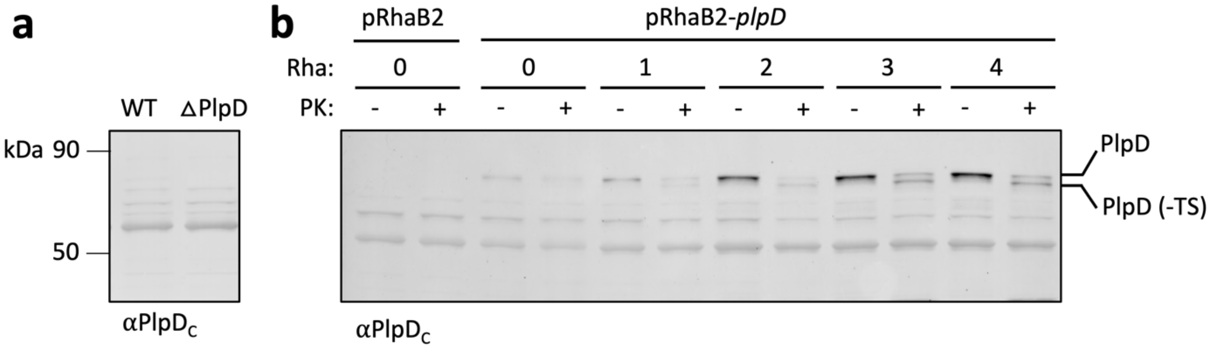

Supplementary Fig. 1: Chromosomally-encoded *plpD* is expressed at a very low level.

a Immunoblot analysis of PlpD levels in PA14 compared to PA14 *ΔplpD*, showing that under standard laboratory conditions native PlpD expression is undetectable. **b** Samples were removed from cultures of PA14 transformed with pRhaB2 or PA14 transformed with pSEH81 after incubation with 0.2% rhamnose for 0-4 hours.

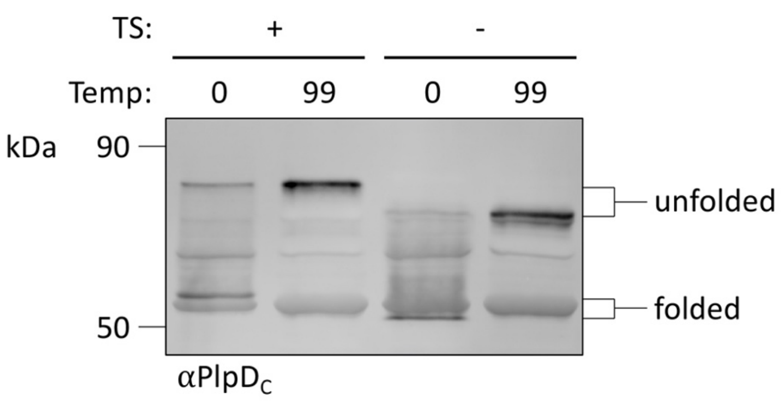

Supplementary Fig. 2: An N-terminal TS tag does not affect the folding of PlpD in *P. aeruginosa*. SEH88 transformed with pSEH81 (which encodes PlpD with an N-terminal TS tag) or pSEH306 (which encodes untagged PlpD) were grown to mid-log phase and the expression of *plpD* was induced by the addition of 0.2% L-rhamnose. Samples were lysed with BugBuster Master Mix and either boiled at 99° C for 20 min or kept on ice (0° C) before proteins were resolved by SDS-PAGE at 4°C. Samples were then analyzed by immunoblot using the anti-$PlpD_C$ antiserum to reveal heat-dependent mobility shifts.

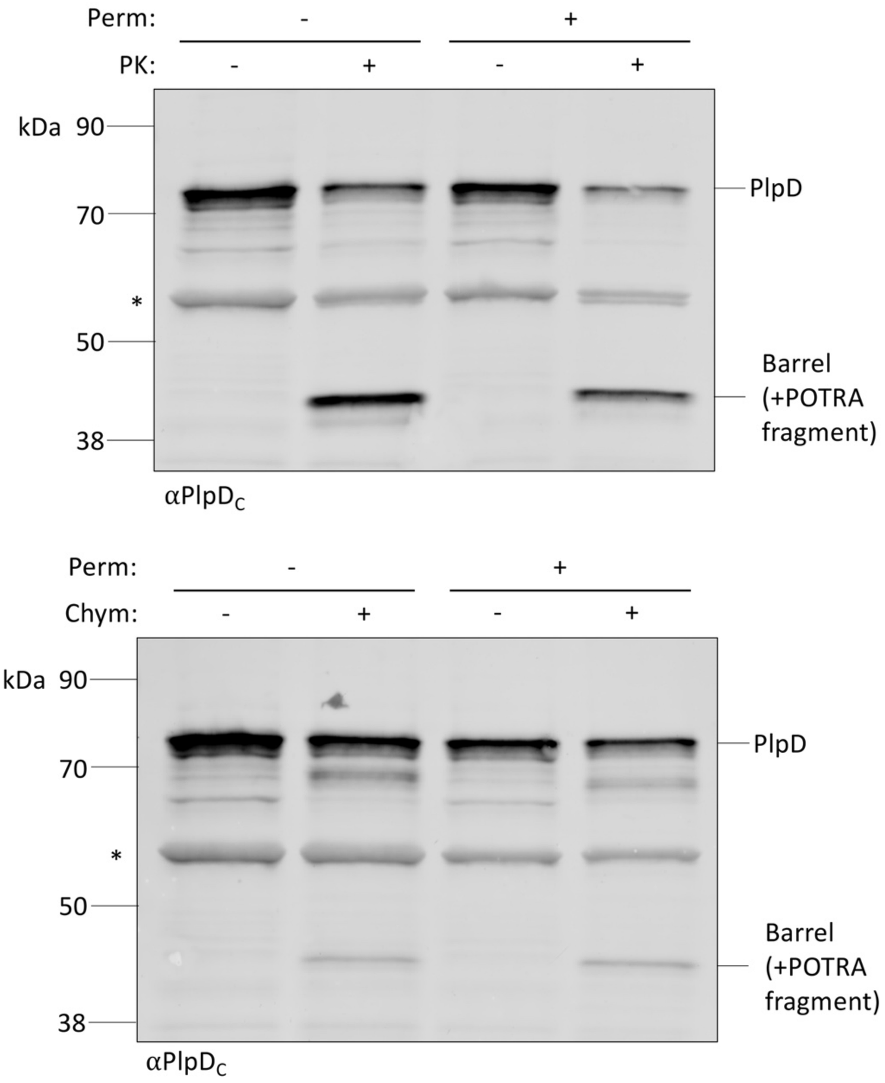

Supplementary Fig. 3: The PL-domain of native (tagless) PlpD is retained in the periplasm. **a** The experiment shown in Fig. 1c was repeated except that SEH88 transformed with pSEH306 was used. The asterisk denotes a non-specific cross-reactive band. **b** SEH88 transformed with pSEH306 were grown to mid-log phase and the expression of *plpD* was induced by the addition of 0.2% L-rhamnose. The culture was divided in half, and the OM of one half was permeabilized (Perm+) while the OM of the other half remained intact (Perm-). Aliquots were then either treated with chymotrypsin (Chym) or left untreated. Samples were analyzed by immunoblot using the anti-PlpD$_C$ antiserum. The asterisk denotes a non-specific cross-reactive band.

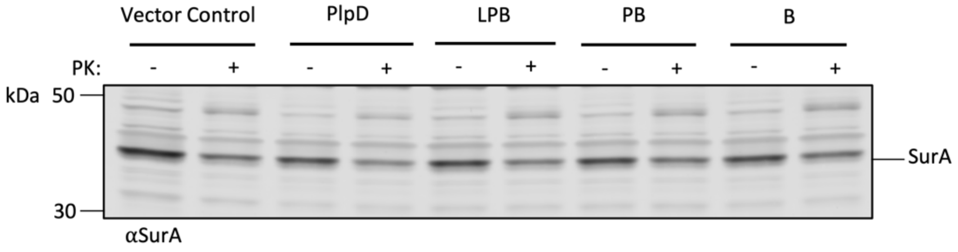

Supplementary Fig. 4: The periplasm of untreated *P. aeruginosa* is accessible to PK. SEH88 transformed with the empty pRhaB2 vector, pSEH81, pSEH93, pSEH95, or pSEH96 were grown and the expression of wild-type *plpD* (WT) or the indicated derivative was induced as in Fig. 1b. Subsequently cells were treated with PK or mock-treated. Immunoblots were then conducted using an anti-SurA antiserum[1].

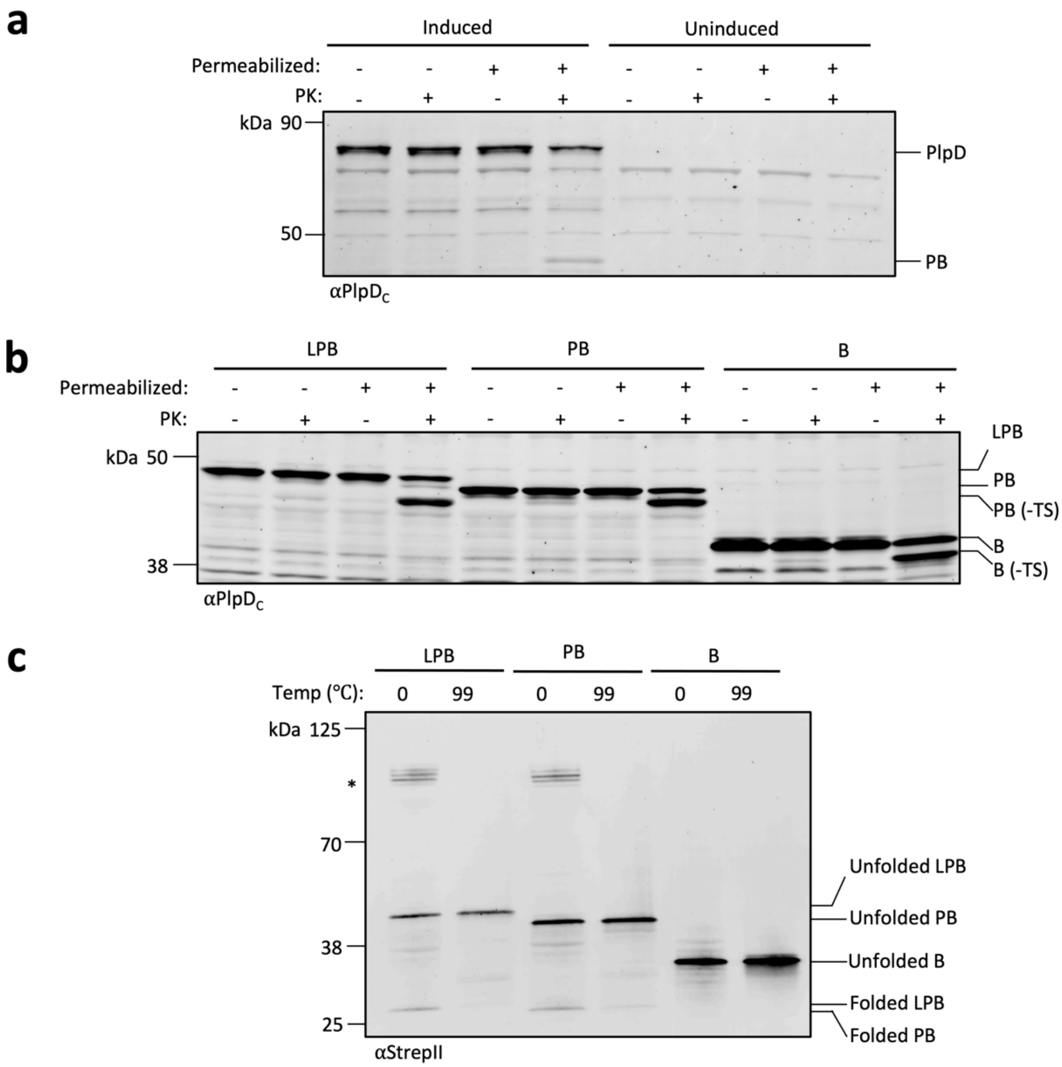

Supplementary Fig. 5: PlpD folds and inserts into the *E. coli* OM and remains resistant to PK digestion. a *E. coli* BL21(DE3) transformed with pSEH71 were grown to late log phase and the culture was divided in half. The expression of *plpD* was then induced in one half by the addition of 0.2% L-rhamnose. The OM of portions of each subculture was permeabilized, and aliquots were then either treated with PK or left untreated. Samples were analyzed by immunoblot using the anti-PlpD$_C$ antiserum. The results show that PlpD was not cleaved in intact cells and thereby indicate that the protein is unlikely to be surface-exposed. When the OM was permeabilized, however, the PL-domain and linker were digested and a fragment corresponding to the size of PB was observed. **b** The expression of the indicated *plpD*

derivative was induced in BL21(DE3) transformed with pMTD1523, pSEH58, or pSEH63 and cells were treated as in (a). Samples were analyzed by immunoblot using the anti-PlpD$_C$ antiserum. As in (a), PK cleaved the PlpD derivatives only when the OM was permeabilized. Protease treatment removed the linker and StrepII tag while leaving the POTRA and β-barrel domains intact. **c** The expression of the indicated *plpD* derivative was induced in BL21(DE3) transformed with pMTD1523, pSEH58, or pSEH63. Samples were lysed with BugBuster Master Mix and either boiled at 99° C for 20 min or kept on ice (0° C) before proteins were resolved by SDS-PAGE at 4°C. Samples were then analyzed by immunoblot with the anti-StrepII antibody. In unheated samples that contained LPB or PB, both a fast-migrating band that corresponds to a folded monomer and high molecular weight bands that correspond to a dimeric form (*) were detected.

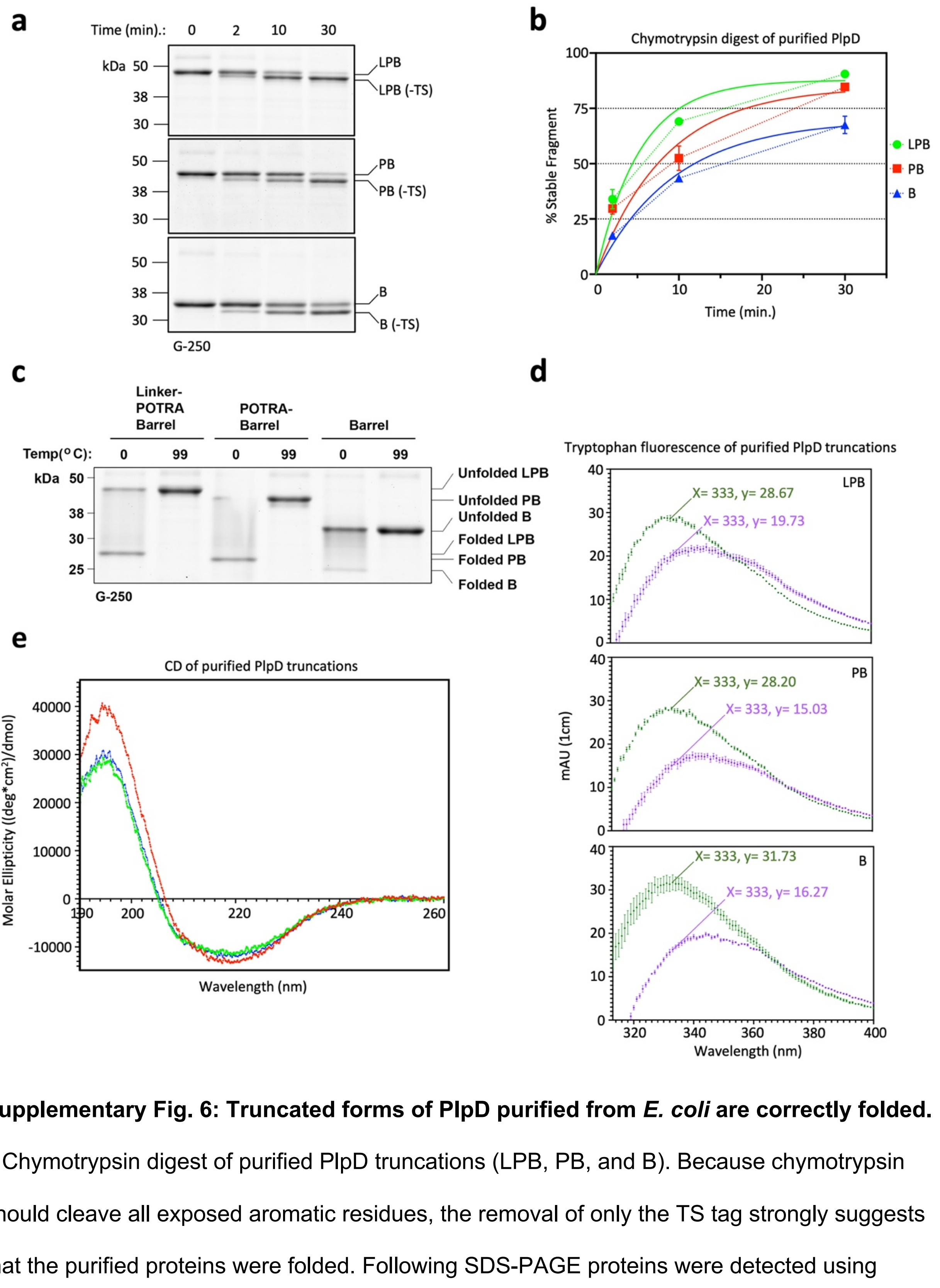

Supplementary Fig. 6: Truncated forms of PlpD purified from *E. coli* are correctly folded. **a** Chymotrypsin digest of purified PlpD truncations (LPB, PB, and B). Because chymotrypsin should cleave all exposed aromatic residues, the removal of only the TS tag strongly suggests that the purified proteins were folded. Following SDS-PAGE proteins were detected using Coomassie Brilliant Blue G-250 stain. **b** Quantitation of chymotrypsin digestion represented by

the “% stable fragment”, i.e., the fraction of the polypeptide resistant to internal cleavage by the protease. Curves of best fit are shown and detailed in Table S11. The error bars represent the standard deviation from the mean. **c** Heat mobility shift assay using the purified PlpD truncations (LPB, PB, and B). Following SDS-PAGE proteins were detected using Coomassie Brilliant Blue G-250 stain. **d** Tryptophan fluorescence analysis of purified PlpD truncations comparing untreated protein (green) with protein treated overnight with 4M guanidinium HCl (purple), a reagent that induces unfolding as indicated by the red shift and lower intensity of the fluorescent signal. mAU: milli-absorbance units. **e** Circular dichroism analysis of the purified PlpD truncations showing the characteristic features of folded β-sheets.

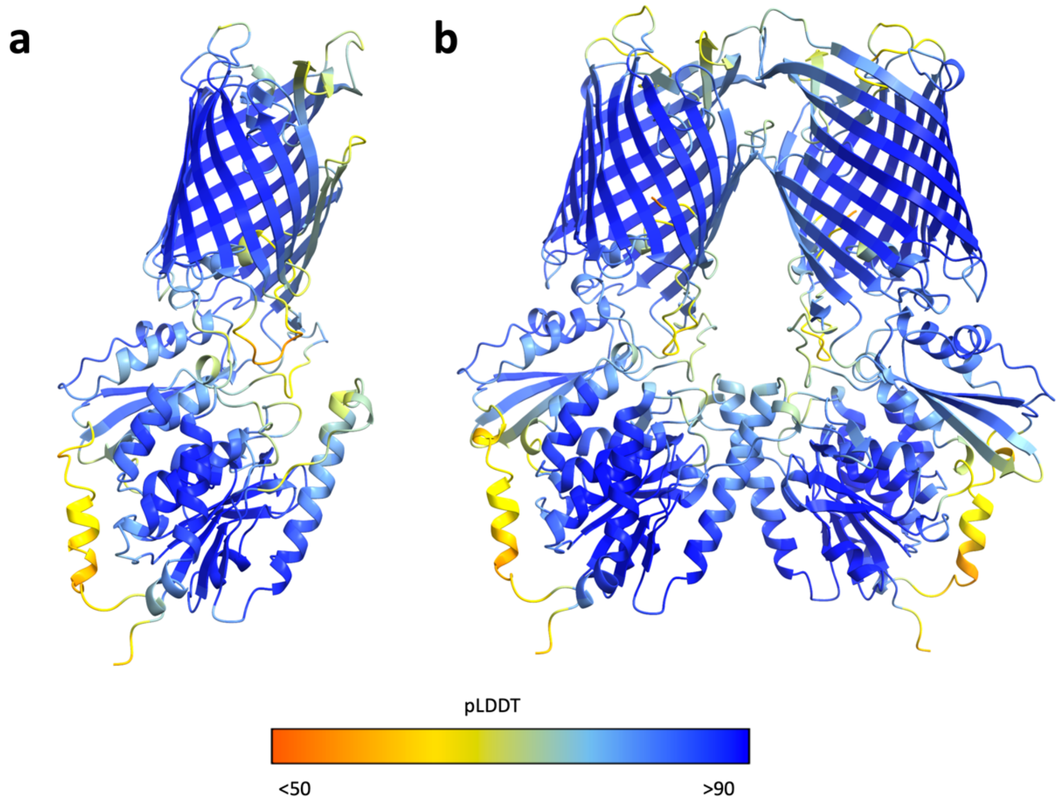

Supplementary Fig. 7: AlphaFold prediction of PlpD monomer and dimer structures with associated confidence scoring. a Predicted model of the *P. aeruginosa* PlpD protein monomer (UniParc: UPI00054992B6) obtained from the AlphaFold database (alphafold.ebi.ac.uk) showing very high confidence for the majority of the structure. The confidence gradient ranges from low (pLDDT score <50; red) to high (pLDDT score >90; dark blue). **b** Top scoring predicted model of PlpD dimer generated by AlphaFold Multimer.

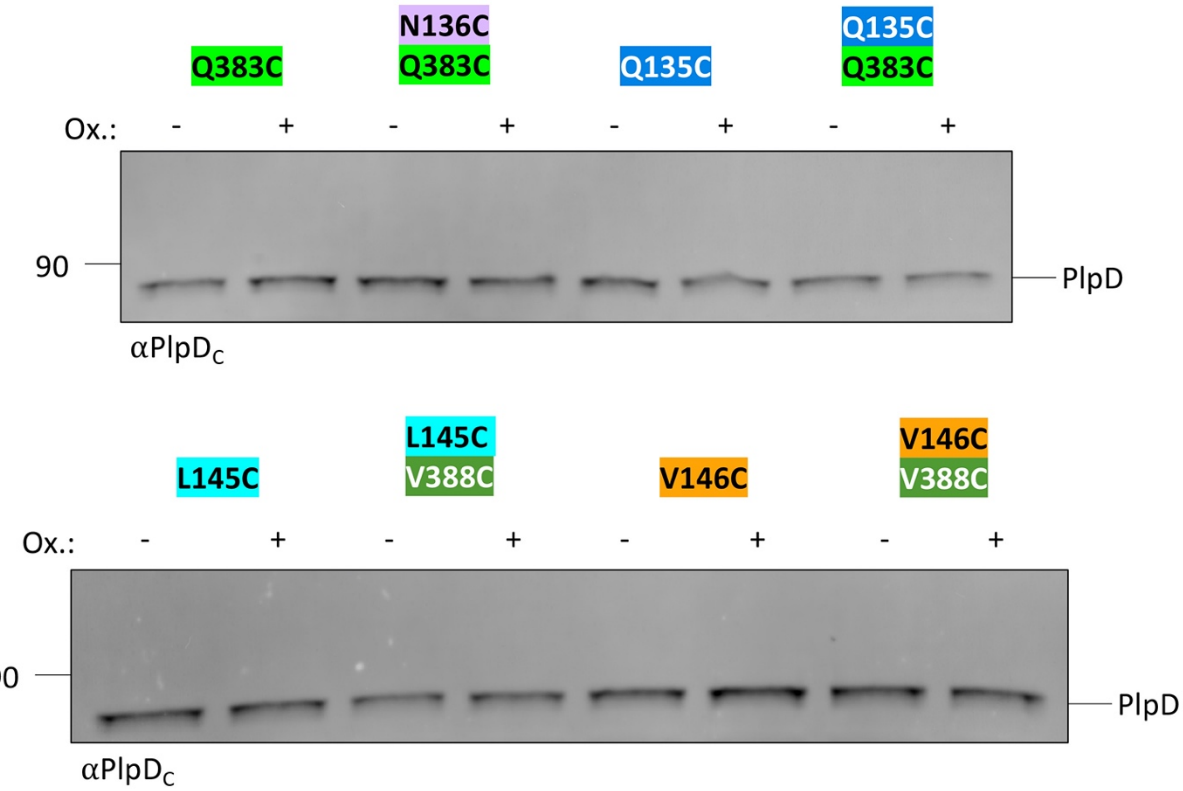

Supplementary Fig. 8: High molecular weight bands result from disulfide bond formation. The reactions shown in Fig. 3b were treated with 150 mM DTT before proteins were resolved by SDS-PAGE. Samples were analyzed by immunoblot using the anti-PlpD$_C$ antiserum.

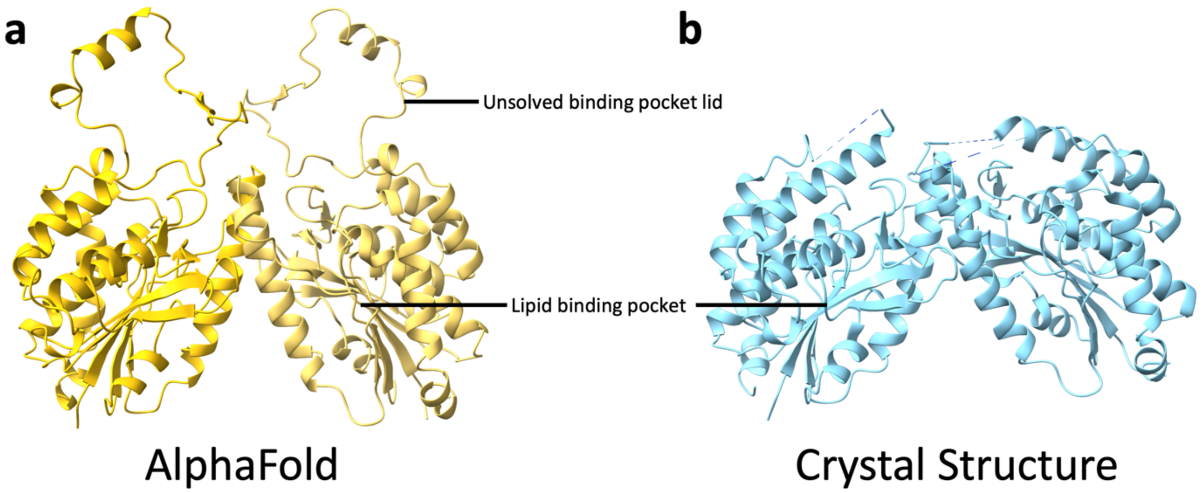

Supplementary Fig. 9: Predicted PL-domain dimer structure is very similar to the crystal structure. a AlphaFold Multimer prediction of PlpD PL-domain dimer (left, yellow). The AlphaFold Multimer model predicts the structure of the lid region of the PL-domain that was unresolved in the crystal structure. **b** The published crystal structure of the dimeric PlpD PL-domain [right, blue; PDB ID: 5FQU (https://www.ncbi.nlm.nih.gov/Structure/pdb/5FQU)].

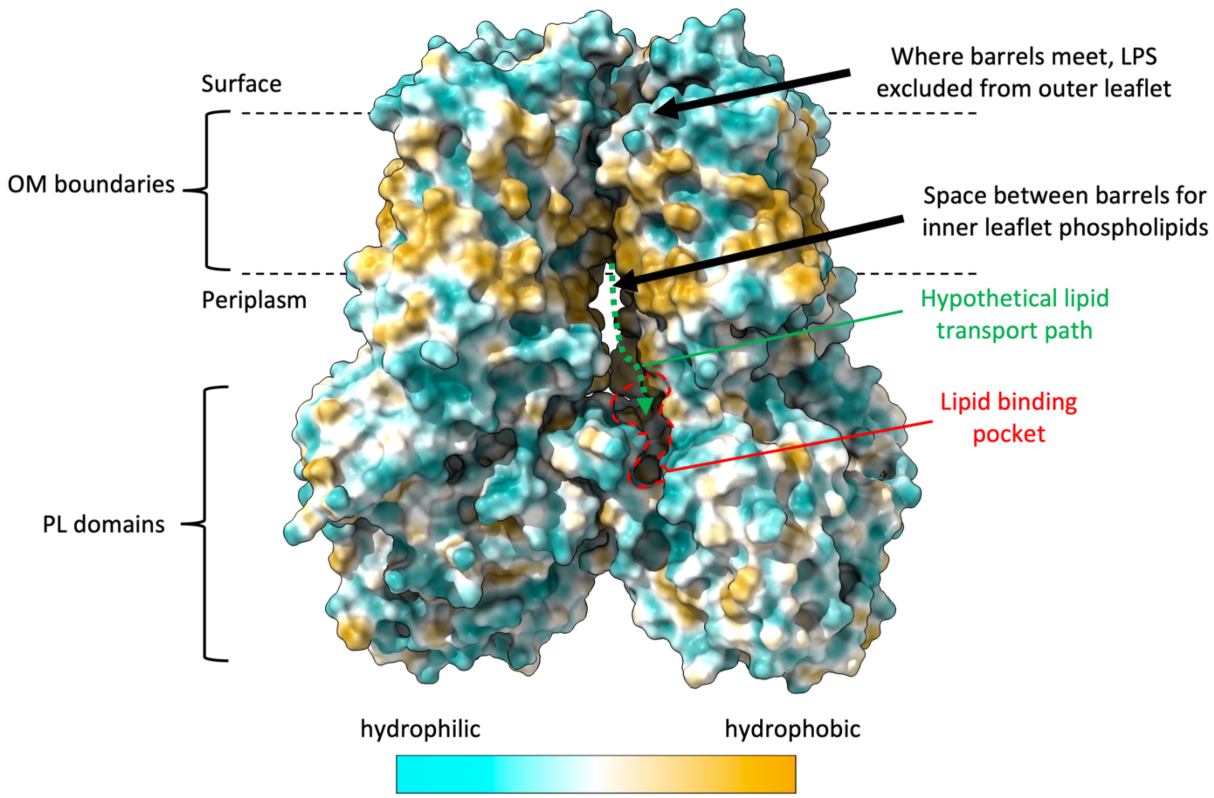

Supplementary Fig. 10: Space-filling model of the PlpD dimer. Space-filling model of the PlpD dimer showing hydrophobic and hydrophilic residues, the exclusion of LPS from the space between the two β-barrels, and the generation of a space between the two PlpD molecules that might accommodate a phospholipid oriented directly above the lipid binding pocket (outlined in red) in the PL-domains. A phospholipid could travel down the hypothetical lipid transport path for hydrolysis (green dashed arrow).

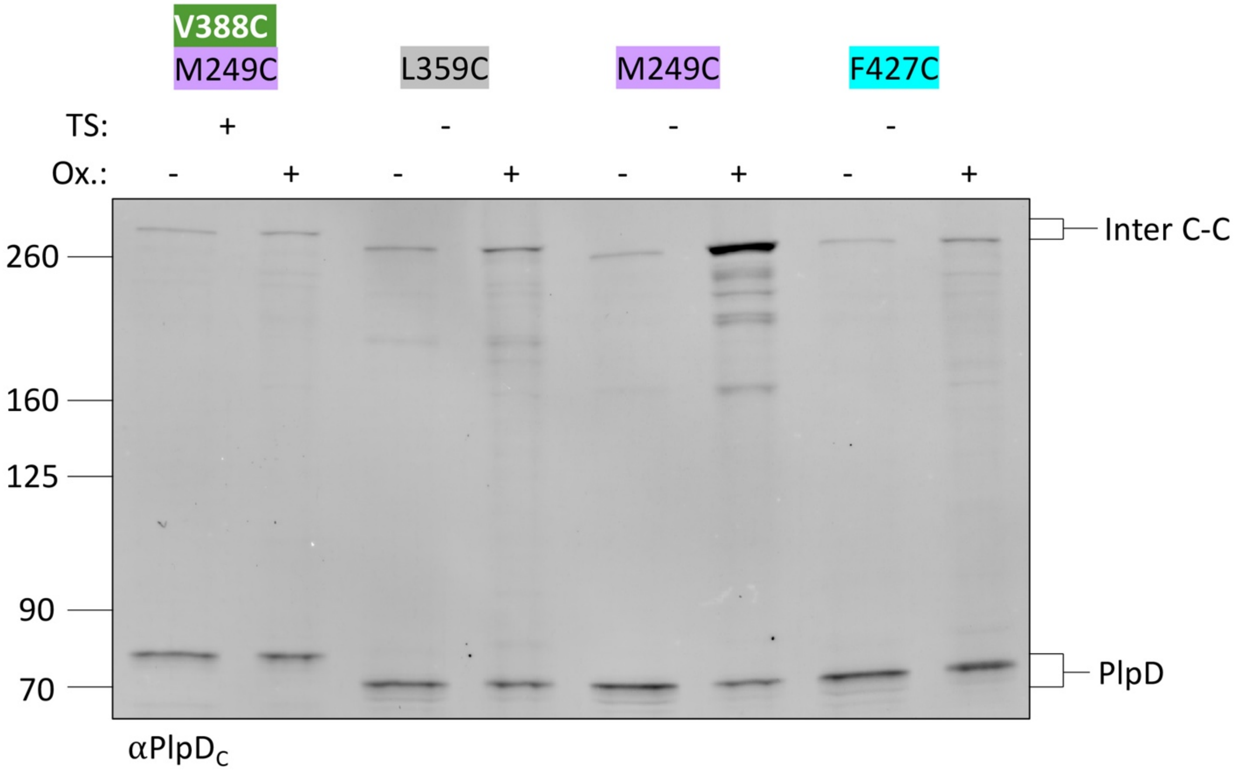

Supplementary Fig. 11: Native (untagged) PlpD forms the same pattern of inter-molecular disulfide bonds as TS-PlpD. The experiment described in Fig. 4c was repeated except that SEH88 transformed with derivatives of pSEH81 and pSEH306 that encoded the indicated cysteine mutant(s) were used.

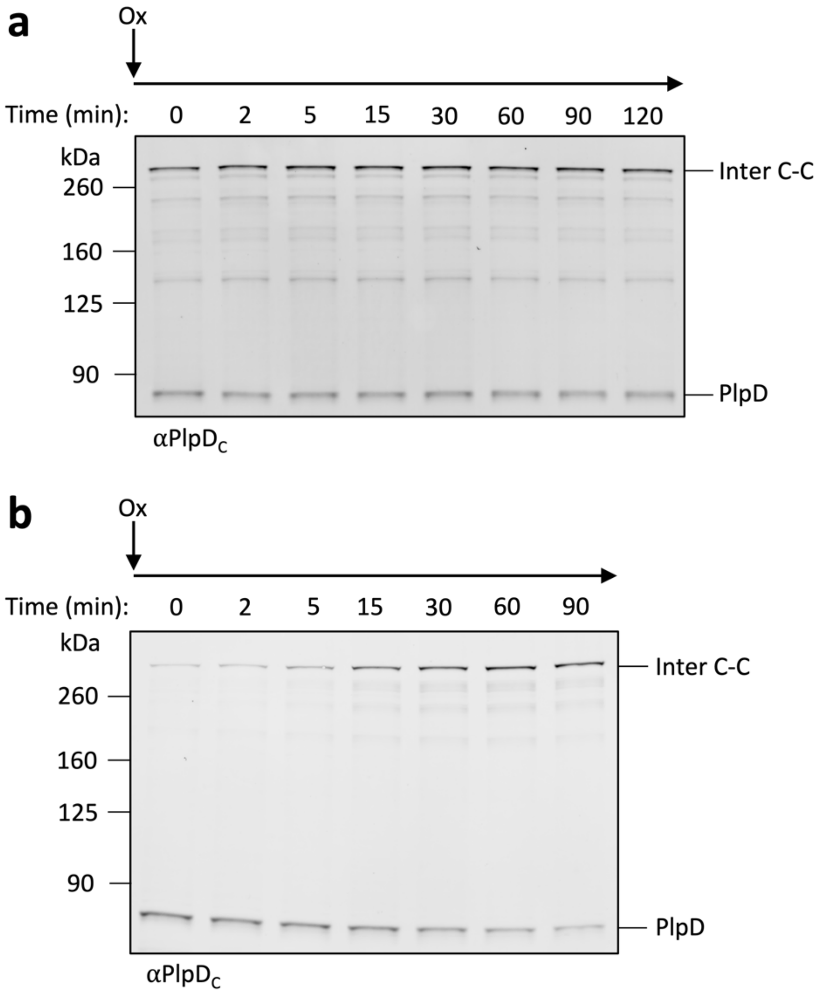

Supplementary Fig. 12: Disulfide bond formation kinetics of the PlpD M249C and I532C mutants. a A representative immunoblot used for the graph in Fig. 4e is shown. **b** A representative immunoblot used for the graph in Fig. 4f is shown.

β-barrel strand 1 interacting region

Species	UniParc ID		
Fusobacterium hwasookii	UPI00027C9D86	FIGTLYSIGYTIDEIEKVLDSLNTESFLESGSDLTNLDLDKKESLKKYS--FYINFDNEL	159
Ralstonia solanacearum	UPI000066CCB11	VVGGLYASGLHADALEQRLSQVNLSDIAFDRKERAKLPQSLREDDFQYPIGLSAGYANG-	202
Burkholderia pseudomallei	UPI00005C3BCB	VVGGLYATGMTAQDMQRRLSQVNLADIAFDVTERSDLPQKKREDERLYIDSLTIGFDSK-	354
Burkholderia cenocepacia	UPI00097BFC68	VVGGLYASGMAADEMQKRLSEVNLADIAFDVTDRADLPQTSREDERLYINGLTLGFGKK-	174
Candidatus Thiodictyon syntrophicum	UPI000C2D36F1	LVGGGYAAGLSPRELEQRVTQVDWNVLFNDDPPRREWPARRKQLSSNPTFDFSIGVRKG-	135
Pseudomonas aeruginosa	UPI00054992B6	VVGGLYASGYTPAELERIALWMDWQQALSDAPPRKDVPFRRKQDDRDFLVKQKISFRDDA	123
Vibrio nigripulchritudo	UPI0003B0D6FA	FVGGMYATGMSASEIESFIHTIDWSSGYRDRVNRDQLPVREKQYYDRYQIQTDLGLRFG-	136
Shewanella oneidensis	UPI00000E1755	YVAGMYALGYSATEVEAIMMGVDWDSGYSDTIPRNVLSYRDKKLRDRYNIPLNIGYSEG-	122
Aeromonas veronii	UPI0007187C63	YVAGMYAMGLSAEEVERTTLAIDWNKGYQDKVGRDELSLRKKQQNEQYQLRTDIGVNGD-	130
		:. *: *. :: :: . :: .	

Supplementary Fig. 13. Alignment of the lid region of PL-Omp85 family members.

Alignment of amino acid sequences of the lid region of nine PL-Omp85 family members (UniParc ID's provided) from a wide range of species. A motif is highlighted in yellow that closely mimics the last several residues of a canonical β-signal that is found at the C-terminus of OMP β-barrels and that is recognized by BamA β-strand 1 to initiate OMP assembly.

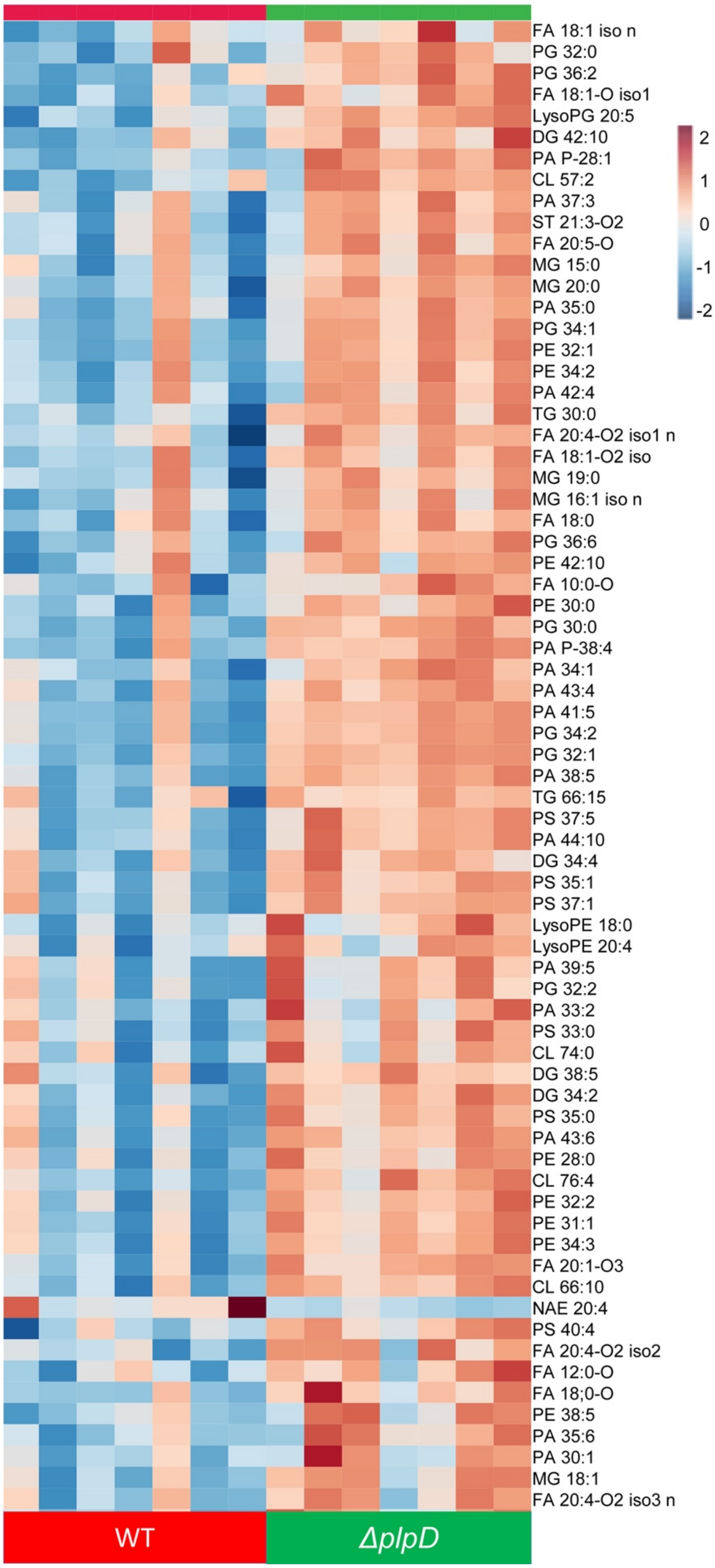
FA 18:1 iso n
PG 32:0
PG 36:2
FA 18:1-O iso1
LysoPG 20:5
DG 42:10
PA P-28:1
CL 57:2
PA 37:3
ST 21:3-O2
FA 20:5-O
MG 15:0
MG 20:0
PA 35:0
PG 34:1
PE 32:1
PE 34:2
PA 42:4
TG 30:0
FA 20:4-O2 iso1 n
FA 18:1-O2 iso
MG 19:0
MG 16:1 iso n
FA 18:0
PG 36:6
PE 42:10
FA 10:0-O
PE 30:0
PG 30:0
PA P-38:4
PA 34:1
PA 43:4
PA 41:5
PG 34:2
PG 32:1
PA 38:5
TG 66:15
PS 37:5
PA 44:10
DG 34:4
PS 35:1
PS 37:1
LysoPE 18:0
LysoPE 20:4
PA 39:5
PG 32:2
PA 33:2
PS 33:0
CL 74:0
DG 38:5
DG 34:2
PS 35:0
PA 43:6
PE 28:0
CL 76:4
PE 32:2
PE 31:1
PE 34:3
FA 20:1-O3
CL 66:10
NAE 20:4
PS 40:4
FA 20:4-O2 iso2
FA 12:0-O
FA 18;0-O
PE 38:5
PA 35:6
PA 30:1
MG 18:1
FA 20:4-O2 iso3 n
2
1
0
-1
-2
WT
ΔplpD

Supplementary Fig. 14: Heatmap of the top 70 lipids differentially produced in wild-type and *ΔplpD* strains. The results were obtained from the experiment shown in Fig. 6. Abbreviations: cardiolipin (CL); diacylglycerol (DG); fatty acid (FA); oxidized fatty acid (FA-O); monoacylated glycerophospholipid (Lyso); monoacylglycerol (MG); N-acylethanolamine (NAE); phosphatidic acid (PA); phosphatidylethanolamine (PE); phosphatidylglycerol (PG); phosphatidylserine (PS); triacylglycerol (TG).

Plasmid	Alias	Notes	Source
pSCrhaB2		rhamnose-inducible expression vector; Tm^R	2
pDONRpEX18Gm	pSEH76	shuttle vector with attp sites and ccdB; Cm^R, Gm^R	3
pMTD607		pSCrhaB2::espP(C) SP-TS-malE-espP948-984-tev-espP985-1300	4
pMTD1523	pMTD607(mtd350/351) + mtd159	pSCrhaB2::plpD312-728StrepII	This work
pSEH58	pMTD1523(seh4/5)	pSCrhaB2::plpD332-728StrepII	This work
pSEH63	pMTD1523(seh4/7)	pSCrhaB2::plpD406-728StrepII	This work
pSEH67	pMTD1523(mtd372)	pSCrhaB2::plpD22-728StrepII	This work
pSEH71	pSEH67(seh13/14)	pSCrhaB2::plpD20-728 No StrepII	
pSEH78	pSEH76(seh23)	pDONRpEX18Gm::plpD upstream/downstream segments (gBlock to delete plpD gene)	This work
pSEH81	pSEH77(seh15/16)	pSCrhaB2::plpD with StrepII tag (gBlock)	This work
pSEH93	pSEH81(seh35/36)	pSCrhaB2::plpD312-728StrepII	This work
pSEH95	pSEH81(seh35/37)	pSCrhaB2::plpD332-728StrepII	This work
pSEH96	pSEH81(seh35/38)	pSCrhaB2::plpD406-728StrepII	This work
pSEH112	pSEH81(seh52/53)	pSCrhaB2::plpD(L703C)StrepII	This work
pSEH114	pSEH81(seh56/57)	pSCrhaB2::plpD(I117C)StrepII	This work
pSEH128	pSEH112(seh54/55)	pSCrhaB2::plpD(L703C,V113C)StrepII	This work
pSEH129	pSEH114(seh58/59)	pSCrhaB2::plpD(I117C,L411C)StrepII	This work
pSEH170	pSEH81(seh88/89)	pSCrhaB2::plpD(L359C)StrepII	This work
pSEH171	pSEH81(seh90/91)	pSCrhaB2::plpD(V146C)StrepII	This work
pSEH172	pSEH81(seh96/97)	pSCrhaB2::plpD(Q383C)StrepII	This work
pSEH173	pSEH81(seh98/99)	pSCrhaB2::plpD(S118C)StrepII	This work
pSEH176	pSEH114(seh113/114)	pSCrhaB2::plpD(I117C,L413C)StrepII	This work
pSEH184	pSEH114(seh109/110)	pSCrhaB2::plpD(I117C,F410C)StrepII	This work
pSEH185	pSEH81(seh54/55)	pSCrhaB2::plpD(V113C)StrepII	This work

pSEH192	pSEH173(seh58/59)	pSCrhaB2::plpD(S118C,L411C)StrepII	This work
pSEH196	pSEH114(seh119/120)	pSCrhaB2::plpD(I117C,R412C)StrepII	This work
pSEH197	pSEH81(seh123/124)	pSCrhaB2::plpD(M249C)StrepII	This work
pSEH202	pSEH171(seh127/128)	pSCrhaB2::plpD(V146C,V388C)StrepII	This work
pSEH203	pSEH172(seh139/140)	pSCrhaB2::plpD(Q383C,N136C)StrepII	This work
pSEH209	pSEH175(seh149/150)	pSCrhaB2::plpD(N723C,S418C)StrepII	This work
pSEH210	pSEH81(seh151/152)	pSCrhaB2::plpD(F427C)StrepII	This work
pSEH211	pSEH81(seh163/164)	pSCrhaB2::plpD(L145C)StrepII	This work
pSEH212	pSEH114(seh145/146)	pSCrhaB2::plpD(I117C,L415C)StrepII	This work
pSEH213	pSEH81(seh155/156)	pSCrhaB2::plpD(I532C)StrepII	This work
pSEH221	pSEH81(seh159/160)	pSCrhaB2::plpD(N513C)StrepII	This work
pSEH222	pSEH81(seh161/162)	pSCrhaB2::plpD(F102C)StrepII	This work
pSEH233	pSEH222(seh159/160)	pSCrhaB2::plpD(F102C,N513C)StrepII	This work
pSEH238	pSEH172(seh165/166)	pSCrhaB2::plpD(Q383C,Q135C)StrepII	This work
pSEH240	pSEH81(seh145/146)	pSCrhaB2::plpD(L415C)StrepII	This work
pSEH242	pSEH81(seh149/150)	pSCrhaB2::plpD(S418C)StrepII	This work
pSEH249	pSEH81(seh176/177)	pSCrhaB2::plpD(F410C)StrepII	This work
pSEH250	pSEH81(seh180/181)	pSCrhaB2::plpD(R412C)StrepII	This work
pSEH251	pSEH81(seh182/183)	pSCrhaB2::plpD(L413C)StrepII	This work
pSEH264	pSEH211(178/179)	pSCrhaB2::plpD(L145C,V388C)StrepII	This work
pSEH266	pSEH81(seh171/172)	pSCrhaB2::plpD(Q135C)StrepII	This work
pSEH306	pSEH81(seh206/207)	pSCrhaB2::plpD	This work
pSEH318	pSEH306(seh88/89)	pSCrhaB2::plpD(L359C)	This work
pSEH320	pSEH306(seh117/118)	pSCrhaB2::plpD(M249C)	This work
pSEH322	pSEH306(157/158)	pSCrhaB2::plpD(F427C)	This work

Supplementary Table 1: Plasmids used in this study.

Tm^R= Trimethoprim resistance, Cm^R= Chloramphenicol resistance, Gm^R= Gentamicin resistance

Name	Sequence	Notes
mtd350	TACTCCGTATTCAAAAGGGCCTGC	F, primer for linearizing pMTD607
mtd351	CGCAAAAGAATATGAGGATTGTAATAAGC	R, primer to linearizing pMTD607
seh4	CCCGCCCTTTTCAAATTGAGG	R, primer for truncating PlpD
seh5	AAACCAGTAATCGACGCTATTCG	F, primer for truncating linker off PlpD
seh7	GGTGGGACAGACTTTCTGCG	F, primer for truncating POTRA off PlpD
seh13	CGCAAAAGAATATGAGGATTGTAATAAG	F, primer for adding first two residues of PlpD PL-domain and removing StrepII tag
seh14	GAGGCCCGGCCAAAGATC	R, primer for adding first two residues of PlpD PL-domain and removing StrepII tag
seh35	GCCGCCTTTTTCGAACTGG	R, primer for truncating PlpD
seh36	CGCAAGCCGAAGGACCTC	F, primer for truncating passenger off PlpD
seh37	AAGCCGGTCATCGACGC	F, primer for truncating linker off PlpD
seh38	GGCGGCACCGACTTCCT	F, primer for truncating POTRA off PlpD
seh52	TGCGGGCCCCTCACCTTCAGCTATGG	F, NQ5 primer for L703C substitution
seh53	CGGCGTATCGAAGCCGATCATCAGGC	R, NQ5 primer for L703C substitution
seh54	TGCAAGCAGAAGATCAGCTTCCGCGA	F, NQ5 primer for V113C substitution
seh55	CAGGAAGTCGCGGTCGTCCTGCTTG	R, NQ5 primer for V113C substitution
seh56	GCACTTCTGCTTGACCAGGAAGTCGCG	R, NQ5 primer for I117C substitution
seh57	AGCTTCCGCGACGACGGCACCCT	F, NQ5 primer for I117C substitution
seh58	TGCCGCCTCGGCCTCAACCTGTCC	F, NQ5 primer for L411C substitution

seh59	GAAGTCGGTGCCGCCTTTCTTGCCGG	R, NQ5 primer for L411C substitution
seh88	GCACGGCTGCCGGATGTAGTGGCGGATCAC	R, NQ5 primer for L359C substitution
seh89	GGCACGCGCCTCGATCTCGGCCG	F, NQ5 primer for L359C substitution
seh90	TGCCACACCAGCGACAACCGCGACTTCGACAA	F, NQ5 primer for V146C substitution
seh91	CAGCAGGGATTCCAGCACCATCGCCAGGTTCTG	R, NQ5 primer for V146C substitution
seh96	TGCGTGCAGTACCGGGTGGTCAAGGAAAAGAAGC	F, NQ5 primer for Q383C substitution
seh97	GTCGAAGTAATCGAGGCCATAGAGGGTGCTCATGTC	R, NQ5 primer for Q383C substitution
seh98	GCAGATCTTCTGCTTGACCAGGAAGTCGCGGTC	R, NQ5 primer for S118C substitution
seh99	TTCCGCGACGACGGCACCCTCGGCC	F, NQ5 primer for S118C substitution
seh109	TGCCTTCGCCTCGGCCTCAACCTGTCCGAT	F, NQ5 primer for F410C substitution
seh110	GTCGGTGCCGCCTTTCTTGCCGGTGGCATG	R, NQ5 primer for F410C substitution
seh113	TGCGGCCTCAACCTGTCCGATGACATGCGC	F, NQ5 primer for L413C substitution
seh114	GCGAAGGAAGTCGGTGCCGCCTTTCTTGCC	R, NQ5 primer for L413C substitution
seh117	TGCAACCAGTCGATCACCCTCATGACCCGGAAGAAC	F, NQ5 primer for M249C substitution
seh118	CACGTCGAGCACGGTCGAGAGGTCCTTGC	R, NQ5 primer for M249C substitution
seh119	GGCGGCACCGACTTCCTTTGCCTCGGCCTCAACCTGTCCGATG	F, QC primer for R412C substitution
seh120	CATCGGACAGGTTGAGGCCGAGGCAAAGGAAGTCGGTGCCGCC	R, QC primer for R412C substitution
seh123	GCTCGACGTGTGCAACCAGTCGATCACCCTCATG	F, QC primer for M249C substitution
seh124	CATGAGGGTGATCGACTGGTTGCACACGTCGAGC	R, QC primer for M249C substitution
seh127	CGACCAGGTGCAGTACCGGTGCGTCAAGGAAAAGAAGCTCAACAC	F, QC primer for V388C substitution
seh128	GTGTTGAGCTTCTTTTCCTTGACGCACCGGTACTGCACCTGGTCG	R, QC primer for V388C substitution
seh139	GGTGATCCAGGGCCAGTGCCTGGCGATGGTGC	F, QC primer for N136C substitution
seh140	GCACCATCGCCAGGCACTGGCCCTGGATCACC	R, QC primer for N136C substitution
seh145	TCGCCTCGGCTGCAACCTGTCCG	F, NQ5 primer for L415C substitution
seh146	AGGAAGTCGGTGCCGCCT	R, NQ5 primer for L415C substitution

seh149	CCTCAACCTGTGCGATGACATGC	F, NQ5 primer for S418C substitution
seh150	CCGAGGCGAAGGAAGTCG	R, NQ5 primer for S418C substitution
seh151	CGAAAGCACCTGCAACCTCGGCG	F, NQ5 primer for F427C substitution
seh152	CCGCGCATGTCATCGGAC	R, NQ5 primer for F427C substitution
seh155	GGACGTACGCTGCGGCGACCCGTC	F, NQ5 primer for I532C substitution
seh156	GCCTTGCCGTAGGCCTGCACCGC	R, NQ5 primer for I532C substitution
seh157	CGAAAGCACCTGCAACCTCGGCG	F, NQ5 primer for F427C substitution
seh158	CCGCGCATGTCATCGGACAGGTTG	R, NQ5 primer for F427C substitution
seh159	GCAGATCGCCTGCAACGGCGAAATCCGGC	F, NQ5 primer for N513C substitution
seh160	CGGCCCACGTTGAGGCCGTAGCCGTAGC	R, NQ5 primer for N513C substitution
seh161	GGACGTGCCGTGCCGGCGCAAGC	F, NQ5 primer for F102C substitution
seh162	TTGCGCGGGGGCGCGTCGGACA	R, NQ5 primer for F102C substitution
seh163	GGAATCCCTGTGCGTCCACACCAGCGACAAC	F, NQ5 primer for L145C substitution
seh164	AGCACCATCGCCAGGTTCTGGCCCTG	R, NQ5 primer for L145C substitution
seh165	GATCCAGGGCTGCAACCTGGCGATGGTGCTGG AATCC	F, NQ5 primer for Q135C substitution
seh166	ACCCCCAGCGGCAGGCCGAGGGTGCCG	R, NQ5 primer for Q135C substitution
seh171	GATCCAGGGCTGCAACCTGGCGATGGTG	F, NQ5 primer for Q135C substitution
seh172	ACCCCCAGCGGCAGGCCG	R, NQ5 primer for Q135C substitution
seh176	CGGCACCGACTGCCTTCGCCTCG	F, NQ5 primer for F410C substitution
seh177	CCTTTCTTGCCGGTGGCATGGATGACCAGG	R, NQ5 primer for F410C substitution
seh178	GCAGTACCGGTGCGTCAAGGAAAAGAAG	F, NQ5 primer for V388C substitution
seh179	ACCTGGTCGAAGTAATCGAGGCCATAGAG	R, NQ5 primer for V388C substitution
seh180	CCGACTTCCTTTGCCTCGGCCTCAACC	F, NQ5 primer for R412C substitution
seh181	TGCCGCCTTTCTTGCCGGTGGCAT	R, NQ5 primer for R412C substitution
seh182	CTTCCTTCGCTGCGGCCTCAACC	F, NQ5 primer for L413C substitution

seh183	TCGGTGCCGCCTTTCTTGCCGG	R, NQ5 primer for L413C substitution
seh206	CGTGGCGAGGGCGGACAGGGGAAGC	R, NQ5 primer for removal of TS tag
seh207	GAGGCCCGGCCGAAGATCGGCCTGG	F, NQ5 primer for removal of TS tag

Supplementary Table 2: Oligonucleotides used in this study.

F= forward strand, R= reverse strand, GA= Gibson Assembly, QC= QuikChange, NQ5 = NEB

Q5 site-directed mutagenesis

Name	Sequence	Notes
mtd359	ATGTTTTTTAGGCTTATTACAATCCTCATATTCTTTTGCGAGCGC<br>ATGGAGTCACCCGCAGTTCGAAAAGGGGGGTGGTTCGGGTGG<br>GGGTTCGGGGGGTTCAGCCTGGTCCCATCCTCAATTTGAAAAG<br>GGCGGGCGTAAACCTAAGGACTTAAATTCTGAAGCATTAGATG<br>TCGCCCGGACTCCTAATCAACGCAAACCAGTAATCGACGCTAT<br>TCGGGTGGAGAATAACTCCAAAGTAAGTGATGAAGTGATCCGC<br>CATTATATTCGGCAGCCGCTCGGGACGCGGTTGGATCTTGGTC<br>GCCTGCAGGACGACATGTCGACGTTATATGGGCTCGATTACTT<br>TGATCAAGTACAATATCGGGTGGTCAAGGAGAAGAAACTGAAC<br>ACATTGGTCATCCACGCAACGGGCAAAAAAGGTGGGACAGACT<br>TTCTGCGTCTTGGCTTGAATCTGAGTGATGACATGCGCGGTGA<br>ATCCACCTTCAATTTAGGGGGGTCGTACCGGATGAATGGGCTG<br>AATCGGCTGGGCGCGGAATGGCTGACTCGCGTCCAGCTCGGG<br>GATCGGCAGGAGCTCTACTCCGAATTCTACCAGCCGTTAGACG<br>TCGGCTCGCGCTATTTTGTAGCACCTTTCCTTTTTCACGAGGCA<br>CAAAATGTGGACGTGACCGAAGACAATGATCCGTTACTGCGCT<br>ATCGGCTTGAGCGCTATGGTTACGGTCTGAACGTAGGTCGCCA<br>AATCGCCAACAATGGTGAAATTCGCTTGGGTGCAGTCCAAGCG<br>TACGGCAAGGCTGATGTCCGGATTGGTGACCCGAGCCTCCCT<br>GATATTGATTTCACCGAAGGTTACTATGAATTAAAGTACTCTTTC<br>GATACGGTAGACGACGTCAACTTTCCGCATGAGGGGGAGGAG<br>ATTGGGTTAACTATGCGCCGGTATGATAAGAGTCTCGGGAGCG<br>ATGATTCTTACCGCCAATGGGATTTACGCCTTAATAAAGCGCTG<br>TCGTTCGGCGCTGACACATGGGTGTTTGGTGGTGGTTATGGTC<br>GCACTCTGGACGACGCGGAGGTCGTAACTAGCTCATTCACGTT<br>GGGGGGCGCTCGGGAACTCAGCGGCTTCCGCCGCGATGCGT<br>TGTCGGGGCAAAACTACAGCCTCGGGCGTATTGTGTATTACCG<br>GCGTCTCACAGAACGCTCCTTCTTGCCACTTGATTTCCCGTTAT<br>ATTTAGGTGGTTCTATTGAGCGGGGCCGCATTTGGAACAATGA<br>TAATGAGTACGATTCGGGTTATATTAATGCGGCATCCCTTATGA<br>TTGGTTTTGACACCCCACTGGGGCCACTCACGTTTTCTTATGG<br>GATCAATGACGAGAACTTTAAAGCATTCTACCTTAATTTAGGTC<br>AGAATTTCTGATACTCCGTATTCAAAAGGGCCTGCGGGCCCTT<br>TACGCTCA	Fragment containing $PlpD_{312-728}$ with TS tag codon-optimized for *E. coli*, to be ligated into pMTD607

mtd372	CGGCCAAAGATCGGTTTGGTACTGTCCGGTGGTGCAGCACGT<br>GGGTTAGCCCACATCGGCGTCTTGAAGGCACTGGACGAACAA<br>GGGATCCAAATCGACGCTATTGCAGGCACATCAATGGGCGCG<br>GTCGTTGGGGGTCTCTATGCGTCAGGGTATACTCCTGCTGAAT<br>TAGAACGTATCGCGTTGGAAATGGACTGGCAACAAGCGTTGAG<br>TGATGCACCTCCGCGGAAGGATGTACCATTCCGCCGTAAACAG<br>GACGATCGCGATTTCCTTGTGAAACAAAAGATCTCCTTCCGTG<br>ACGACGGGACACTCGGTTTGCCACTCGGGGTGATCCAGGGCC<br>AAAATCTCGCTATGGTACTGGAGTCCCTCTTGGTTCATACATCC<br>GACAATCGGGATTTCGATAAGCTTGCCATCCCTTTCCGCGCAG<br>TTTCAACGGACATTGCTACAGGCGAAAAGGTCGTTTTTCGCAA<br>AGGTCATCTGCCGCAAGCAATCCGCGCCTCGATGTCCATTCCA<br>GCGGTTTTTGCGCCGGTTGAGATTGATGGCCGCCTTCTTGTGG<br>ATGGTGGTATGGTAGACAACATTCCAGTTGATGTAGCTCGGGA<br>CATGGGCGTAGATGTCGTAATTGTCGTTGATATCGGTAACCCT<br>CTCCGCGACCGGAAGGATCTGTCCACCGTACTCGACGTAATGA<br>ACCAATCGATTACCCTGATGACCCGCAAGAATAGCGAGGCTCA<br>ATTAGCGACCTTGAAACCTGGCGACGTATTGATCCAACCTCCT<br>CTGTCCGGCTATGGTACAACAGACTTTGGTCGCGTGCCACAAC<br>TGATCGATGCGGGCTACCGGGCAACTACGGTTCTTGCCGCTC<br>GCCTGGCCGAATTG	Fragment containing $PlpD_{20-311}$ codon-optimized for *E. coli*, to be ligated into pMTD1523
seh15	GAATTCAGGCGCTTTTTAGACTGGTCGTAATGAAATTCAGCAG<br>GATCACATATGCGCCGCCTGCTGCTCGTACTGCTGCTCCTGCT<br>TCCCCTGTCCGCCCTCGCCACGTCGGCCTGGTCCCATCCGCA<br>GTTCGAAAAGGGTGGCGGCAGCGGCGGTGGCTCGGGCGGCA<br>GCGCGTGGAGCCATCCCCAGTTCGAAAAAGGCGGCGAGGCCC<br>GGCCGAAGATCGGCCTGGTCCTCTCCGGAGGTGCGGCCCGT<br>GGCCTGGCCCATATCGGCGTGCTCAAGGCCCTCGACGAACAG<br>GGCATCCAGATCGACGCCATCGCGGGCACCAGCATGGGCGC<br>GGTGGTCGGCGGCCTGTACGCCTCCGGCTACACCCCCGCGG<br>AACTGGAGCGCATCGCCCTGGAGATGGACTGGCAGCAGGCGC<br>TGTCCGACGCGCCCCCGCGCAAGGACGTGCCGTTCCGGCGC<br>AAGCAGGACGACCGCGACTTCCTGGTCAAGCAGAAGATCAGC<br>TTCCGCGACGACGGCACCCTCGGCCTGCCGCTGGGGGTGATC<br>CAGGGCCAGAACCTGGCGATGGTGCTGGAATCCCTGCTGGTC<br>CACACCAGCGACAACCGCGACTTCGACAAGCTGGCGATCCCC<br>TTCCGCGCCGTCTCCACCGATATCGCAACCGGCGAGAAAGTG<br>GTGTTCCGCAAGGGCCACCTGCCCCAGGCGATCCGCGCCTCG<br>ATGTCGATCCCCGCCGTGTTCGCCCCGGTCGAAATCGACGGC<br>AGGCTGCTGGTGGATGGCGGCATGGTCGATAACATCCCGGTG<br>GACGTCGC	Fragment combined with seh16 to contain PlpD gBlock for cloning TS-tagged $PlpD_{1-728}$ codon-optimized for *P. aeruginosa* PA14, to be ligated into pSCrhaB2

seh16	AGGCTGCTGGTGGATGGCGGCATGGTCGATAACATCCCGGTG<br>GACGTCGCGCGCGACATGGGCGTGGATGTCGTGATCGTGGTC<br>GACATCGGCAACCCCCTGCGCGACCGCAAGGACCTCTCGACC<br>GTGCTCGACGTGATGAACCAGTCGATCACCCTCATGACCCGGA<br>AGAACTCCGAAGCCCAGCTGGCCACGCTCAAGCCCGGCGACG<br>TGCTGATCCAGCCGCCGCTCTCCGGCTATGGCACCACCGATTT<br>CGGCCGCGTCCCGCAACTGATCGACGCCGGCTACCGGGCCA<br>CCACGGTGCTCGCCGCGCGCCTCGCCGAGTTGCGCAAGCCG<br>AAGGACCTCAACAGCGAAGCGCTCGACGTGGCGCGCACGCCG<br>AACCAGCGCAAGCCGGTCATCGACGCGATCCGCGTGGAAAAC<br>AACTCGAAGGTCAGCGACGAGGTGATCCGCCACTACATCCGG<br>CAGCCGCTCGGCACGCGCCTCGATCTCGGCCGCCTCCAGGAC<br>GACATGAGCACCCTCTATGGCCTCGATTACTTCGACCAGGTGC<br>AGTACCGGGTGGTCAAGGAAAAGAAGCTCAACACCCTGGTCAT<br>CCATGCCACCGGCAAGAAAGGCGGCACCGACTTCCTTCGCCT<br>CGGCCTCAACCTGTCCGATGACATGCGCGGCGAAAGCACCTT<br>CAACCTCGGCGGCAGCTACCGCATGAACGGCCTCAACCGGCT<br>CGGCGCGGAGTGGCTCACCCGGGTCCAGCTCGGCGATCGCC<br>AGGAACTCTATTCGGAATTCTATCAGCCCCTCGACGTCGGCTC<br>ACGGTACTTCGTCGCGCCCTTCCTCTTCCACGAAGCACAGAAC<br>GTCGACGTCACCGAGAACAACGACCCGCTGCTGCGCTATCGC<br>CTGGAGCGCTACGGCTACGGCCTCAACGTGGGCCGGCAGATC<br>GCCAACAACGGCGAAATCCGGCTGGGCGCGGTGCAGGCCTAC<br>GGCAAGGCGGACGTACGCATCGGCGACCCGTCCCTCCCGGA<br>CATCGACTTCACCGAAGGCTACTACGAACTGAAGTACTCGTTC<br>GACACCGTCGACGACGTGAATTTCCCCCACGAGGGCGAGGAA<br>ATCGGCCTGACCATGCGCCGCTACGACAAGTCCCTCGGCTCG<br>GATGACTCCTACCGGCAGTGGGACCTGCGGCTGAACAAGGCG<br>CTCAGCTTCGGCGCCGACACCTGGGTGTTCGGCGGCGGCTAC<br>GGGCGGACCCTGGACGACGCCGAAGTGGTCACCTCCAGCTTC<br>ACCCTCGGCGGCGCCCGCGAACTGTCGGGCTTCCGCCGCGAT<br>GCGCTGTCCGGGCAGAACTACAGCCTGGGGCGGATCGTCTAT<br>TACCGGCGCCTCACCGAACGTTCCTTCCTGCCGCTGGACTTCC<br>CGCTGTACCTGGGCGGCTCCATCGAACGCGGACGCATCTGGA<br>ACAACGACAACGAATACGACTCGGGCTACATCAACGCCGCCA<br>GCCTGATGATCGGCTTCGATACGCCGCTGGGGCCCCTCACCT<br>TCAGCTATGGGATCAACGACGAAAACTTCAAGGCGTTCTACCT<br>GAACCTCGGGCAGAACTTCTGAATGCCATGGTACTCCGTATTC<br>AAAAGGGCCTGCGGGCCCTTTACGCTCAG	Fragment combined with seh15 to contain PlpD gBlock for cloning TS-tagged $PlpD_{1-728}$ codon-optimized for *P. aeruginosa* PA14, to be ligated into pSCrhaB2

seh23	CAATGTAACATCAGAGATTTTGAGACACGGGCCAGAGCTGCAG CTGGATGGCGCAGCGGTTCAGCGGGAAGGACGATCGTTGTGC CCGAGGTCGCGTTGCGGGTCGATGCGGTCGCGAACCCGCTG CTTGAGCGCCTTGGCCTCGGGGAAGCCGCCGTCGGCCTTGCG CTCCCAGACCTGCACGCCGTCGCAGGTGATACGGAAGACGCC ACCGGTTCCGGGCTCCAGGCAGACTTTTCCGAGATCGTCGGC GAAGGTGCTGAGCAGTTCCTGGGCCAGCCAGGCGGCACGCA GTAGCCACTGGCACTGGGTGCAATAGGTGATGACGATTTCGG GCTTGGCGGTGGGCATGTCGGCGGCTCAGGGGAAACGGTCG CGCCTATAATAGCCGCCTTTTCACCCGGCTCCGGATCGTTCTC GAACTACAGCCTGGGGCGGATCGTCTATTACCGGCGCCTCAC CGAACGTTCCTTCCTGCCGCTGGACTTCCCGCTGTACCTGGGC GGCAGCATCGAGCGCGGACGCATCTGGAACAACGACAACGAA TACGACAGCGGCTACATCAACGCCGCCAGCCTGATGATCGGC TTCGATACGCCGCTGGGGCCGCTGACCTTCAGCTACGGGATC AACGACGAGAACTTCAAGGCGTTCTACCTGAACCTCGGGCAGA ACTTCTGAGCGAAGTTCAGGCGATCAGCGCCAGCAACTCGCG CGCCTCGGCCTTCTGCCACGGGTCGCCGTGCGCCACCACCAG GCGCAGGATGCGGCTGGCCTGGTCGAGATCGCCGCTGTCGAT GCAGGCGCGGGCCCGGTCCAGGGGCCCGAGATCCATGCTAG CGTTAACAAGCTTGCATGCCTGCAGGTCG	Fragment containing partial upstream and downstream sequence of *P. aeruginosa* chromosomal plpD gene, to be ligated into pDONRpEX18Gm

Supplementary Table 3: dsDNA fragments used in this study.

F=forward strand, R=reverse strand, TS=TwinStrepII

ANOVA table	SS	DF	MS	F (DFn, DFd)	P-value
Treatment (between columns)	34666	7	4952	F (7, 16) = 141.2	P<0.0001
Residual (within columns)	561.2	16	35.08		
Total	35227	23			
**Tukey's multiple comparisons test**	**Adjusted P**				
Q383 C vs. N136, Q383 C	<0.0001				
Q383 C vs. Q135 C	>0.9999				
Q383 C vs. Q135, Q383 C	<0.0001				
Q383 C vs. L145 C	>0.9999				
Q383 C vs. L145, V388 C	<0.0001				
Q383 C vs. V146 C	>0.9999				
Q383 C vs. V146, V388 C	<0.0001				
N136, Q383 C vs. Q135 C	<0.0001				
N136, Q383 C vs. Q135, Q383 C	<0.0001				
N136, Q383 C vs. L145 C	<0.0001				
N136, Q383 C vs. L145, V388 C	0.0648				
N136, Q383 C vs. V146 C	<0.0001				
N136, Q383 C vs. V146, V388 C	<0.0001				
Q135 C vs. Q135, Q383 C	<0.0001				
Q135 C vs. L145 C	>0.9999				
Q135 C vs. L145, V388 C	<0.0001				
Q135 C vs. V146 C	>0.9999				
Q135 C vs. V146, V388 C	<0.0001				
Q135, Q383 C vs. L145 C	<0.0001				
Q135, Q383 C vs. L145, V388 C	0.0086				
Q135, Q383 C vs. V146 C	<0.0001				
Q135, Q383 C vs. V146, V388 C	0.2459				
L145 C vs. L145, V388 C	<0.0001				
L145 C vs. V146 C	>0.9999				
L145 C vs. V146, V388 C	<0.0001				
L145, V388 C vs. V146 C	<0.0001				
L145, V388 C vs. V146, V388 C	<0.0001				
V146 C vs. V146, V388 C	<0.0001				

Supplementary Table 4: One-sided ANOVA and multiple comparison test for catalyzed disulfide bond formation assays shown in Figs. 3b and c (catalyzed samples only).

SS=sum of squares, DF=degrees of freedom, MS=mean square

Catalyzed:					
**ANOVA table**	**SS**	**DF**	**MS**	**F (DFn, DFd)**	**P-value**
Treatment (between columns)	7742	3	2581	F (3, 8) = 434.3	P<0.0001
Residual (within columns)	47.53	8	5.942		
Total	7789	11			
**Tukey's multiple comparisons test**	**Adjusted P**				
L359 C vs. M249 C	<0.0001				
L359 C vs. F427 C	0.0104				
L359 C vs. I532 C	<0.0001				
M249 C vs. F427 C	<0.0001				
M249 C vs. I532 C	0.6677				
F427 C vs. I532 C	<0.0001				
Spontaneous:					
**ANOVA table**	**SS**	**DF**	**MS**	**F (DFn, DFd)**	**P-value**
Treatment (between columns)	1885	3	628.4	F (3, 8) = 32.44	P<0.0001
Residual (within columns)	155	8	19.37		
Total	2040	11			
**Tukey's multiple comparisons test**	**Adjusted P**				
L359 C vs. M249 C	0.779				
L359 C vs. F427 C	0.3797				
L359 C vs. I532 C	0.0005				
M249 C vs. F427 C	0.8719				
M249 C vs. I532 C	0.0002				
F427 C vs. I532 C	0.0001				

Supplementary Table 5: One-sided ANOVA and multiple comparison test for disulfide bond formation assays shown in Figs. 4c and d

SS=sum of squares, DF=degrees of freedom, MS=mean square

One-phase association	
**Best-fit values**	
Y0	50.21
Plateau	63.46
K	0.8738
Tau	1.144
Half-time	0.7932
Span	13.25
**95% CI (profile likelihood)**	
Y0	43.45 to 56.94
Plateau	60.71 to 66.28
K	0.2401 to ???
Tau	??? to 4.165
Half-time	??? to 2.887
**Goodness of Fit**	
Degrees of Freedom	21
R squared	0.4023
Sum of Squares	668.6
Sy.x	5.642
**Constraints**	
K	K > 0
**Number of points**	
# of X values	24
# Y values analyzed	24

Supplementary Table 6: Nonlinear analysis of graph shown in Fig. 4e.

One-phase association	
**Best-fit values**	
Y0	9.582
Plateau	91.5
K	0.05077
Tau	19.7
Half-time	13.65
Span	81.92
**95% CI (profile likelihood)**	
Y0	6.203 to 12.92
Plateau	87.61 to 95.76
K	0.04273 to 0.05989
Tau	16.70 to 23.40
Half-time	11.57 to 16.22
**Goodness of Fit**	
Degrees of Freedom	18
R squared	0.9875
Sum of Squares	258.6
Sy.x	3.791
**Constraints**	
K	K > 0
**Number of points**	
# of X values	21
# Y values analyzed	21

Supplementary Table 7: Nonlinear analysis of graph shown in Fig. 4f.

ANOVA table	SS	DF	MS	F (DFn, DFd)	P-value
Treatment (between columns)	53842	17	3167	F (17, 36) = 298.4	P<0.0001
Residual (within columns)	382.1	36	10.61		
Total	54225	53			
**Tukey's multiple comparisons test**	**Adjusted P**				
I117 C vs. F410 C	>0.9999				
I117 C vs. R412 C	>0.9999				
I117 C vs. L413 C	>0.9999				
I117 C vs. L415 C	>0.9999				
I117 C vs. I117,F410 C	<0.0001				
I117 C vs. I117,L411 C	<0.0001				
I117 C vs. I117,R412 C	<0.0001				
I117 C vs. I117,L413 C	<0.0001				
I117 C vs. I117,L415 C	<0.0001				
I117 C vs. S118 C	>0.9999				
I117 C vs. S118,L411 C	<0.0001				
I117 C vs. V113 C	>0.9999				
I117 C vs. L703 C	>0.9999				
I117 C vs. V113,L703 C	<0.0001				
I117 C vs. F102 C	>0.9999				
I117 C vs. N513 C	>0.9999				
I117 C vs. F102,N513 C	<0.0001				
F410 C vs. R412 C	>0.9999				
F410 C vs. L413 C	>0.9999				
F410 C vs. L415 C	>0.9999				
F410 C vs. I117,F410 C	<0.0001				
F410 C vs. I117,L411 C	<0.0001				
F410 C vs. I117,R412 C	<0.0001				
F410 C vs. I117,L413 C	<0.0001				
F410 C vs. I117,L415 C	<0.0001				
F410 C vs. S118 C	>0.9999				
F410 C vs. S118,L411 C	<0.0001				
F410 C vs. V113 C	>0.9999				
F410 C vs. L703 C	>0.9999				
F410 C vs. V113,L703 C	<0.0001				
F410 C vs. F102 C	>0.9999				

F410 C vs. N513 C	>0.9999				
F410 C vs. F102,N513 C	<0.0001				
R412 C vs. L413 C	>0.9999				
R412 C vs. L415 C	>0.9999				
R412 C vs. I117,F410 C	<0.0001				
R412 C vs. I117,L411 C	<0.0001				
R412 C vs. I117,R412 C	<0.0001				
R412 C vs. I117,L413 C	<0.0001				
R412 C vs. I117,L415 C	<0.0001				
R412 C vs. S118 C	>0.9999				
R412 C vs. S118,L411 C	<0.0001				
R412 C vs. V113 C	>0.9999				
R412 C vs. L703 C	>0.9999				
R412 C vs. V113,L703 C	<0.0001				
R412 C vs. F102 C	>0.9999				
R412 C vs. N513 C	>0.9999				
R412 C vs. F102,N513 C	<0.0001				
L413 C vs. L415 C	>0.9999				
L413 C vs. I117,F410 C	<0.0001				
L413 C vs. I117,L411 C	<0.0001				
L413 C vs. I117,R412 C	<0.0001				
L413 C vs. I117,L413 C	<0.0001				
L413 C vs. I117,L415 C	<0.0001				
L413 C vs. S118 C	>0.9999				
L413 C vs. S118,L411 C	<0.0001				
L413 C vs. V113 C	>0.9999				
L413 C vs. L703 C	>0.9999				
L413 C vs. V113,L703 C	<0.0001				
L413 C vs. F102 C	>0.9999				
L413 C vs. N513 C	>0.9999				
L413 C vs. F102,N513 C	<0.0001				
L415 C vs. I117,F410 C	<0.0001				
L415 C vs. I117,L411 C	<0.0001				
L415 C vs. I117,R412 C	<0.0001				
L415 C vs. I117,L413 C	<0.0001				
L415 C vs. I117,L415 C	<0.0001				
L415 C vs. S118 C	>0.9999				
L415 C vs. S118,L411 C	<0.0001				
L415 C vs. V113 C	>0.9999				

L415 C vs. L703 C	>0.9999				
L415 C vs. V113,L703 C	<0.0001				
L415 C vs. F102 C	>0.9999				
L415 C vs. N513 C	>0.9999				
L415 C vs. F102,N513 C	<0.0001				
I117,F410 C vs. I117,L411 C	<0.0001				
I117,F410 C vs. I117,R412 C	<0.0001				
I117,F410 C vs. I117,L413 C	<0.0001				
I117,F410 C vs. I117,L415 C	<0.0001				
I117,F410 C vs. S118 C	<0.0001				
I117,F410 C vs. S118,L411 C	<0.0001				
I117,F410 C vs. V113 C	<0.0001				
I117,F410 C vs. L703 C	<0.0001				
I117,F410 C vs. V113,L703 C	<0.0001				
I117,F410 C vs. F102 C	<0.0001				
I117,F410 C vs. N513 C	<0.0001				
I117,F410 C vs. F102,N513 C	0.1781				
I117,L411 C vs. I117,R412 C	0.3081				
I117,L411 C vs. I117,L413 C	0.0161				
I117,L411 C vs. I117,L415 C	>0.9999				
I117,L411 C vs. S118 C	<0.0001				
I117,L411 C vs. S118,L411 C	<0.0001				
I117,L411 C vs. V113 C	<0.0001				
I117,L411 C vs. L703 C	<0.0001				
I117,L411 C vs. V113,L703 C	0.275				
I117,L411 C vs. F102 C	<0.0001				
I117,L411 C vs. N513 C	<0.0001				
I117,L411 C vs. F102,N513 C	<0.0001				
I117,R412 C vs. I117,L413 C	0.9964				
I117,R412 C vs. I117,L415 C	0.0961				
I117,R412 C vs. S118 C	<0.0001				
I117,R412 C vs. S118,L411 C	<0.0001				
I117,R412 C vs. V113 C	<0.0001				
I117,R412 C vs. L703 C	<0.0001				
I117,R412 C vs. V113,L703 C	>0.9999				
I117,R412 C vs. F102 C	<0.0001				
I117,R412 C vs. N513 C	<0.0001				
I117,R412 C vs. F102,N513 C	<0.0001				
I117,L413 C vs. I117,L415 C	0.0032				

I117,L413 C vs. S118 C	<0.0001				
I117,L413 C vs. S118,L411 C	<0.0001				
I117,L413 C vs. V113 C	<0.0001				
I117,L413 C vs. L703 C	<0.0001				
I117,L413 C vs. V113,L703 C	0.9979				
I117,L413 C vs. F102 C	<0.0001				
I117,L413 C vs. N513 C	<0.0001				
I117,L413 C vs. F102,N513 C	<0.0001				
I117,L415 C vs. S118 C	<0.0001				
I117,L415 C vs. S118,L411 C	<0.0001				
I117,L415 C vs. V113 C	<0.0001				
I117,L415 C vs. L703 C	<0.0001				
I117,L415 C vs. V113,L703 C	0.0828				
I117,L415 C vs. F102 C	<0.0001				
I117,L415 C vs. N513 C	<0.0001				
I117,L415 C vs. F102,N513 C	<0.0001				
S118 C vs. S118,L411 C	<0.0001				
S118 C vs. V113 C	>0.9999				
S118 C vs. L703 C	>0.9999				
S118 C vs. V113,L703 C	<0.0001				
S118 C vs. F102 C	>0.9999				
S118 C vs. N513 C	>0.9999				
S118 C vs. F102,N513 C	<0.0001				
S118,L411 C vs. V113 C	<0.0001				
S118,L411 C vs. L703 C	<0.0001				
S118,L411 C vs. V113,L703 C	<0.0001				
S118,L411 C vs. F102 C	<0.0001				
S118,L411 C vs. N513 C	<0.0001				
S118,L411 C vs. F102,N513 C	0.0099				
V113 C vs. L703 C	>0.9999				
V113 C vs. V113,L703 C	<0.0001				
V113 C vs. F102 C	>0.9999				
V113 C vs. N513 C	>0.9999				
V113 C vs. F102,N513 C	<0.0001				
L703 C vs. V113,L703 C	<0.0001				
L703 C vs. F102 C	>0.9999				
L703 C vs. N513 C	>0.9999				
L703 C vs. F102,N513 C	<0.0001				
V113,L703 C vs. F102 C	<0.0001				

V113,L703 C vs. N513 C	<0.0001				
V113,L703 C vs. F102,N513 C	<0.0001				
F102 C vs. N513 C	>0.9999				
F102 C vs. F102,N513 C	<0.0001				
N513 C vs. F102,N513 C	<0.0001				

Supplementary Table 8: One-sided ANOVA and multiple comparison test for disulfide bond formation assays shown in Fig. 5b (catalyzed samples only).

SS=sum of squares, DF=degrees of freedom, MS=mean square

Putative ID	Response	Fold change	Welch P-value
Carnitine	↓ΔplpD	0.92	0.56691
CAR 16:0	↓ΔplpD	0.86	0.94864
CL 57:2	↑ΔplpD	2.28	0.00326
CL 66:0	↑ΔplpD	1.48	0.00159
CL 66:2	↑ΔplpD	1.53	0.05592
CL 74:0	↑ΔplpD	1.89	0.02104
CL 74:3	↑ΔplpD	1.17	0.26626
CL 74:4	↑ΔplpD	1.44	0.08121
CL 76:4	↑ΔplpD	1.79	0.00024
CL 76:7	↑ΔplpD	1.33	0.07420
CL 78:1	↑ΔplpD	1.62	0.03218
DG 20:0	↑ΔplpD	1.47	0.00129
DG 32:0	↑ΔplpD	1.16	0.11429
DG 33:1	↓ΔplpD	0.11	0.20042
DG 34:2	↑ΔplpD	1.55	0.00093
DG 34:4	↑ΔplpD	1.50	0.00752
DG 36:0	↑ΔplpD	1.08	0.57456
DG 36:1	↑ΔplpD	1.20	0.27162
DG 36:3	↓ΔplpD	0.98	0.81890
DG 36:4	↑ΔplpD	1.22	0.14237
DG 38:3	↓ΔplpD	0.94	0.81118
DG 38:5	↑ΔplpD	1.42	0.03409
DG 40:10	↑ΔplpD	1.23	0.19989
DG 41:3	↑ΔplpD	1.33	0.07340
DG 42:10	↑ΔplpD	1.55	0.00235
DG 43:3	↑ΔplpD	1.21	0.17578
FA 20:1-O3	↑ΔplpD	1.69	0.00080
FA 10:0-O	↑ΔplpD	1.37	0.01851
FA 11:1-O2	↑ΔplpD	1.72	0.00214
FA 12:0-O	↑ΔplpD	1.54	0.01661
FA 12:1	↑ΔplpD	1.35	0.07410
FA 12:1-O	↑ΔplpD	1.06	0.72895
FA 12:1-O iso n	↑ΔplpD	1.18	0.59856
FA 14:1	↑ΔplpD	1.74	0.13610
FA 16:2	↑ΔplpD	1.50	0.52640
FA 18:0	↑ΔplpD	1.46	0.03014
FA 18:0-O	↑ΔplpD	1.63	0.00368
FA 18:1 iso n	↑ΔplpD	1.49	0.02395
FA 18:1 iso1 p	↑ΔplpD	1.43	0.16780
FA 18:1 iso2 p	↓ΔplpD	0.99	0.83916
FA 18:1-O iso1	↑ΔplpD	1.56	0.00055
FA 18:1-O iso2	↑ΔplpD	1.56	0.04386
FA 18:1-O iso3	↑ΔplpD	1.52	0.03598
FA 18:1-O2	↑ΔplpD	1.58	0.18124
FA 18:1-O2 iso	↑ΔplpD	1.39	0.01163
FA 18:2	↑ΔplpD	2.16	0.03335
FA 18:3	↑ΔplpD	1.55	0.44072

FA 18:3 iso	↑ΔplpD	1.39	0.71971
FA 20:4-O2 iso1 n	↑ΔplpD	1.62	0.01183
FA 20:4-O2 iso1 p	↑ΔplpD	1.81	0.47233
FA 20:4-O2 iso2 n	↑ΔplpD	1.84	0.00567
FA 20:4-O2 iso2 p	↑ΔplpD	1.62	0.46572
FA 20:4-O2 iso3 n	↑ΔplpD	1.45	0.02208
FA 20:5-O	↑ΔplpD	1.44	0.01664
LTE3	↑ΔplpD	1.31	0.12415
LTE4	↑ΔplpD	1.95	0.14743
LysoPE 16:0	↓ΔplpD	0.56	0.89304
LysoPE 16:1	↑ΔplpD	1.28	0.06098
LysoPE 17:0	↑ΔplpD	1.39	0.03126
LysoPE 18:0	↑ΔplpD	1.58	0.00468
LysoPE 18:1 iso n	↑ΔplpD	1.25	0.08238
LysoPE 18:1 iso p	↑ΔplpD	1.27	0.27319
LysoPE 18:2 iso p	↑ΔplpD	1.20	0.47635
LysoPE 18:2 iso1 n	↑ΔplpD	1.53	0.07388
LysoPE 18:2 iso2 n	↑ΔplpD	1.11	0.51372
LysoPE 20:4	↑ΔplpD	1.34	0.02625
LysoPE 24:0	↑ΔplpD	1.62	0.07151
LysoPG 16:0	↑ΔplpD	1.13	0.29568
LysoPG 18:0	↑ΔplpD	1.36	0.08626
LysoPG 18:1	↓ΔplpD	0.65	0.05086
LysoPG 19:1	↑ΔplpD	1.20	0.39277
LysoPG 20:5	↑ΔplpD	1.76	0.00045
LysoPS 17:1	↑ΔplpD	1.44	0.06236
LysoPS 19:0	↑ΔplpD	1.31	0.89145
LysoPS 19:1	↑ΔplpD	1.41	0.18983
LysoPS 20:3	↑ΔplpD	1.30	0.03922
LysoPS 24:1 iso n	↑ΔplpD	1.02	0.64171
LysoPS 24:1 iso p	↑ΔplpD	1.49	0.40241
MG 14:1 iso n	↑ΔplpD	2.07	0.00156
MG 14:1 iso p	↑ΔplpD	1.68	0.84339
MG 15:0	↑ΔplpD	1.41	0.01464
MG 15:1	↑ΔplpD	1.45	0.30664
MG 16:1 iso n	↑ΔplpD	1.46	0.02020
MG 16:1 iso p	↑ΔplpD	2.00	0.00923
MG 17:0	↑ΔplpD	1.33	0.03577
MG 18:1	↑ΔplpD	1.33	0.00448
MG 18:1 iso	↑ΔplpD	1.09	0.37030
MG 18:2	↑ΔplpD	2.21	0.00285
MG 19:0	↑ΔplpD	1.35	0.02597
MG 20:0	↑ΔplpD	1.44	0.00914
MG 22:0	↑ΔplpD	1.15	0.63799
NAE 20:4-O	↓ΔplpD	0.31	0.04020
PA 30:1	↑ΔplpD	1.30	0.02879
PA 31:1	↑ΔplpD	1.03	0.68227
PA 33:2	↑ΔplpD	1.73	0.01606

PA 34:1	↑ΔplpD	1.42	0.00275
PA 34:3	↑ΔplpD	1.13	0.29134
PA 34:6	↑ΔplpD	1.13	0.17632
PA 35:0	↑ΔplpD	1.35	0.01117
PA 35:6	↑ΔplpD	1.29	0.00972
PA 36:3	↑ΔplpD	1.13	0.28028
PA 37:3	↑ΔplpD	1.43	0.01732
PA 38:5	↑ΔplpD	1.45	0.00059
PA 38:5 iso	↑ΔplpD	1.14	0.33630
PA 39:0	↑ΔplpD	1.26	0.04344
PA 39:3	↑ΔplpD	1.27	0.05923
PA 39:3 iso	↑ΔplpD	1.19	0.03145
PA 39:5	↑ΔplpD	1.55	0.00879
PA 40:6	↑ΔplpD	1.32	0.09421
PA 41:3	↑ΔplpD	1.31	0.06247
PA 41:5	↑ΔplpD	1.41	0.00169
PA 42:4	↑ΔplpD	1.29	0.02080
PA 43:0	↑ΔplpD	1.43	0.06084
PA 43:4	↑ΔplpD	1.43	0.00332
PA 43:6	↑ΔplpD	1.61	0.00406
PA 44:10	↑ΔplpD	1.50	0.00107
PA O-32:0	↓ΔplpD	0.97	0.73222
PA O-34:0	↓ΔplpD	0.95	0.82027
PA O-35:1	↑ΔplpD	1.08	0.49377
PA P-28:1	↑ΔplpD	1.38	0.00085
PA P-36:4	↑ΔplpD	1.25	0.03997
PA P-38:4	↑ΔplpD	1.48	0.00120
PE 18:0	↑ΔplpD	1.36	0.45957
PE 28:0	↑ΔplpD	1.52	0.00668
PE 28:1	↑ΔplpD	1.30	0.08838
PE 30:0	↑ΔplpD	1.39	0.00394
PE 30:2	↑ΔplpD	1.27	0.10957
PE 31:1	↑ΔplpD	1.47	0.00199
PE 32:0	↑ΔplpD	1.10	0.61112
PE 32:1	↑ΔplpD	1.36	0.00291
PE 32:2	↑ΔplpD	1.40	0.00399
PE 34:2	↑ΔplpD	1.32	0.01189
PE 34:3	↑ΔplpD	1.47	0.00349
PE 34:5	↑ΔplpD	1.23	0.29781
PE 36:5	↑ΔplpD	1.14	0.58177
PE 38:1	↑ΔplpD	1.67	0.26502
PE 38:5	↑ΔplpD	1.36	0.01473
PE 40:4	↑ΔplpD	1.27	0.04529
PE 42:10	↑ΔplpD	1.33	0.02303
PE 44:10	↑ΔplpD	1.04	0.39736
PE O-28:0	↑ΔplpD	2.05	0.11569
PE P-31:1	↑ΔplpD	1.30	0.03579
PE P-36:4	↑ΔplpD	1.19	0.26315

PE P-37:1	↑ΔplpD	1.80	0.03600
PG 30:0	↑ΔplpD	1.51	0.00107
PG 32:0	↑ΔplpD	1.35	0.03293
PG 32:1	↑ΔplpD	1.42	0.00043
PG 32:2	↑ΔplpD	1.49	0.01736
PG 34:1	↑ΔplpD	1.35	0.00266
PG 34:2	↑ΔplpD	1.40	0.00178
PG 36:2	↑ΔplpD	1.90	0.00061
PG 36:6	↑ΔplpD	1.41	0.00547
PS 29:0	↑ΔplpD	2.25	0.13453
PS 33:0	↑ΔplpD	1.64	0.01387
PS 33:1	↑ΔplpD	1.31	0.13000
PS 35:0	↑ΔplpD	1.49	0.00405
PS 35:1	↑ΔplpD	1.51	0.00294
PS 37:1	↑ΔplpD	1.50	0.00467
PS 37:4	↑ΔplpD	1.30	0.07234
PS 37:5	↑ΔplpD	1.48	0.00099
PS 40:4	↑ΔplpD	1.87	0.00464
PS 40:5	↑ΔplpD	2.08	0.09127
PS 41:5	↑ΔplpD	1.93	0.04583
PS 41:6	↑ΔplpD	1.08	0.28972
PS 43:6	↓ΔplpD	0.46	0.07977
PS 43:7	↑ΔplpD	1.84	0.06501
ST 21:3-O2	↑ΔplpD	1.41	0.01034
ST 21:4;O2	↑ΔplpD	1.41	0.03652
TG 30:0	↑ΔplpD	1.84	0.00097
TG 39:0	↑ΔplpD	1.21	0.17887
TG 57:10	↑ΔplpD	1.15	0.82690
TG 66:15	↑ΔplpD	1.36	0.03111

Supplementary Table 9: Lipid differential between strains PA14 and SEH88 (PA14 *ΔplpD*).

An unpaired, parametric Welch two-sided *t*-test was performed to determine the P-values.

Abbreviations: fatty acylcarnitine (CAR); cardiolipin (CL); diacylglycerol (DG); fatty acid (FA); oxidized fatty acid (FA-O); Leukotriene (LTE); monoacylated glycerophospholipid (Lyso); monoacylglycerol (MG); N-acylethanolamine (NAE); phosphatidic acid (PA); phosphatidylethanolamine (PE); phosphatidylglycerol (PG); phosphatidylserine (PS); steroid (ST); triacylglycerol (TG)

Putative ID	Neutral Mass	m/z [ion mode]	Mass Error (mDa)	Retention Time (min)	Alternative IDs
CAR 16:0	399.334	400.3414 [M+H]+	0.0009	9.45	O-palmitoyl-L-carnitine; NAE 21:1-O2
Carnitine	161.105	162.1124 [M+H]+	0.0002	2.18	L-Carnitine
CL 57:2	1250.831	1251.8384 [M+H]+	0.0004	10.42	PIP 57:3-O; PI 64:12-O
CL 66:0	1381.004	1382.0114 [M+H]+	0.0005	10.79	
CL 66:2	1376.971	1377.9784 [M+H]+	0.0012	10.43	
CL 74:0	1493.126	1494.1334 [M+H]+	0.0027	11.59	
CL 74:3	1487.086	1488.0934 [M+H]+	0.0042	11.59	
CL 74:4	1485.07	1486.0774 [M+H]+	0.0039	11.49	
CL 76:4	1513.101	1514.1084 [M+H]+	0.0036	11.61	
CL 76:7	1507.052	1508.0594 [M+H]+	0.0015	11.49	
CL 78:1	1547.175	1548.1824 [M+H]+	0.0007	11.15	
DG 20:0	400.316	401.3234 [M+H]+	0.0029	8.3	FA 23:1-O3
DG 32:0	568.505	569.5124 [M+H]+	0.0017	11.83	
DG 33:1	580.51	581.5174 [M+H]+	0.0033	9.86	
DG 34:2	592.505	593.5124 [M+H]+	0.0017	10.09	
DG 34:4	588.471	589.4784 [M+H]+	0.0044	11.59	
DG 36:0	624.568	625.5754 [M+H]+	0.0013	12.25	
DG 36:1	622.552	623.5594 [M+H]+	0.0016	11.03	
DG 36:3	618.519	619.5264 [M+H]+	0.0033	12.07	
DG 36:4	616.503	617.5104 [M+H]+	0.0037	11.85	
DG 38:3	646.55	647.5574 [M+H]+	0.0036	12.25	
DG 38:5	642.518	643.5254 [M+H]+	0.0043	11.86	
DG 40:10	660.477	661.4844 [M+H]+	0.0016	9.08	PA 33:1
DG 41:3	688.603	689.6104 [M+H]+	0.0024	11.81	
DG 42:10	688.509	689.5164 [M+H]+	0.0047	9.56	PA 35:1
DG 43:3	716.634	717.6414 [M+H]+	0.0021	12.04	
FA 10:0-O	188.141	187.1336 [M-H]-	0.0002	4.58	Hydroxydecanoic acid
FA 11:1-O2	216.135	215.1276 [M-H]-	0.0012	3.57	Undecanedioic acid
FA 12:0-O	216.173	215.1656 [M-H]-	0.0005	6.18	Hydroxylauric acid
FA 12:1	198.161	199.1684 [M+H]+	0.001	7.76	Dodecenoic acid
FA 12:1-O	214.156	215.1634 [M+H]+	0.0009	5.16	Oxododecanoic acid
FA 12:1-O iso n	214.157	213.1496 [M-H]-	0.0001	5.14	Oxododecanoic acid
FA 14:1	226.192	227.1994 [M+H]+	0.0013	6.46	Myristoleic acid
FA 16:2	252.208	253.2154 [M+H]+	0.0009	6.88	Palmitolinoleic acid
FA 18:0	284.272	283.2646 [M-H]-	0.0005	7.37	Stearic acid
FA 18:0-O	300.267	299.2596 [M-H]-	0.0006	7.66	MG O-15:1; Hydroxystearic acid
FA 18:1 iso n	282.256	281.2486 [M-H]-	0.0001	7.92	Octadecenoic acid; Oleic acid; Vaccenic acid; Elaidic acid
FA 18:1 iso1 p	282.254	283.2614 [M+H]+	0.0019	8.22	Octadecenoic acid; Oleic acid; Vaccenic acid; Elaidic acid
FA 18:1 iso2 p	282.256	283.2634 [M+H]+	0.0001	9.15	Octadecenoic acid; Oleic acid; Vaccenic acid; Elaidic acid
FA 18:1-O iso1	298.251	297.2436 [M-H]-	0.0002	7.43	Hydroxyoleic acid;Oxostearic acid
FA 18:1-O iso2	298.251	297.2436 [M-H]-	0.0002	6.89	Hydroxyoleic acid;Oxostearic acid
FA 18:1-O iso3	298.252	297.2446 [M-H]-	0.0012	7.13	Hydroxyoleic acid;Oxostearic acid
FA 18:1-O2	314.246	313.2386 [M-H]-	0.0003	6.92	MG 15:1; Octadecanedioic acid; DiHOME
FA 18:1-O2 iso	314.246	313.2386 [M-H]-	0.0003	7.74	MG 15:1; Octadecanedioic acid; DiHOME
FA 18:2	280.24	279.2326 [M-H]-	0.0002	7.73	Linoleic acid; Linoelaidic acid
FA 18:3	278.224	279.2314 [M+H]+	0.0006	5.76	Octadecatrienoic Acid; Linolenic acid
FA 18:3 iso	278.224	279.2314 [M+H]+	0.0006	6.54	Octadecatrienoic Acid; Linolenic acid
FA 20:1-O3	358.272	357.2646 [M-H]-	0.0001	7.77	Dioctanoyl-butanetriol
FA 20:4-O2 iso1 n	336.228	335.2206 [M-H]-	0.0021	4.94	MG 17:4; LTB4; PGA1; PGB1; PGC1; HpETE
FA 20:4-O2 iso1 p	336.227	337.2344 [M+H]+	0.0031	5.75	MG 17:4; LTB4; PGA1; PGB1; PGC1; HpETE
FA 20:4-O2 iso2 n	336.229	335.2216 [M-H]-	0.0011	6.73	MG 17:4; LTB4; PGA1; PGB1; PGC1; HpETE
FA 20:4-O2 iso2 p	336.227	337.2344 [M+H]+	0.0031	6.33	MG 17:4; LTB4; PGA1; PGB1; PGC1; HpETE
FA 20:4-O2 iso3 n	336.232	335.2246 [M-H]-	0.0019	7.14	MG 17:4; LTB4; PGA1; PGB1; PGC1; HpETE
FA 20:5-O	318.22	317.2126 [M-H]-	0.0005	7.38	EpETE; Oxo-ETE
LTE3	441.252	442.2594 [M+H]+	0.0028	7.42	LysoPE 14:0-O, LysoPS O-13:0
LTE4	439.235	438.2276 [M-H]-	0.0015	7.03	LysoPE 14:1-O, LysoPS O-13:1
LysoPE 16:0	453.287	452.2796 [M-H]-	0.0015	7.17	LysoPC 13:0
LysoPE 16:1	451.268	452.2754 [M+H]+	0.0019	7.52	LysoPC 13:1
LysoPE 17:0	467.303	466.2956 [M-H]-	0.0018	7.34	LysoPC 14:0
LysoPE 18:0	481.316	482.3234 [M+H]+	0.0008	8.97	LysoPE 18:0
LysoPE 18:1 iso n	479.303	478.2956 [M-H]-	0.0018	7.27	LysoPC 15:1
LysoPE 18:1 iso p	479.3	480.3074 [M+H]+	0.0012	8.43	LysoPC 15:1
LysoPE 18:2 iso p	477.285	478.2924 [M+H]+	0.0005	8.23	LysoPC 15:2
LysoPE 18:2 iso1 n	477.286	476.2786 [M-H]-	0.0005	7.14	LysoPC 15:2
LysoPE 18:2 iso2 n	477.29	476.2826 [M-H]-	0.0045	7.29	LysoPC 15:2
LysoPE 20:4	501.282	502.2894 [M+H]+	0.0035	8.44	
LysoPE 24:0	565.411	566.4184 [M+H]+	0.0003	9.28	LysoPC 21:0; LysoPE 24:0
LysoPG 16:0	484.279	485.2864 [M+H]+	0.0011	7.8	
LysoPG 18:0	512.313	511.3056 [M-H]-	0.0016	7.27	
LysoPG 18:1	510.293	511.3004 [M+H]+	0.0028	8.05	
LysoPG 19:1	524.311	525.3184 [M+H]+	0.0004	9.57	
LysoPG 20:5	530.266	531.2734 [M+H]+	0.0015	8.43	
LysoPS 17:1	509.275	508.2676 [M-H]-	0.0004	6.69	
LysoPS 19:0	539.322	538.3146 [M-H]-	0.0003	6.72	
LysoPS 19:1	537.308	536.3006 [M-H]-	0.0013	7.1	
LysoPS 20:3	547.29	546.2826 [M-H]-	0.001	7.28	
LysoPS 24:1 iso n	607.386	606.3786 [M-H]-	0.0011	7.33	PE 25:0 cyclo
LysoPS 24:1 iso p	606.381	607.3884 [M+H]+	0.0042	7.44	TG 31:5-O3
MG 14:1 iso n	300.23	299.2226 [M-H]-	0.0001	6.89	FA 17:1-O2
MG 14:1 iso p	300.228	301.2354 [M+H]+	0.0021	7.71	FA 17:1-O2
MG 15:0	316.261	317.2684 [M+H]+	0.0004	8.72	FA 18:0-O2
MG 15:1	314.245	315.2524 [M+H]+	0.0007	6.63	FA 18:0-O2
MG 16:1 iso n	328.261	327.2536 [M-H]-	0.0004	7.81	FA 19:1-O2

MG 16:1 iso p	328.259	329.2664 [M+H]+	0.0024	8.52	FA 19:1-O2
MG 17:0	344.292	345.2994 [M+H]+	0.0007	9.29	FA 20:0-O2
MG 18:1	356.291	357.2984 [M+H]+	0.0017	7.51	FA 21:1-O2
MG 18:1 iso	356.292	357.2994 [M+H]+	0.0007	9.13	FA 21:1-O2
MG 18:2	354.276	353.2686 [M-H]-	0.001	7.39	FA 21:2-O2
MG 19:0	372.322	373.3294 [M+H]+	0.002	9.77	FA 22:0-O2; MG 19:0
MG 20:0	386.339	387.3464 [M+H]+	0.0006	10	FA 23:0-O2; MG 20:0
MG 22:0	414.37	415.3774 [M+H]+	0.0009	10.44	FA 25:0-O2
NAE 20:4-O	363.276	364.2834 [M+H]+	0.0013	6.05	Hydroxyanandamide; N-acylethanolamine 20:4-O
PA 30:1	618.429	619.4364 [M+H]+	0.0006	8.09	DG 37:10
PA 31:1	632.442	633.4494 [M+H]+	0.0003	8.42	DG 38:10
PA 33:2	644.479	645.4864 [M+H]+	0.0009	12.43	Rhamnosyl-C30
PA 34:1	674.487	675.4944 [M+H]+	0.004	11.08	DG 41:10
PA 34:3	670.462	671.4694 [M+H]+	0.0046	9.97	
PA 34:6	664.414	665.4214 [M+H]+	0.0036	7.64	
PA 35:0	690.521	691.5284 [M+H]+	0.001	9	DG 42:9
PA 35:6	678.43	679.4374 [M+H]+	0.0039	8.03	
PA 36:3	698.493	699.5004 [M+H]+	0.002	10.36	DG 43:12
PA 37:3	712.503	713.5104 [M+H]+	0.0013	9.01	DG 44:12
PA 38:5	722.5	723.5074 [M+H]+	0.0031	10.16	MGDG 32:4
PA 38:5 iso	722.492	723.4994 [M+H]+	0.0033	9.6	
PA 39:0	746.584	747.5914 [M+H]+	0.0014	9.55	DG 46:9
PA 39:3	740.538	741.5454 [M+H]+	0.0025	10.46	DG 46:12
PA 39:3 iso	740.539	741.5464 [M+H]+	0.001	9.96	DG 46:12
PA 39:5	736.506	737.5134 [M+H]+	0.0017	9.92	
PA 40:6	748.507	749.5144 [M+H]+	0.0027	9.5	
PA 41:3	768.569	769.5764 [M+H]+	0.0003	10.97	DG 48:12
PA 41:5	764.538	765.5454 [M+H]+	0.0024	10.23	
PA 42:4	780.564	781.5714 [M+H]+	0.0029	11.4	
PA 43:0	802.645	803.6524 [M+H]+	0.0002	10.01	DG 50:9
PA 43:4	794.583	795.5904 [M+H]+	0.0004	11.01	DG 50:13; Rhamnosyl-Rha-C30
PA 43:6	790.547	791.5544 [M+H]+	0.0043	10.3	
PA 44:10	796.505	797.5124 [M+H]+	0.0007	10.32	
PA O-32:0	634.493	635.5004 [M+H]+	0.0007	12.07	PA O-32:1-O
PA O-34:0	662.523	663.5304 [M+H]+	0.002	12.25	PA 34:0
PA O-35:1	674.524	675.5314 [M+H]+	0.001	8.38	
PA P-28:1	576.412	577.4194 [M+H]+	0.0035	8.74	
PA P-36:4	680.475	681.4824 [M+H]+	0.0031	11.39	PA O-36:5
PA P-38:4	708.513	709.5204 [M+H]+	0.0036	10.66	PA O-38:5
PE 18:0	495.296	494.2886 [M-H]-	0.0001	6.88	LysoPE 18:1-O; LysoPC 15:1-O
PE 28:0	635.451	636.4584 [M+H]+	0.0016	10.32	
PE 28:1	633.436	634.4434 [M+H]+	0.001	10.08	
PE 30:0	663.483	664.4904 [M+H]+	0.0009	10.74	
PE 30:2	659.451	660.4584 [M+H]+	0.0016	10.11	
PE 31:1	675.482	676.4894 [M+H]+	0.0019	10.64	PC 28:1
PE 32:0	691.514	692.5214 [M+H]+	0.0012	11.18	PC 29:0
PE 32:1	689.498	690.5054 [M+H]+	0.0016	10.79	PC 29:1
PE 32:2	687.483	688.4904 [M+H]+	0.0009	10.43	PC 29:2
PE 34:2	715.514	716.5214 [M+H]+	0.0012	10.91	PC 31:2
PE 34:3	713.498	714.5054 [M+H]+	0.0016	10.69	PC 31:3
PE 34:5	709.464	710.4714 [M+H]+	0.0043	10.42	PC 31:5
PE 36:5	737.497	738.5044 [M+H]+	0.0026	10.97	PC 33:5
PE 38:1	773.591	774.5984 [M+H]+	0.0025	11.37	PC 35:1
PE 38:5	765.527	766.5344 [M+H]+	0.0039	8.27	PC 35:5
PE 40:4	795.581	796.5884 [M+H]+	0.0032	11.4	PC 37:4
PE 42:10	811.512	812.5194 [M+H]+	0.0032	7.9	PC 39:10
PE 44:10	839.542	840.5494 [M+H]+	0.0045	2.99	PC 41:10
PE O-28:0	621.474	622.4814 [M+H]+	0.0007	9.81	LysoPC 25:0
PE P-31:1	659.489	660.4964 [M+H]+	0.0001	11.85	PE 31:2; PC 28:2
PE P-36:4	723.523	724.5304 [M+H]+	0.0027	11.41	PE O-36:5
PE P-37:1	743.583	744.5904 [M+H]+	0.0002	12.19	PC O-34:2; PE O-37:2
PG 30:0	694.476	695.4834 [M+H]+	0.0025	10.12	
PG 32:0	722.509	723.5164 [M+H]+	0.0008	10.46	
PG 32:1	720.494	721.5014 [M+H]+	0.0001	10.16	
PG 32:2	718.477	719.4844 [M+H]+	0.0015	9.92	
PG 34:1	748.525	749.5324 [M+H]+	0.0004	10.52	
PG 34:2	746.508	747.5154 [M+H]+	0.0018	10.23	
PG 36:2	774.544	775.5514 [M+H]+	0.0029	8.31	
PG 36:6	766.482	767.4894 [M+H]+	0.0035	7.94	
PS 29:0	693.456	694.4634 [M+H]+	0.0021	8.16	
PS 33:0	749.522	750.5294 [M+H]+	0.0013	10.09	
PS 33:1	747.504	748.5114 [M+H]+	0.001	9.63	
PS 35:0	777.551	778.5584 [M+H]+	0.001	10.43	
PS 35:1	775.537	776.5444 [M+H]+	0.0007	10.62	
PS 37:1	801.551	802.5584 [M+H]+	0.001	10.65	
PS 37:4	797.518	798.5254 [M+H]+	0.0027	10.64	
PS 37:5	795.501	796.5084 [M+H]+	0.004	10.32	
PS 40:4	839.563	840.5704 [M+H]+	0.0046	7.83	
PS 40:5	837.556	838.5634 [M+H]+	0.004	3.37	
PS 41:5	851.563	852.5704 [M+H]+	0.0046	3.53	
PS 41:6	849.551	850.5584 [M+H]+	0.001	7.85	
PS 43:6	877.584	878.5914 [M+H]+	0.0007	7.45	
PS 43:7	874.562	875.5694 [M+H]+	0.0049	3.3	
ST 21:3-O2	314.223	313.2156 [M-H]-	0.0016	7.49	Progesterone; FA 21:6

ST 21:4;O2	312.209	311.2016 [M-H]-	0.0001	7.08	Pregnadienedione; Delta1-progesterone
TG 30:0	554.457	553.4496 [M-H]-	0.0024	7.77	FA 33:2-O4
TG 39:0	680.594	681.6014 [M+H]+	0.0015	12.03	
TG 57:10	912.722	913.7294 [M+H]+	0.0013	7.84	
TG 66:15	1028.783	1029.7904 [M+H]+	0.0003	9	

Supplementary Table 10: Detailed list of the LC/MS lipid mass assignments.

Abbreviations: fatty acylcarnitine (CAR); cardiolipin (CL); diacylglycerol (DG); fatty acid (FA); oxidized fatty acid (FA-O); leukotriene (LTE); monoacylated glycerophospholipid (Lyso); monoacylglycerol (MG); N-acylethanolamine (NAE); phosphatidic acid (PA); phosphatidylcholine (PC); phosphatidylethanolamine (PE); phosphatidylglycerol (PG); phosphatidylserine (PS); steroid (ST); triacylglycerol (TG).

	LPB	PB	B
One-phase association			
Best-fit values			
Y0	= 0.000	= 0.000	= 0.000
Plateau	87.79	84.75	69.77
K	0.1913	0.1198	0.1062
Tau	5.227	8.349	9.414
Half-time	3.623	5.787	6.526
Span	= 87.79	= 84.75	= 69.77
95% CI (profile likelihood)			
Plateau	77.97 to 98.93	67.14 to 115.0	60.93 to 82.00
K	0.1247 to 0.3113	0.05349 to 0.2852	0.06921 to 0.1581
Tau	3.212 to 8.017	3.506 to 18.70	6.324 to 14.45
Half-time	2.226 to 5.557	2.430 to 12.96	4.383 to 10.02
Goodness of Fit			
Degrees of Freedom	7	7	7
R squared	0.9292	0.8353	0.9545
Sum of Squares	354.9	790.9	175.8
Sy.x	7.120	10.63	5.012
Constraints			
Y0	Y0 = 0	Y0 = 0	Y0 = 0
K	K > 0	K > 0	K > 0
Number of points			
# of X values	9	9	9
# Y values analyzed	9	9	9

Supplementary Table 11: Nonlinear analysis of curves shown in Fig. S6b.

SUPPLEMENTARY REFERENCES

1. Pavlova, O., Peterson, J.H., Ieva, R. & Bernstein, H.D. Mechanistic link between β barrel assembly and the initation of autotransporter secretion. *Proc Natl Acad Sci USA* **110**, E938-E947 (2013).

2. Cardona, S. T. & Valvano, M. A. An expression vector containing a rhamnose-inducible promoter provides tightly regulated gene expression in Burkholderia cenocepacia. *Plasmid* **54**, 219-228 (2005).

3. Fazli, M., Harrison, J.J., Gambino, M., Givskov, M. & Tolker-Nielsen, T. In-Frame and unmarked gene deletions in *Burkholderia cenocepacia* via an allelic exchange system compatible with Gateway technology." *Appl Environ Microbiol* **81**, 3623-3630 (2015).

4.Doyle, M. T. & Bernstein, H. D. Bacterial outer membrane proteins assemble via asymmetric interactions with the BamA β-barrel. *Nat Commun* **10**, 3358 (2019).
