# Supplementary material for: The patatin-like protein PlpD forms structurally dynamic homodimers in the Pseudomonas aeruginosa outer membrane
Source: Nat Commun. 2024 May 23;15:4389. doi: 10.1038/s41467-024-48756-6 (PMC11116518; doi:10.1038/s41467-024-48756-6)
Supplement: Supplementary file 4 — Source Data [file 41467_2024_48756_MOESM4_ESM.zip › Source Data.pdf]

Figure 1b (top)

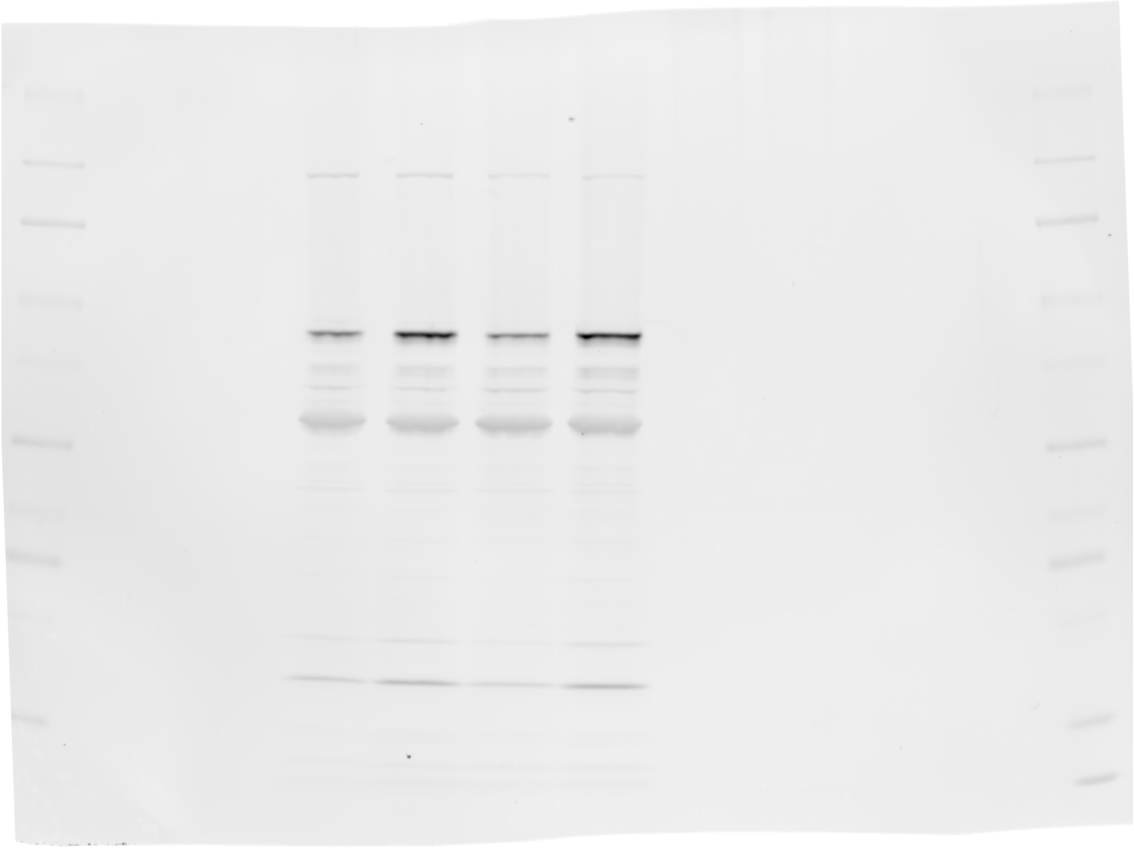

Figure 1b (bottom)

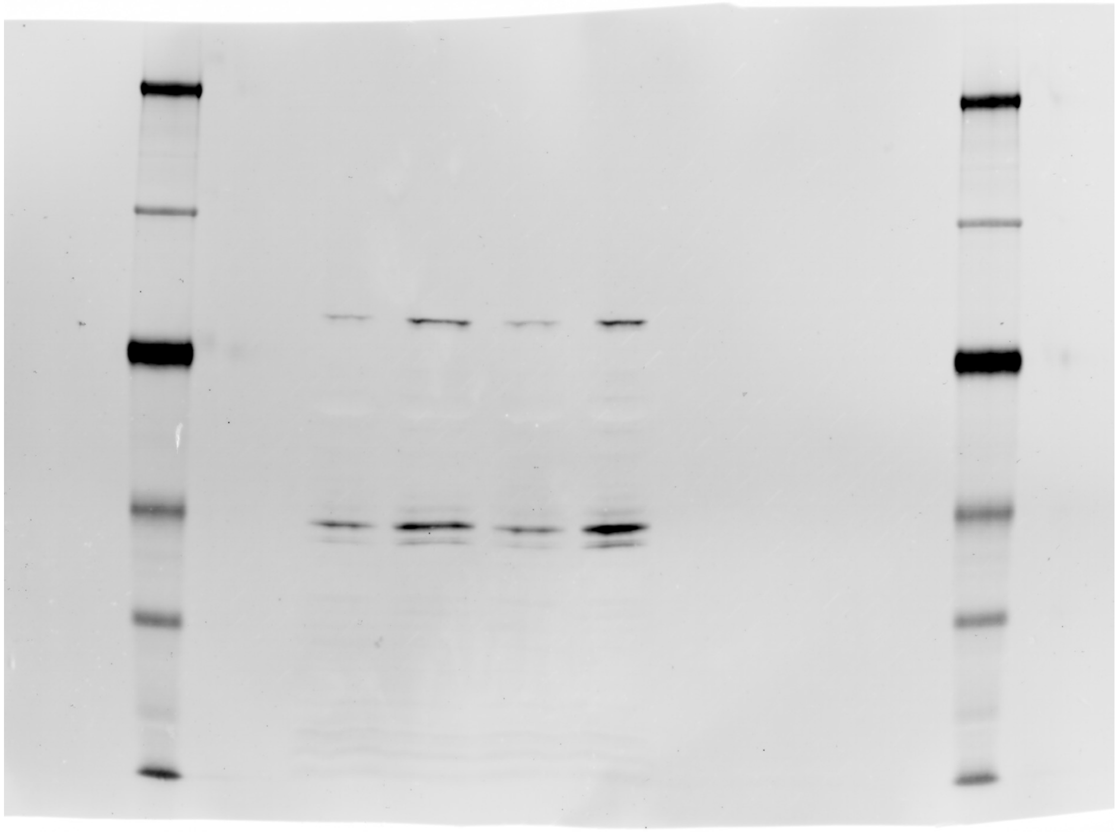

Figure 1c

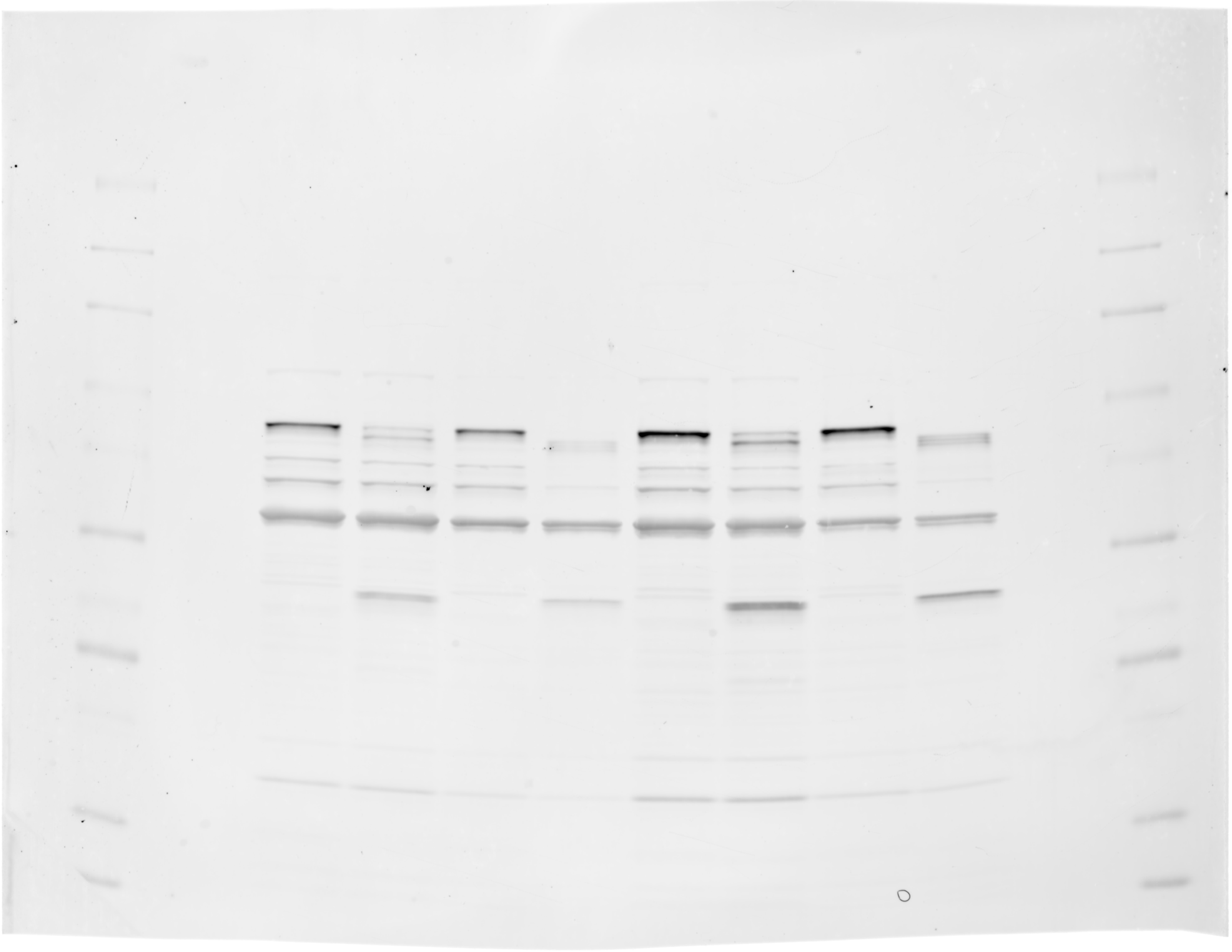

Figure 1d

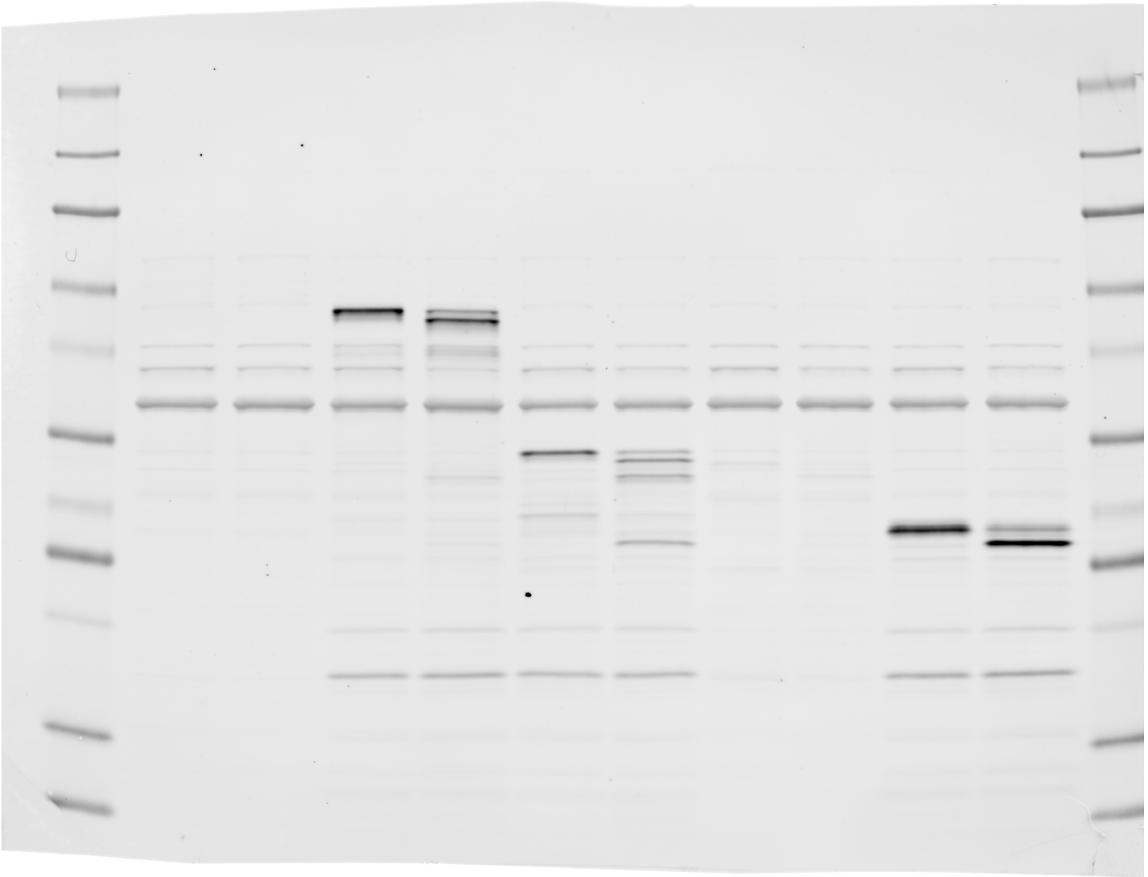

Figure 3b (top)

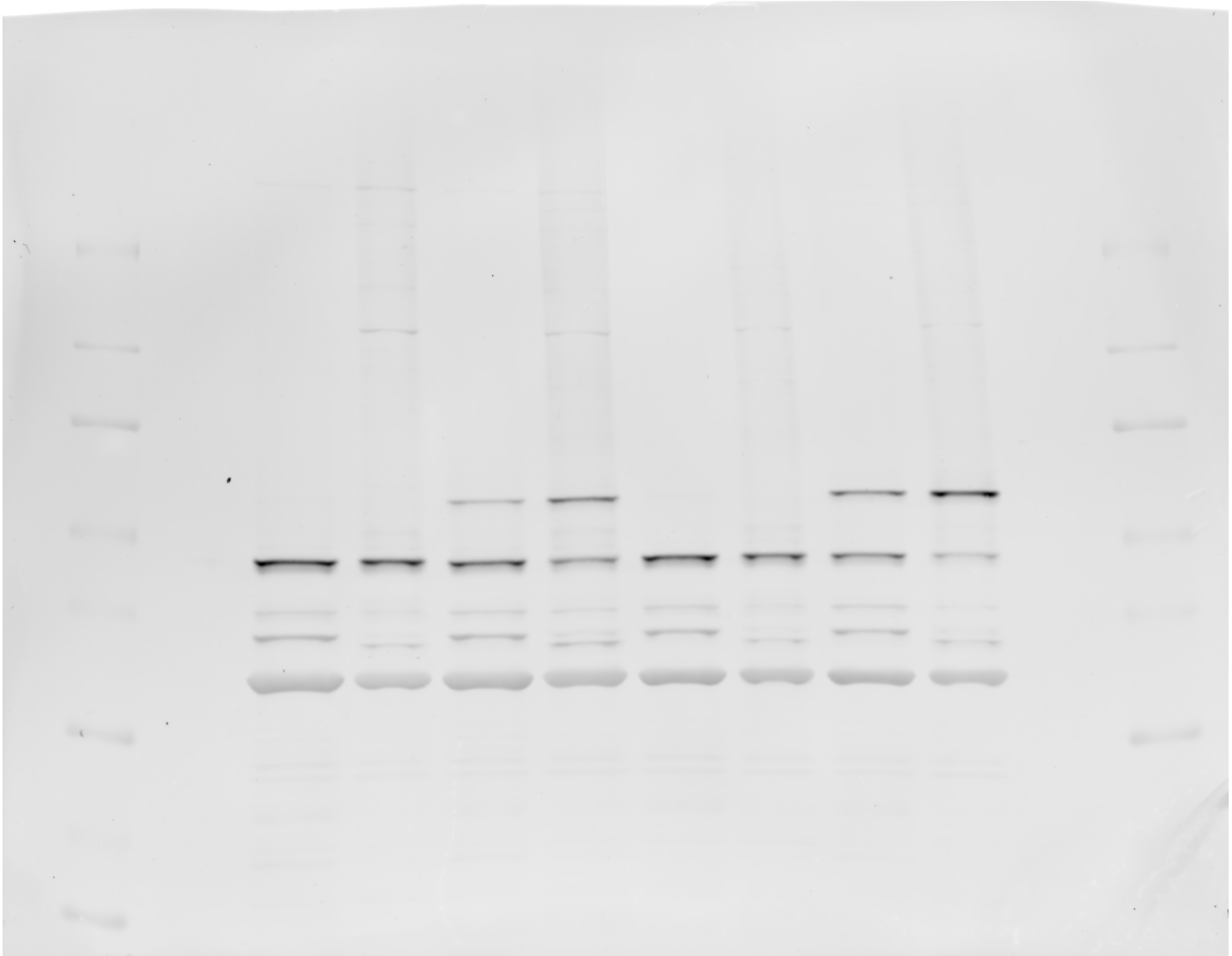

Figure 3b (bottom)

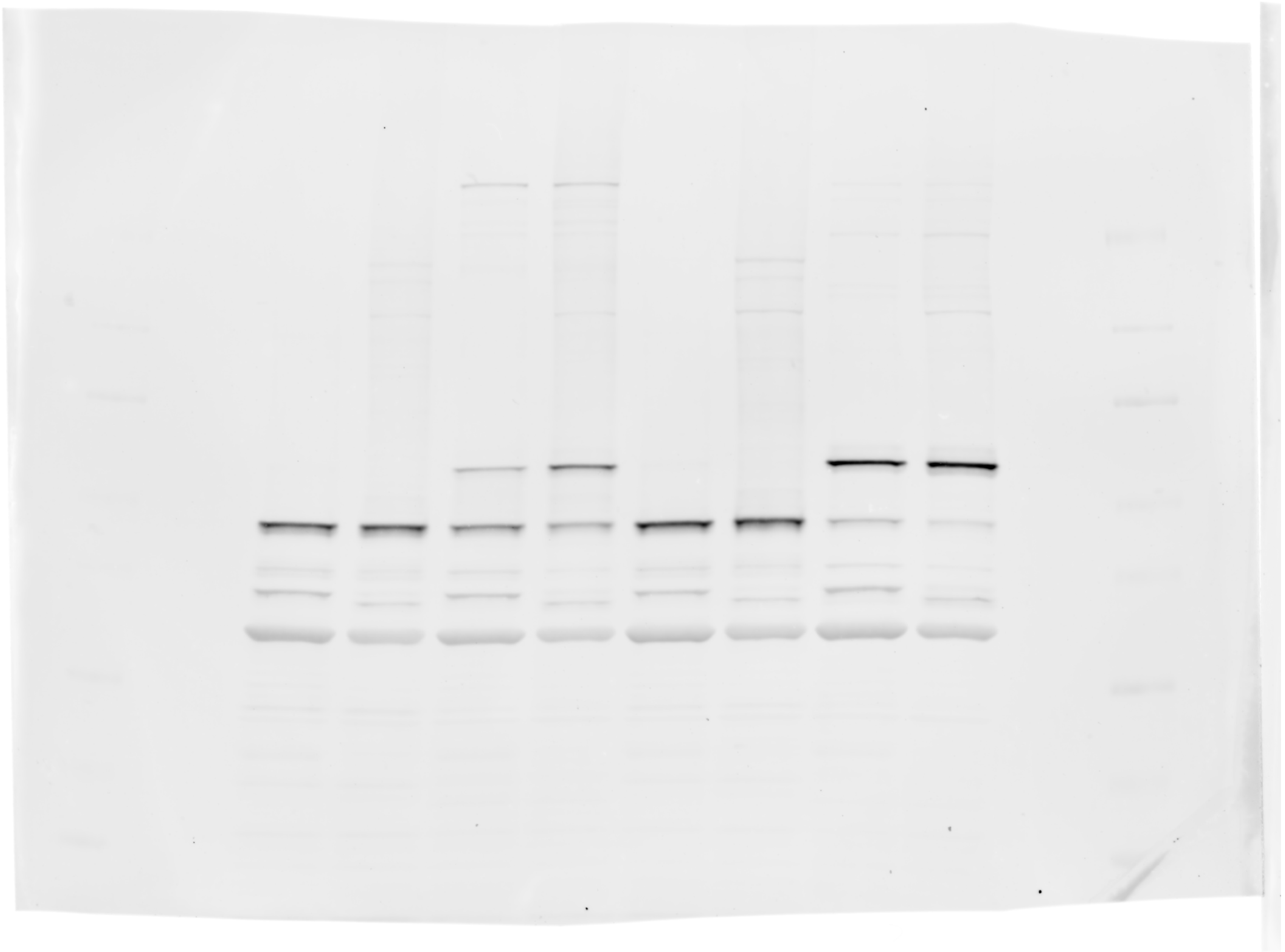

Figure 4

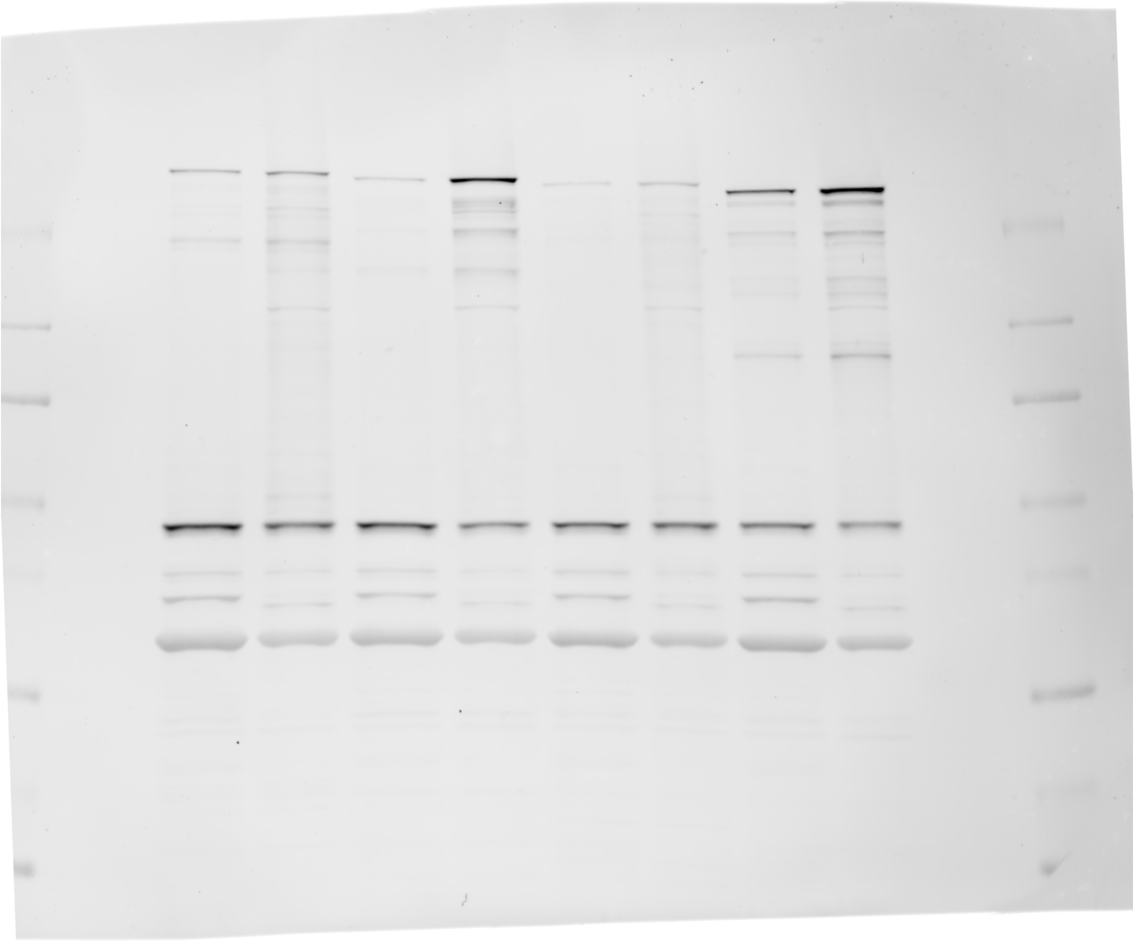

Fig. 5b left

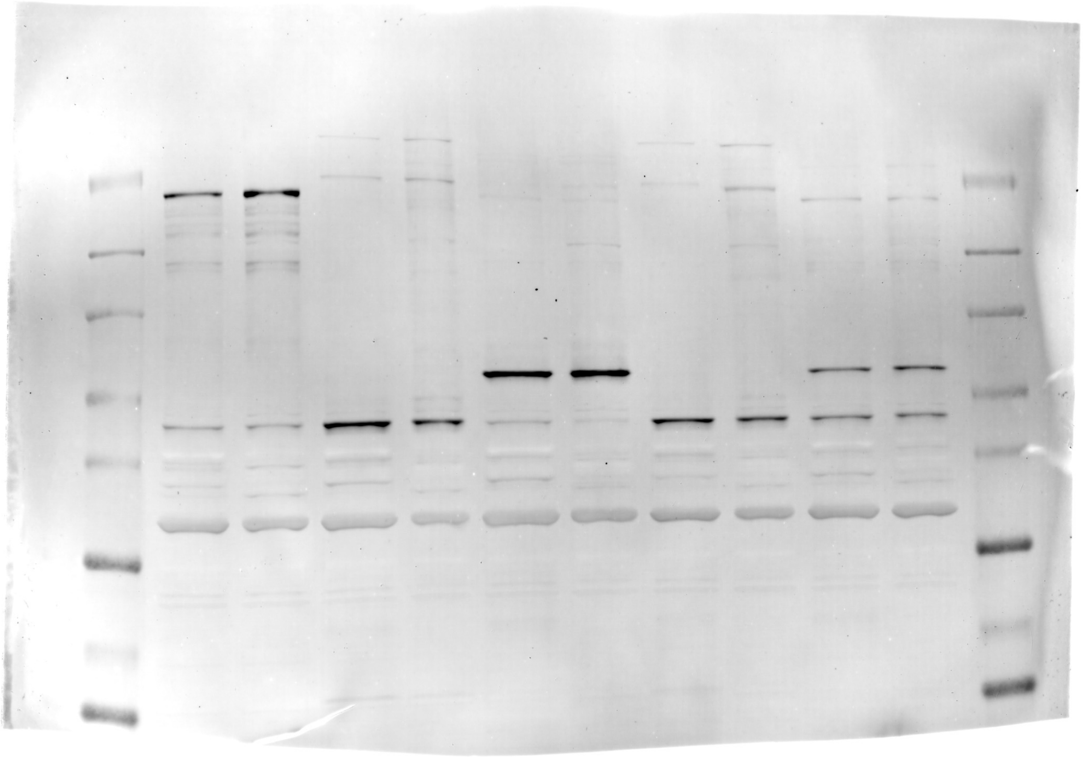

Fig. 5b right

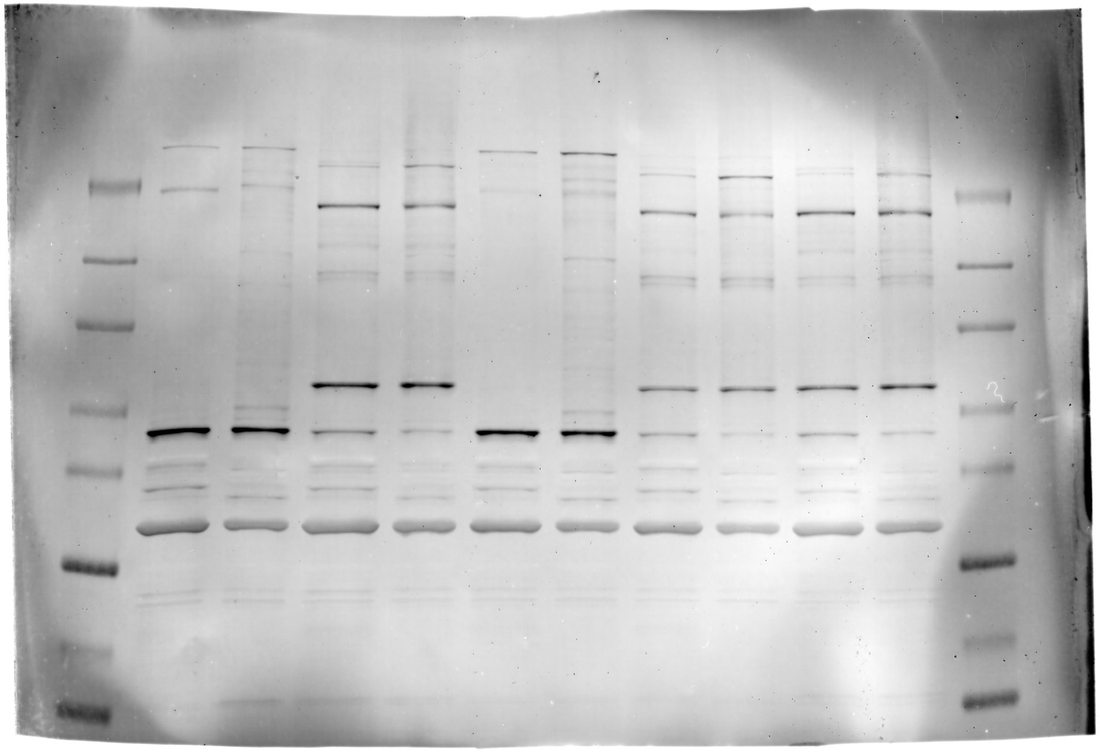

Fig. 5b bottom

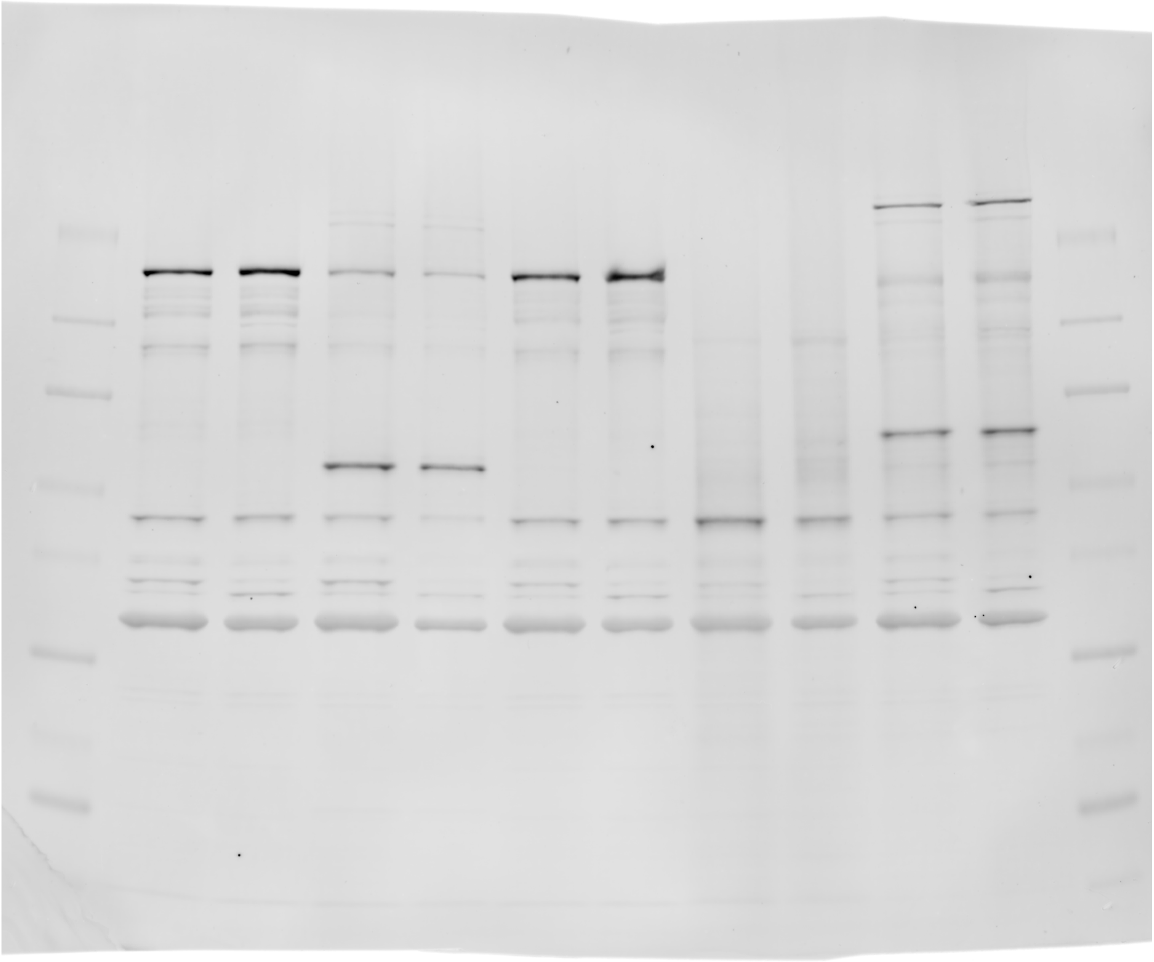

Fig. 5c

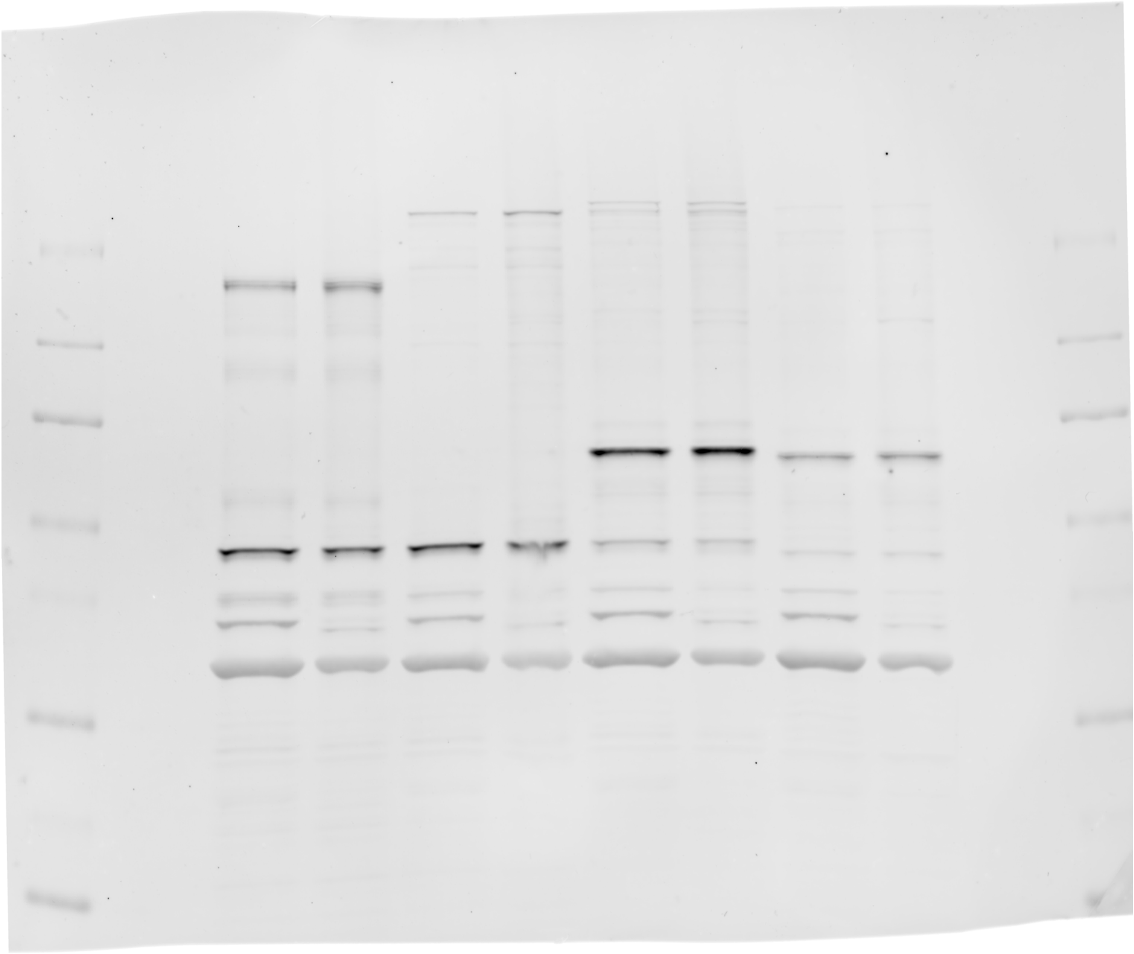

Fig. S1 (left)

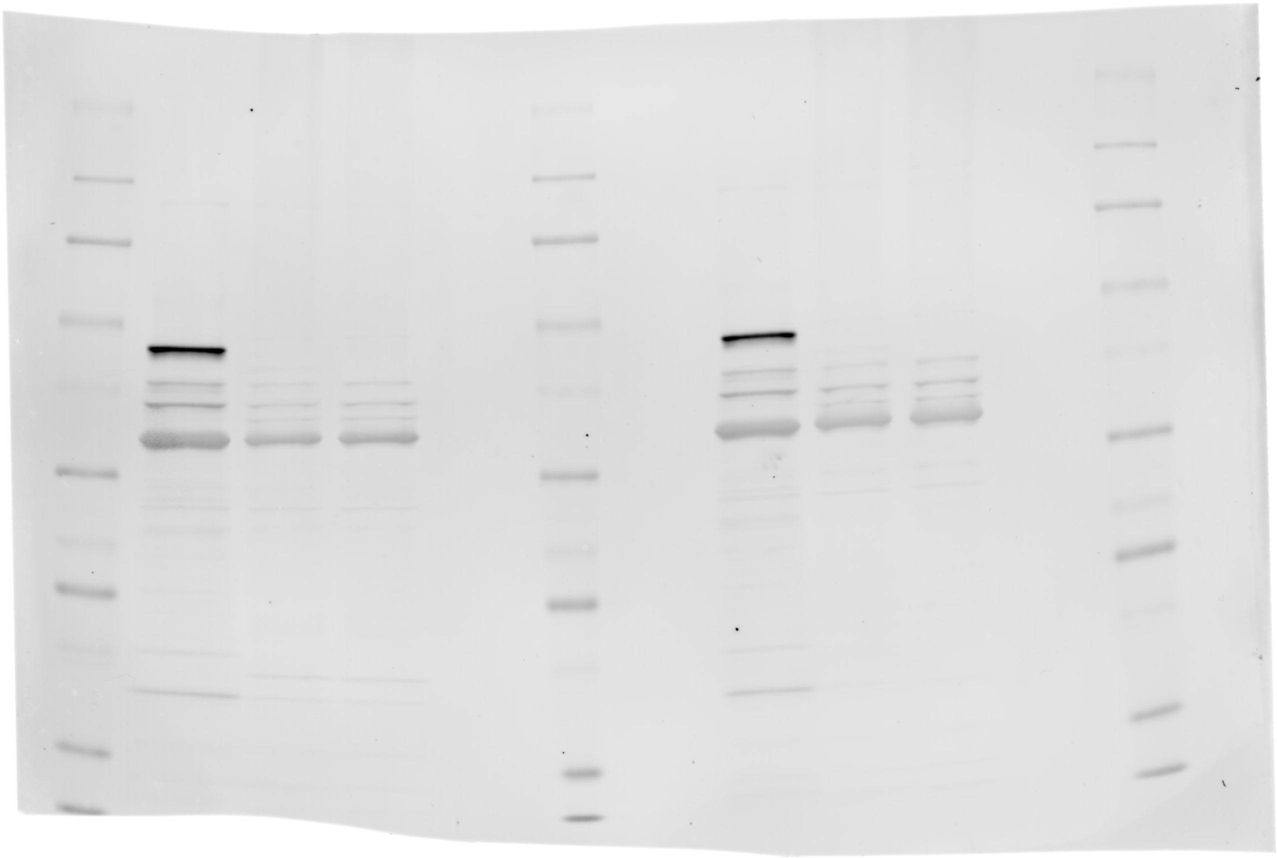

Fig. S1 (right)

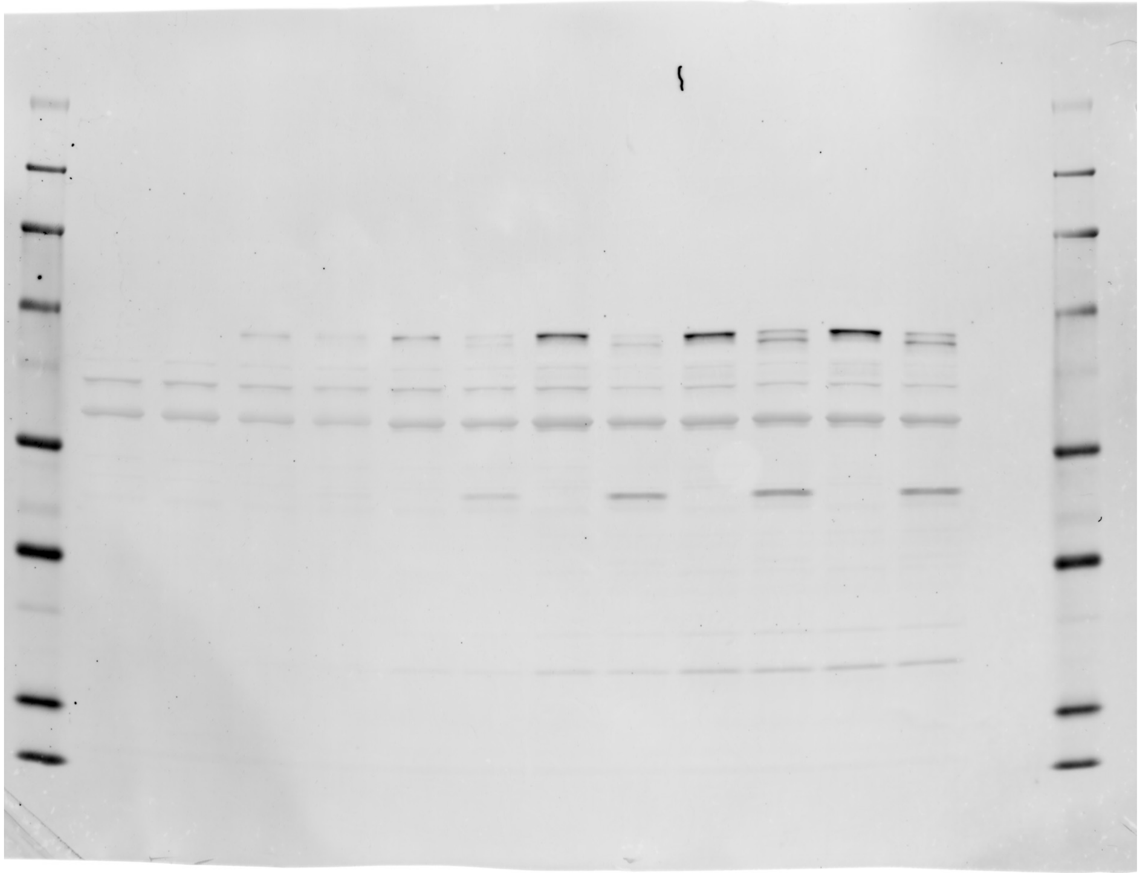

Fig. S2

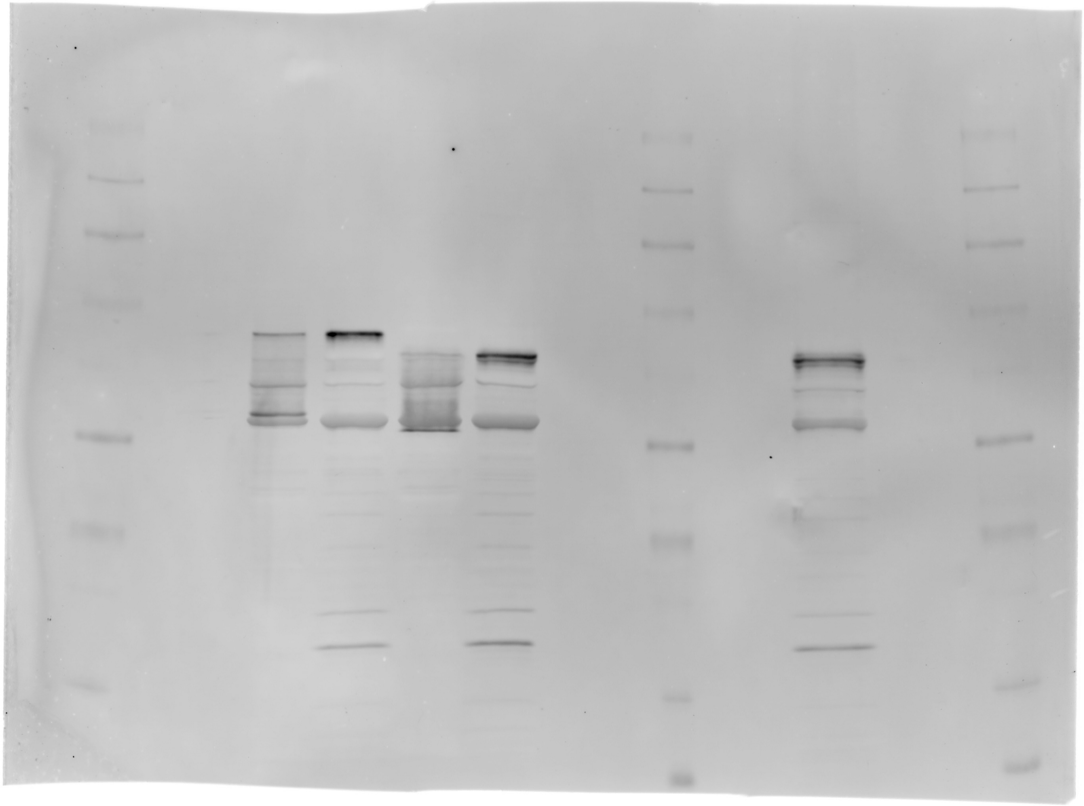

Fig. S3 top

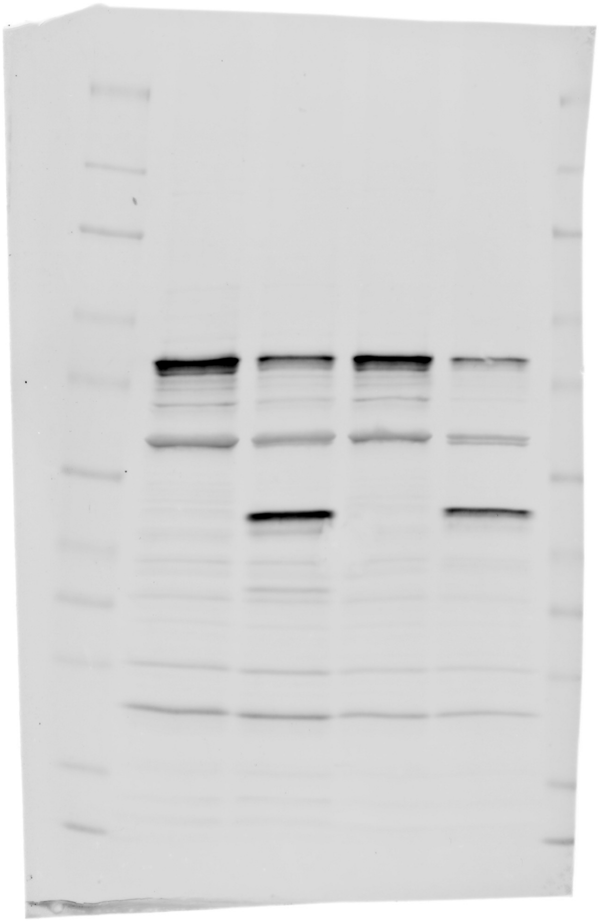

Fig. S3 bottom

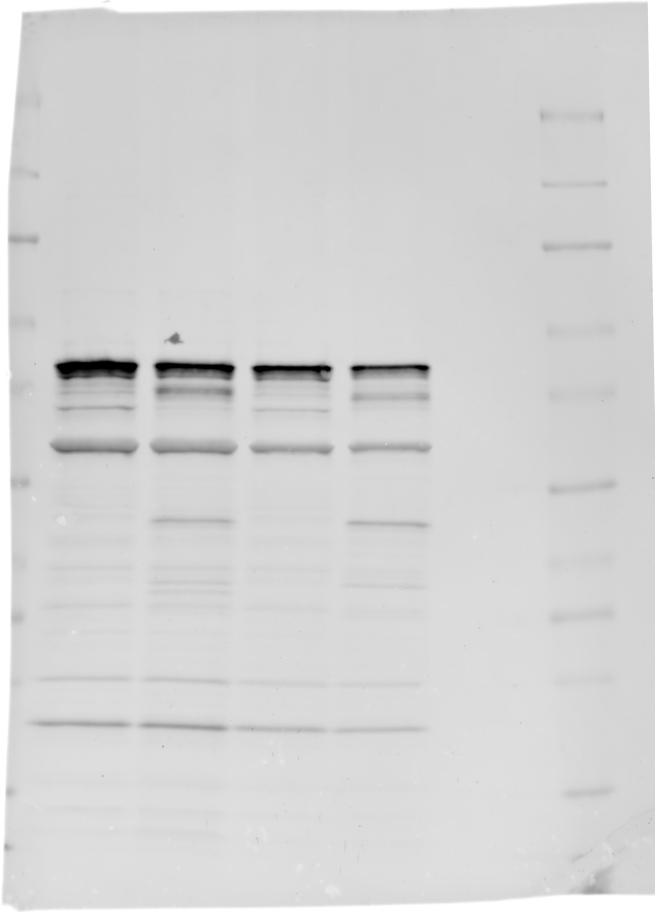

Fig. S4

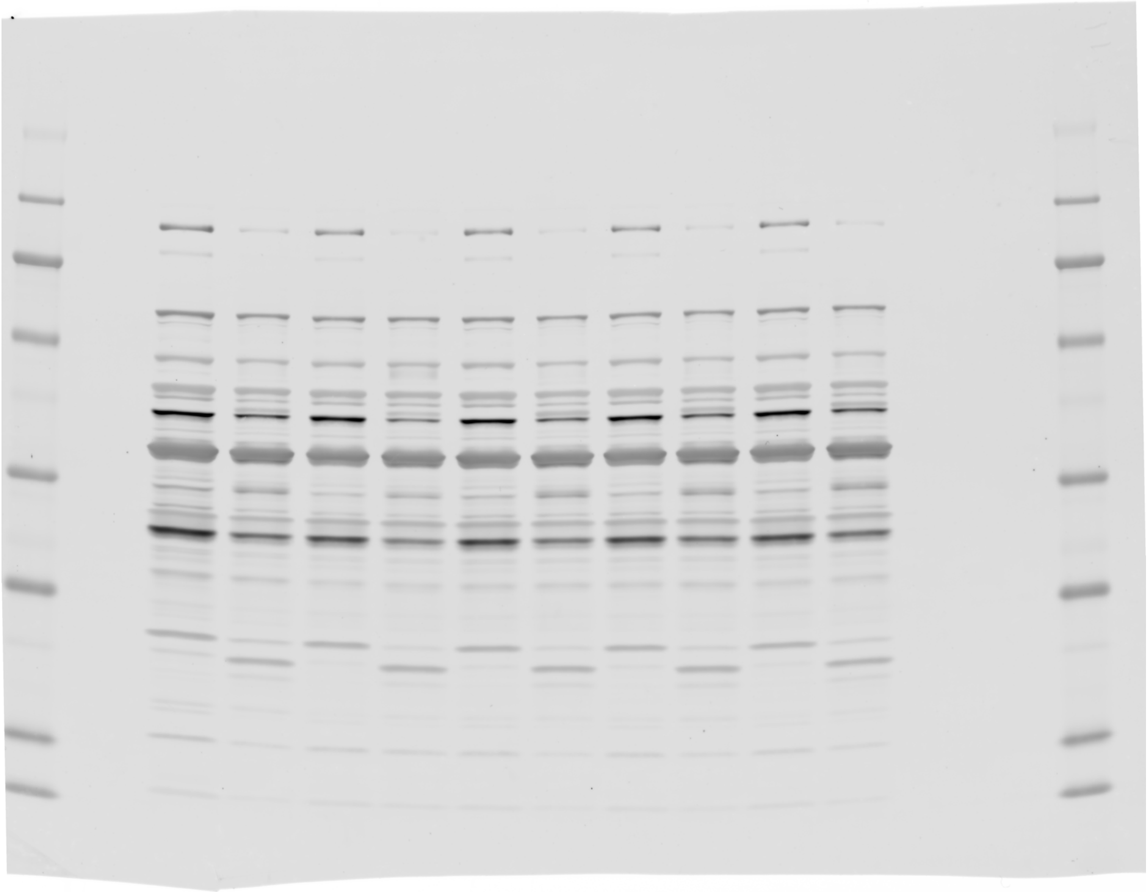

Fig. S5a

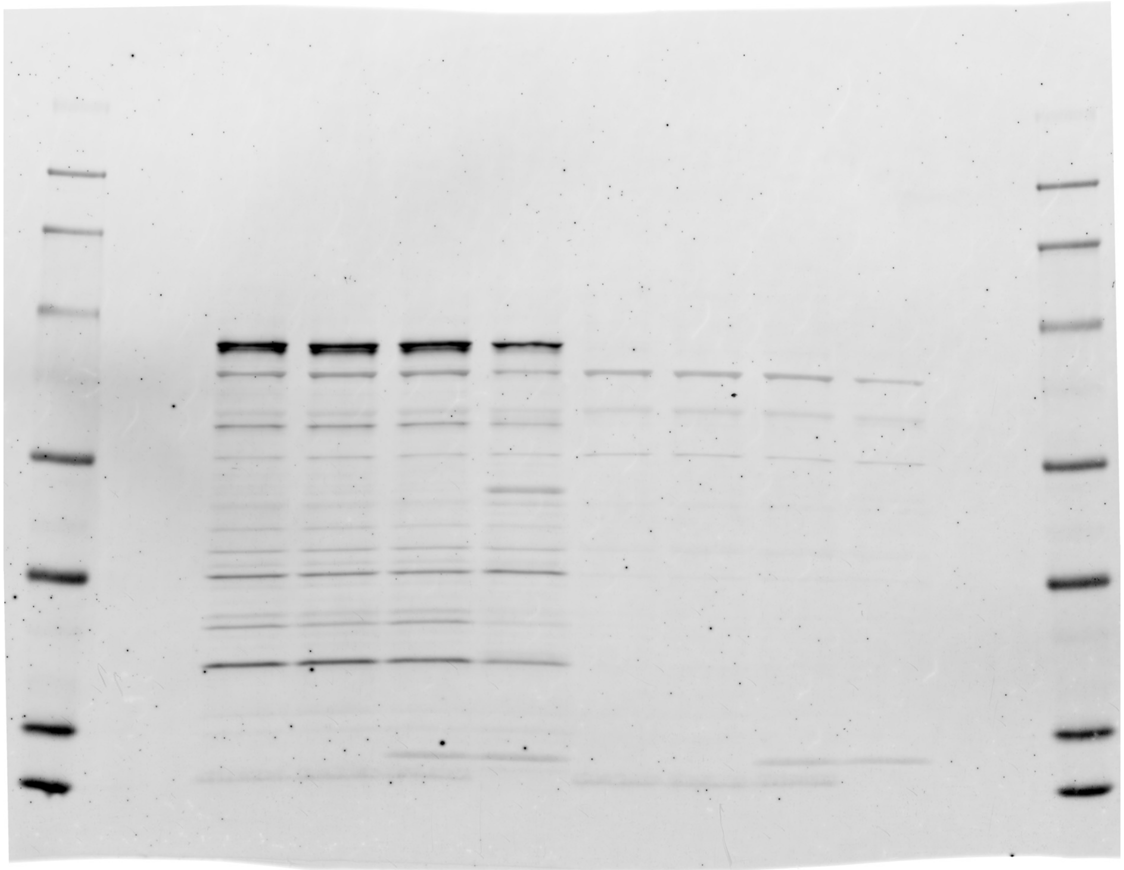

Fig. S5b

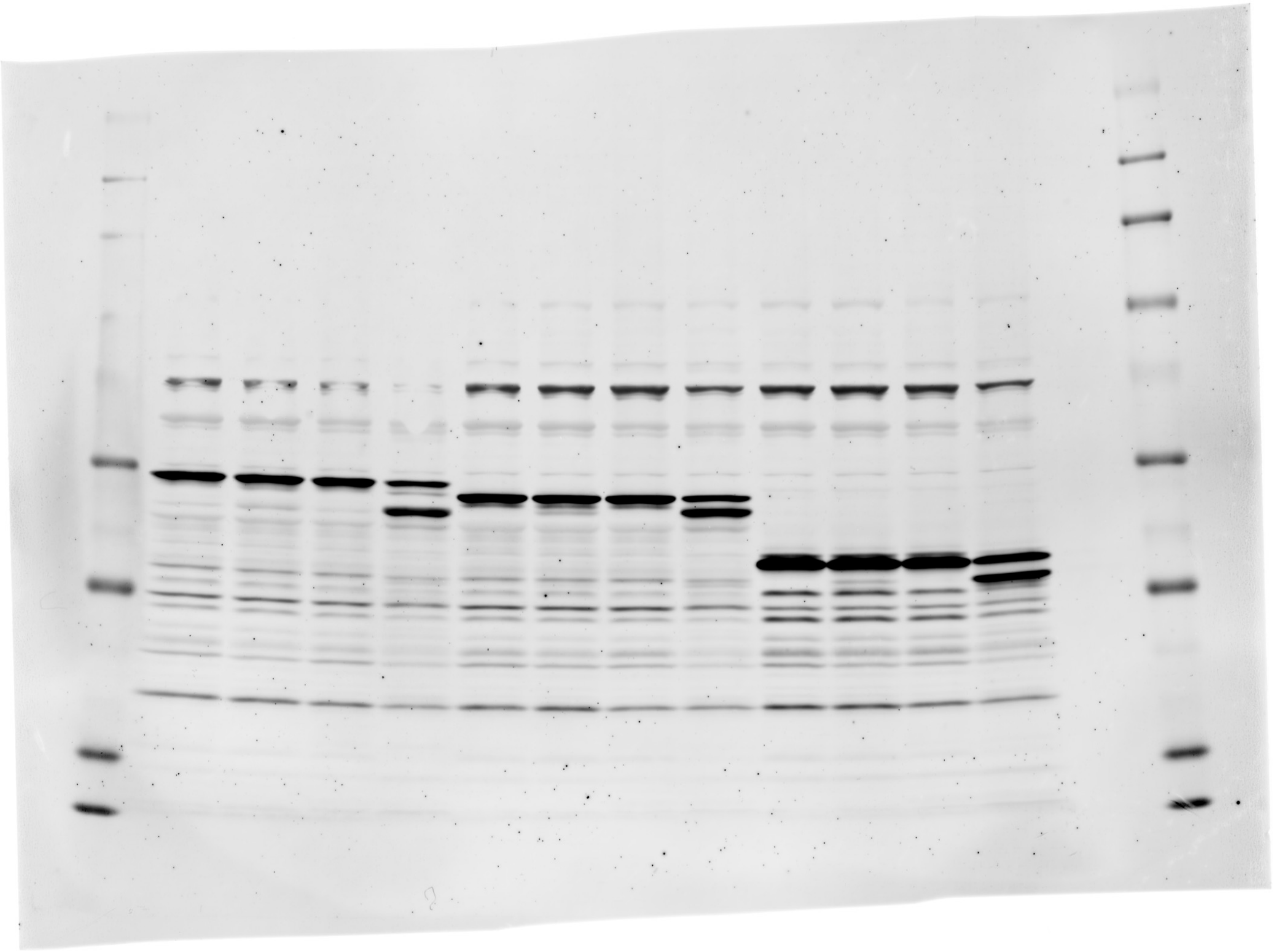

Fig. S5c

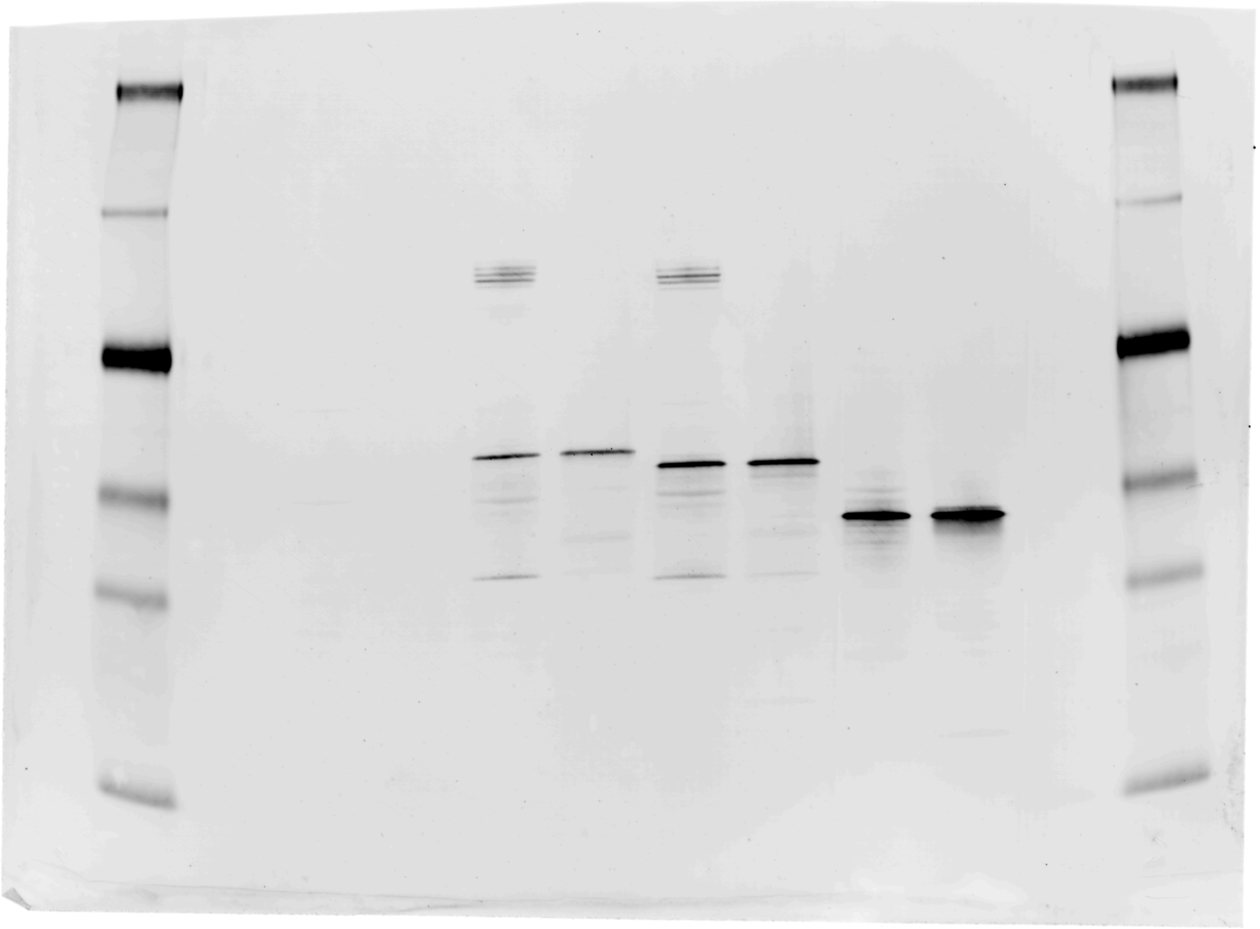

Fig. S6a (top)

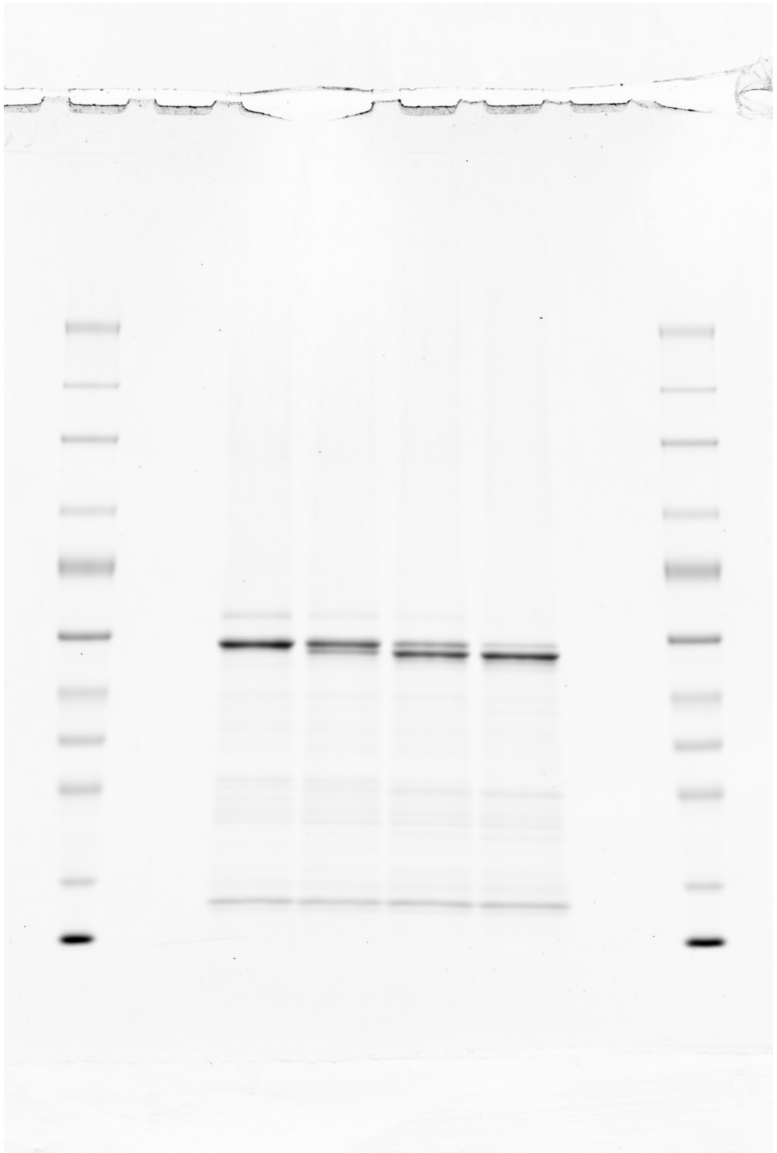

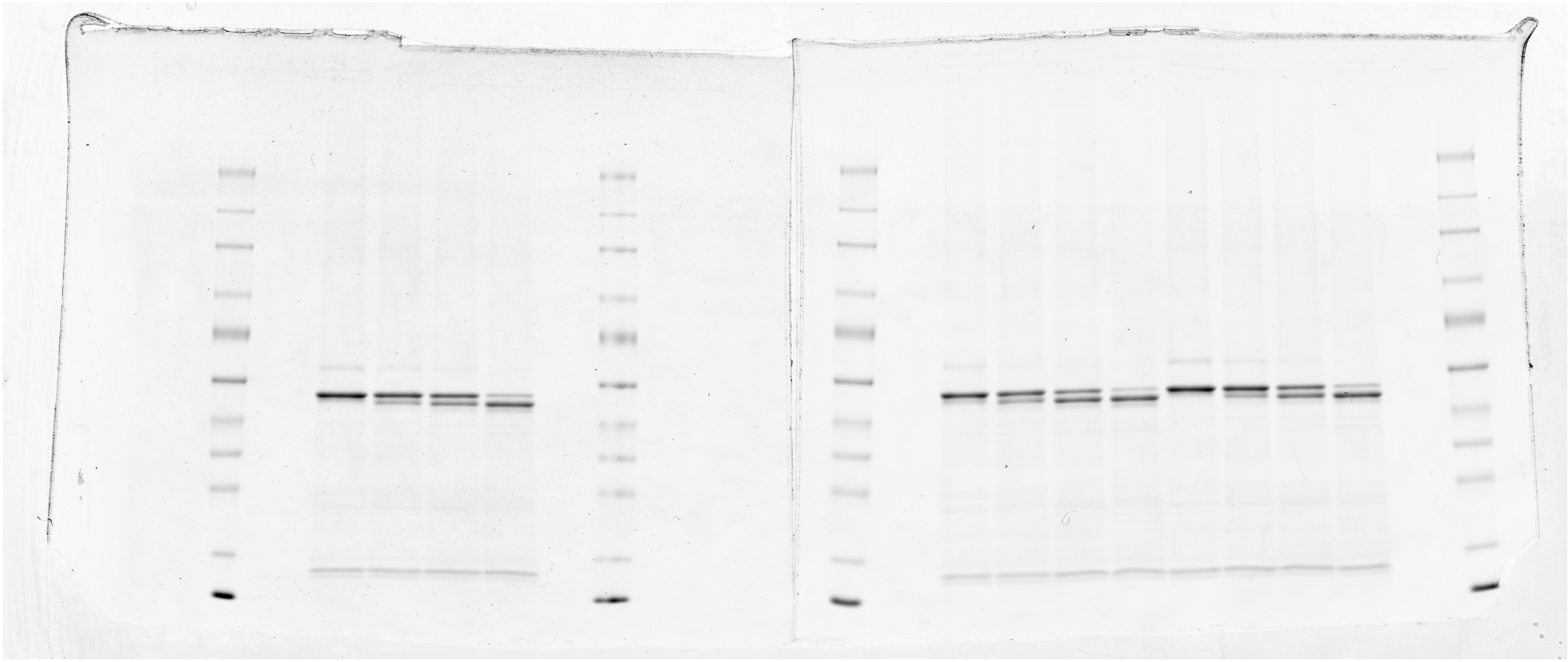

Fig. S6a (middle)

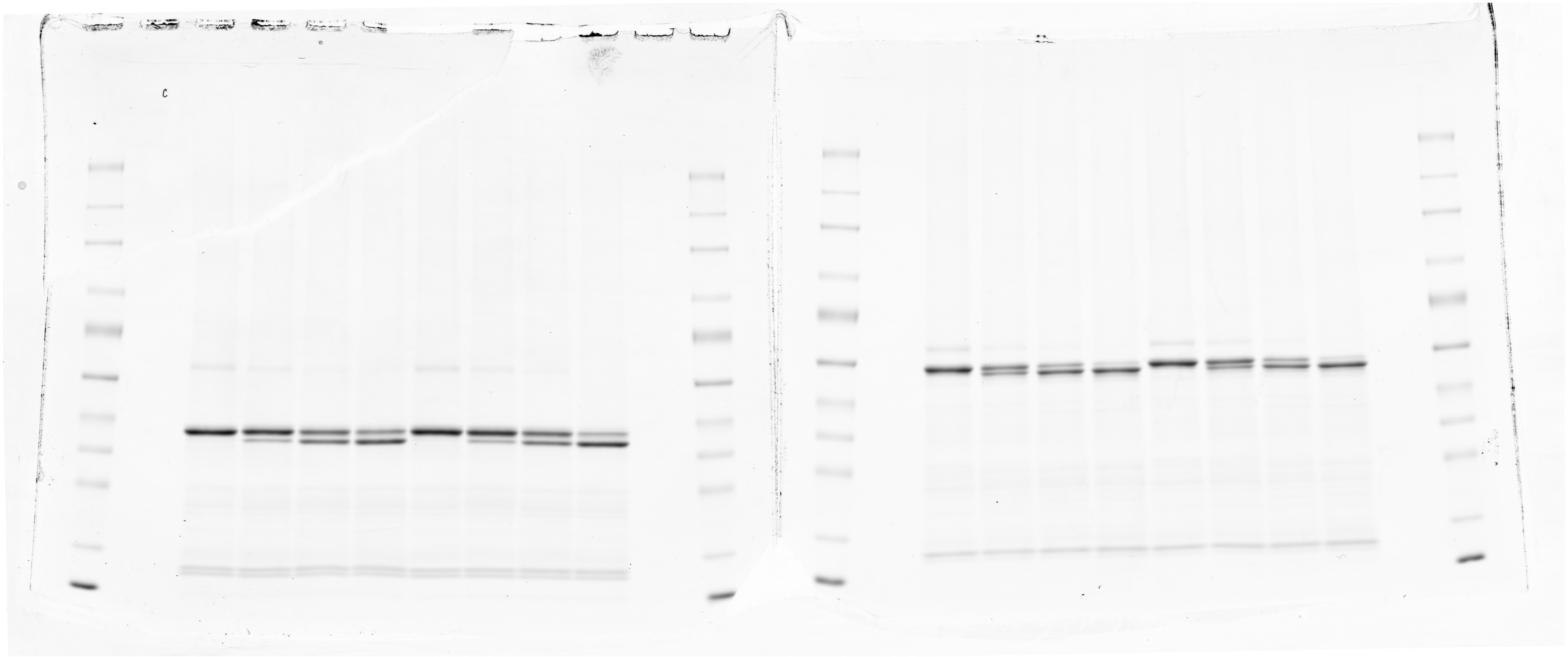

Fig. S6a (bottom)

Fig. S6c

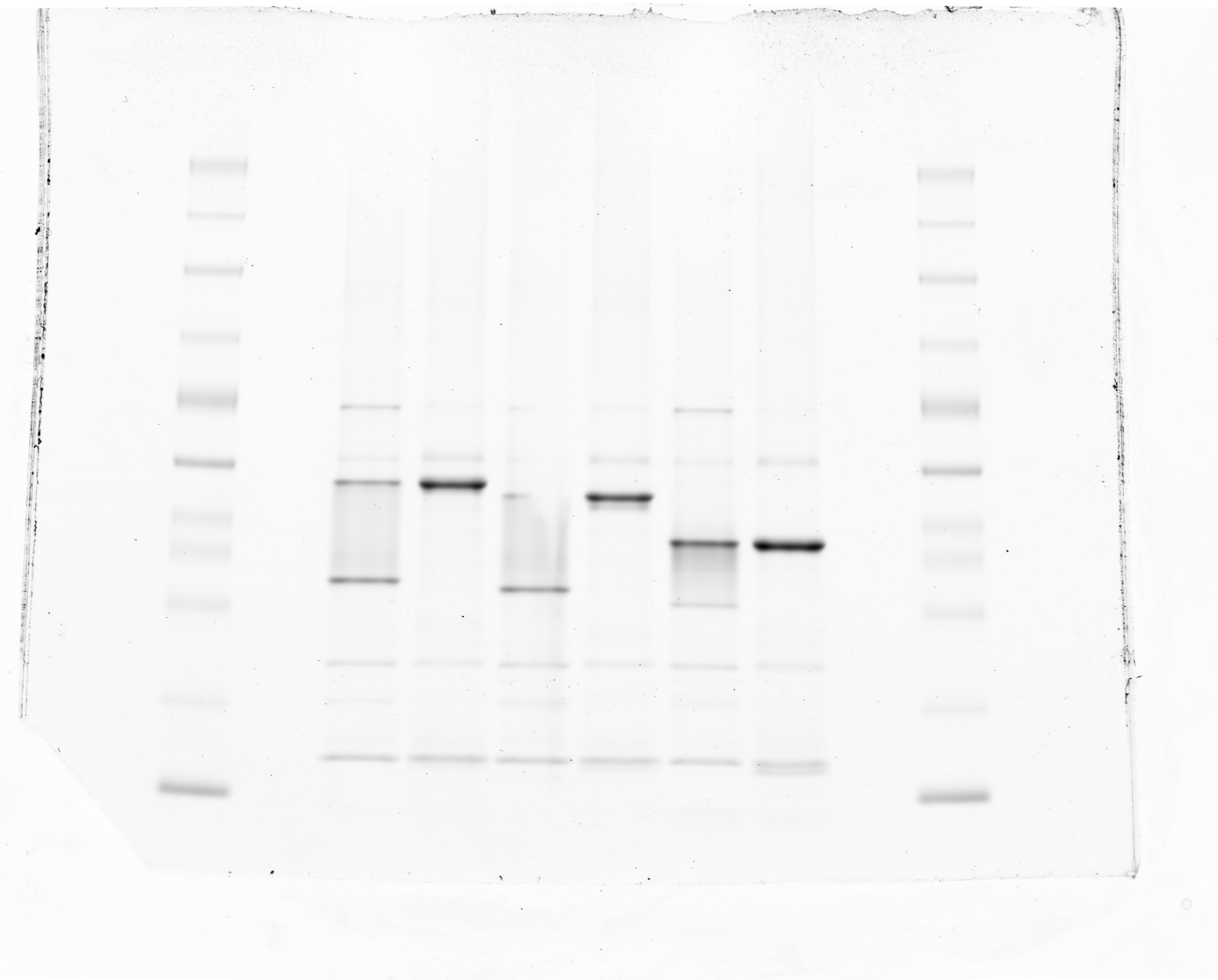

Fig. S8 top

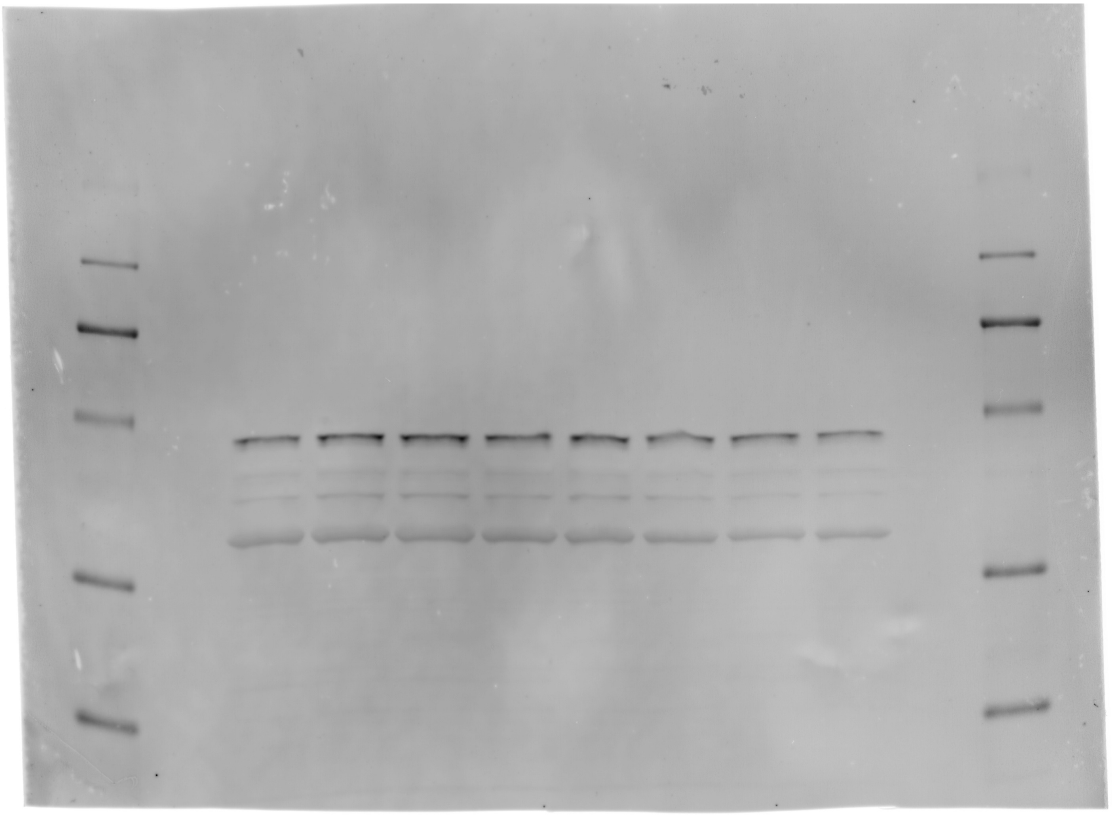

Fig. S8 bottom

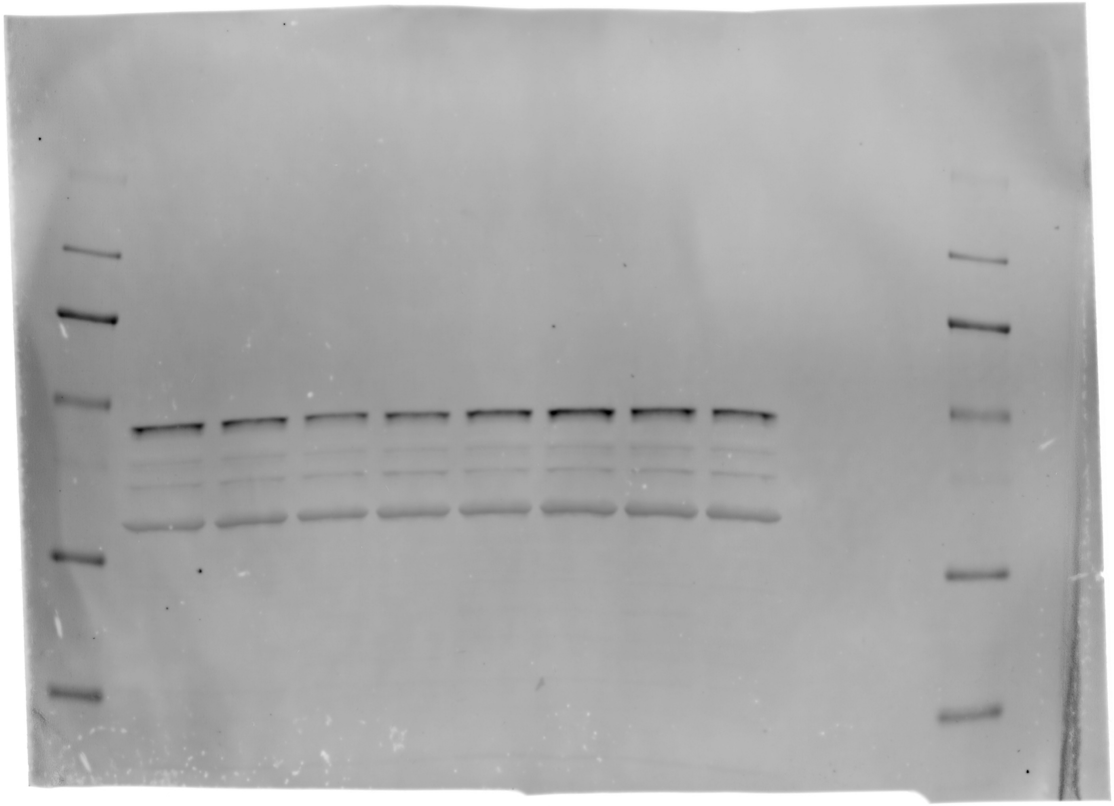

Fig. S11

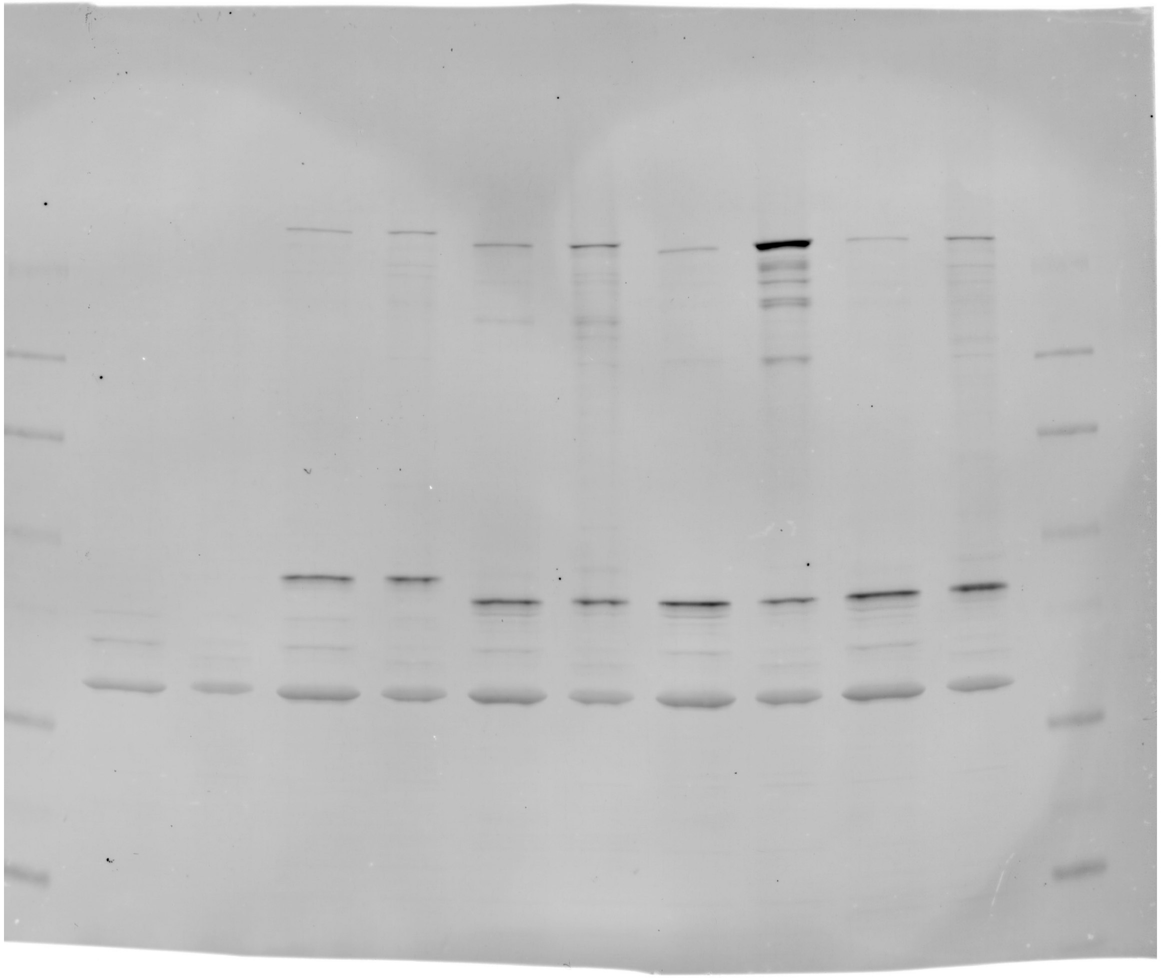

Fig. S12a

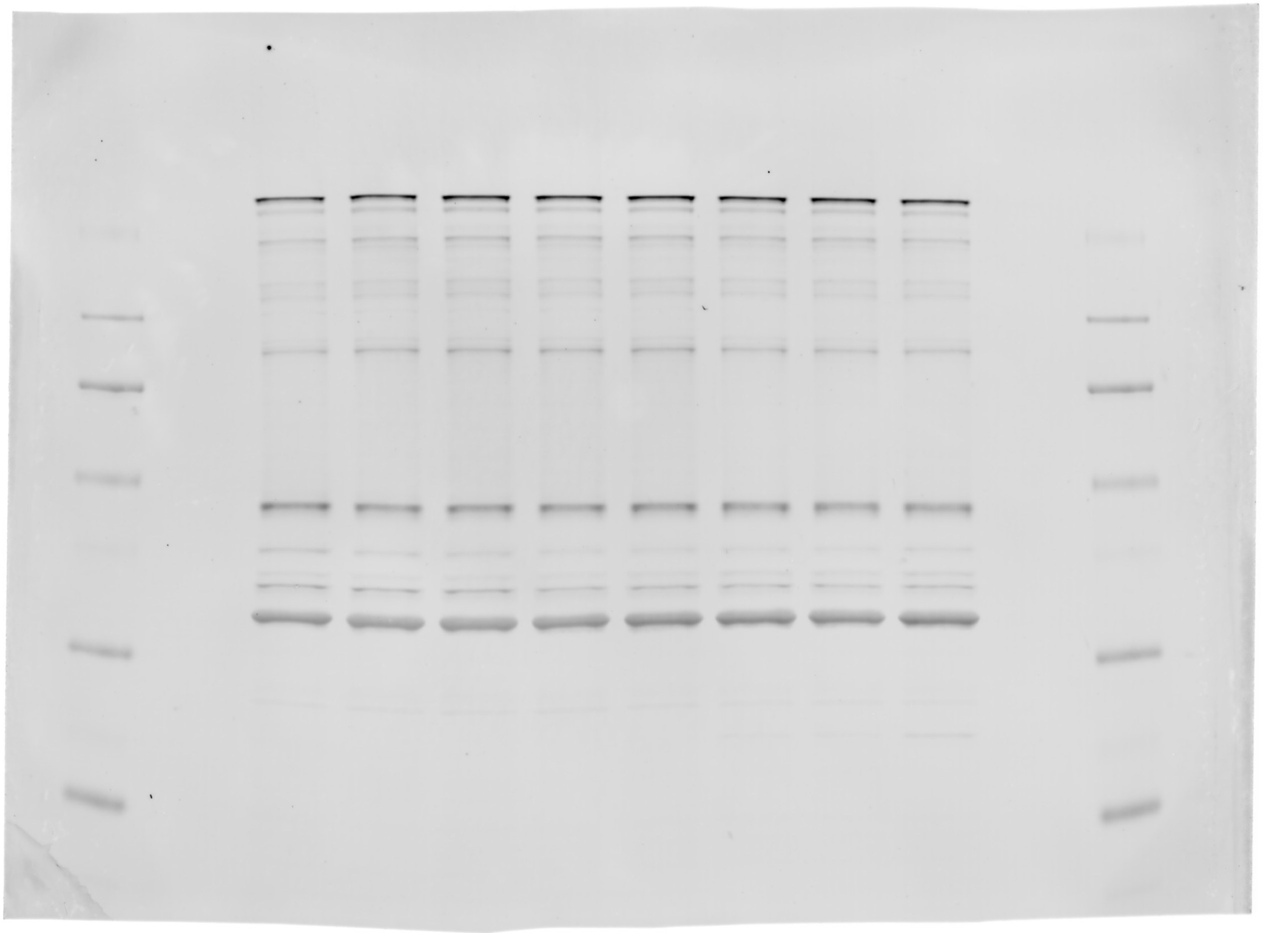

Fig. S12b

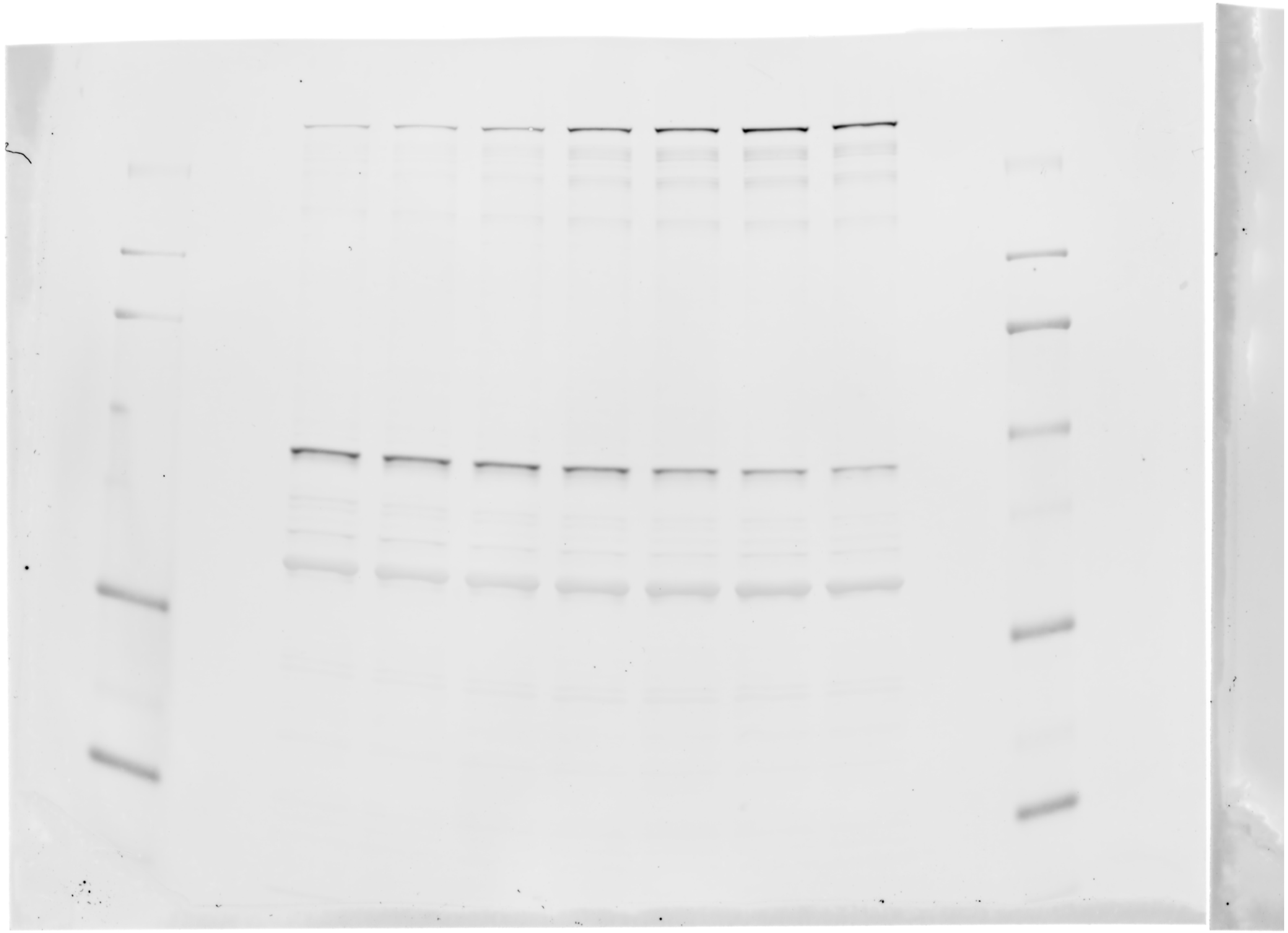

Fig.3c

	N136, Q383 C	Q135, Q383 C	L145, V388 C	V146, V388 C	N136, Q383 C	Q135, Q383 C	L145, V388 C	V146, V388 C
	17.7082114	43.3791404	24.0050048	85.0799233	51.8745447	80.5839804	51.8445752	94.3178893
	20.170769	47.3310936	23.0723766	82.533142	49.5636104	76.3336487	58.2005209	93.2086102
	31.3146566	52.0804625	21.5740255	88.1315715	34.6512469	90.8604822	74.2497062	97.1354681
MEDIAN	20.170769	47.3310936	23.0723766	85.0799233	49.5636104	80.5839804	51.8445752	94.3178893

Fig.4d

	-Ox				+Ox			
	L359 C	M249 C	F427 C	I532 C	L359 C	M249 C	F427 C	I532 C
	7.41472077	5.67792306	2.75730084	27.5002125	19.4050005	67.2738325	12.0685199	64.739636
	10.5128803	6.27707347	4.31033947	40.1818145	19.7029719	60.7362596	9.69360128	70.2883254
	14.378973	10.0878103	6.83021666	40.5854955	20.8982571	66.7360385	12.22519	66.6271213
MEDIAN	10.5128803	6.27707347	4.31033947	40.1818145	19.7029719	66.7360385	12.0685199	66.6271213

Fig.4e
I532C

Time (min)	%intra-molecular crosslinking			AVERAGE	STD ERROR
0	51.9667177	55.7450196	43.0973255	50.2696876	3.74837631
2	60.4198972	66.6872125	54.6241642	60.5770913	3.483188963
5	62.5484684	77.8048347	59.0180338	66.4571123	5.764665208
15	62.3946444	73.2003298	59.3234702	64.9728148	4.208207459
30	60.9850736	62.4748617	60.3783934	61.2794429	0.622838876
60	60.4479912	64.0990384	58.0104221	60.8524839	1.769229858
90	62.9796614	70.4178669	57.8578741	63.7518008	3.646253978
120	59.3231794	66.9762896	65.0891001	63.7961897	2.30190044

Fig.4f
M249C

Time (min)	%intra-molecular crosslinking			AVERAGE	STD ERROR
0	7.35471568	15.1449781	13.3619841	11.95389263	2.356486674
2	15.1429117	17.6842415	18.3958408	17.07433133	0.987316184
5	28.2515776	19.6945196	25.6867158	24.544271	2.535395794
15	54.4435588	49.473801	57.9683732	53.961911	2.463968863
30	72.0323379	71.4013776	81.9089166	75.1142107	3.402232058
60	87.0698879	89.8838369	85.8991269	87.61761723	1.182438725
90	88.1583605	90.4634762	91.096672	89.90616957	0.892816075

Fig. S6b

PlpD (312-728)
Replicate #1

	None	2mins	10mins	30mins
Uncut	19626.802	13624.347	6156.79	1963.285
Cut	0	4634.012	14326.669	16954.154
% Uncut	100	74.6197783	30.0573746	10.3781754
% Cut	0	25.3802217	69.9426254	89.6218246

Replicate #2

	None	2mins	10mins	30mins
Uncut	20571.924	11090.397	5734.961	1673.871
Cut	0	7277.719	13238.548	16768.447
% Uncut	100	60.3785222	30.2261485	9.07625061
%Cut	0	39.6214778	69.7738515	90.9237494

Replicate #3

	None	2mins	10mins	30mins
Uncut	19749.924	12096.104	5422.547	1549.627
Cut	0	7049.012	11245.255	15658.861
% Uncut	100	63.1811476	32.5330658	9.00501543
% Cut	0	36.8188524	67.4669342	90.9949846

AVERAGE				
% Uncut	100	66.059816	30.938863	9.48648048
% Cut	0	33.940184	69.061137	90.5135195
STDEV	0	7.5444279	1.38319676	0.77305141

PlpD (332-728)
Replicate #1

	None	2mins	10mins	30mins
Uncut	19536.045	12677.518	10578.397	2883.113
Cut	0	5150.134	7953.426	13531.861
% Uncut	100	71.1115407	57.0823335	17.5639206
% Cut	0	28.8884593	42.9176665	82.4360794

Replicate #2

	None	2mins	10mins	30mins
Uncut	19562.531	13406.225	8248.669	2482.184
Cut	0	7048.134	13506.669	16213.619
% Uncut	100	65.5421419	37.9156095	13.276691
%Cut	0	34.4578581	62.0843905	86.723309

Replicate #3

	None	2mins	10mins	30mins
Uncut	19441.459	14172.933	9364.619	2539.87
Cut	0	4945.77	10295.669	14247.326
% Uncut	100	74.1312473	47.6321557	15.1298049
% Cut	0	25.8687527	52.3678443	84.8701951

AVERAGE				
% Uncut	100	70.2616433	47.5433662	15.3234722
% Cut	0	29.7383567	52.4566338	84.6765278
STDEV	0	4.35716961	9.58367046	2.15016618

PlpD (406-728)
Replicate #1

	None	2mins	10mins	30mins
Uncut	19178.874	15912.832	11117.276	8070.083
Cut	0	3245.456	8529.941	11967.719
% Uncut	100	83.0597807	56.5844822	40.2742926
% Cut	0	16.9402193	43.4155178	59.7257074

Replicate #2

	None	2mins	10mins	30mins
Uncut	20307.873	17077.539	12049.276	5448.891
Cut	0	3552.87	8321.355	13214.77
% Uncut	100	82.7784801	59.1502345	29.1951884
%Cut	0	17.2215199	40.8497655	70.8048116

Replicate #3

	None	2mins	10mins	30mins
Uncut	21528.309	19003.439	10593.346	6505.477
Cut	0	4292.87	9066.477	16827.497
% Uncut	100	81.5727461	53.8832216	27.8810451
% Cut	0	18.4272539	46.1167784	72.1189549

AVERAGE				
% Uncut	100	82.4703356	56.5393128	32.4501754
% Cut	0	17.5296644	43.4606872	67.5498246
STDEV	0	0.7899574	2.63379693	6.80766852

Fig. S6d (Trp fluor)

LPB

Wavelength (nm)	0M Guanidine, 0.75uM protein (corrected)			4M Guanidine, 0.75uM protein (corrected)		
313	9.866666667	8.366666667	8.366666667	-6.733333333	-1.733333333	-5.033333333
314	12.2	9.5	9.6	-3	-0.1	-2.4
315	14.2	11.6	11.4	-1.266666667	3.433333333	0.133333333
316	17.1	13.9	13.5	-1.033333333	3.666666667	1.766666667
317	18.13333333	14.33333333	14.83333333	2	6	3.4
318	19.23333333	16.53333333	16.33333333	2.7	8.3	5
319	21.26666667	18.76666667	18.86666667	4.4	7.2	6
320	22.16666667	20.86666667	19.66666667	5.2	9.2	7.5
321	23.7	22.1	22.3	7.033333333	12.33333333	9.533333333
322	24.03333333	23.13333333	22.83333333	8.3	11.7	9.9
323	24.03333333	23.73333333	23.43333333	8.9	13.5	11.4
324	26.06666667	24.96666667	24.46666667	10.4	14.3	12.2
325	27.06666667	26.36666667	25.66666667	12.1	16.3	13.6
326	27	26.6	26.4	12.5	15.9	14.9
327	27.36666667	27.16666667	26.16666667	13.56666667	17.26666667	15.86666667
328	28.66666667	27.86666667	26.96666667	14.06666667	18.46666667	16.36666667
329	28.46666667	28.46666667	27.46666667	15.13333333	19.23333333	17.73333333
330	29.3	28.9	28.1	16.13333333	20.33333333	17.63333333
331	29	28.9	28.5	18.03333333	20.73333333	19.13333333
332	29.6	28.9	27.7	17.36666667	20.06666667	19.26666667
333	29.36666667	29.36666667	27.26666667	17.93333333	21.43333333	19.83333333
334	28.76666667	28.36666667	27.96666667	18.63333333	22.43333333	19.53333333
335	29.16666667	27.96666667	27.66666667	19.5	22.1	20.4
336	29.33333333	28.53333333	28.83333333	19.56666667	21.76666667	21.06666667
337	29.3	28.1	27.5	20.16666667	22.16666667	20.76666667
338	27.7	28.1	26.6	20.1	22.6	21
339	28.03333333	27.53333333	26.93333333	19.6	22.9	20.5
340	27.7	26.7	26.3	20.46666667	22.86666667	21.96666667
341	27.8	26.3	26.3	20.3	22.6	21.4
342	26.1	24.9	25.1	21	23	21.7
343	26.26666667	25.06666667	25.26666667	20.4	22.7	21.5
344	25.03333333	24.83333333	24.93333333	20.83333333	22.93333333	21.23333333
345	25.33333333	23.83333333	24.03333333	20.9	23.6	21.7
346	24.83333333	23.33333333	23.53333333	20.16666667	22.16666667	21.66666667
347	23.66666667	22.86666667	23.16666667	19.86666667	22.86666667	21.06666667
348	23.66666667	22.26666667	22.86666667	20.23333333	22.33333333	21.33333333
349	23.1	22	22.1	18.93333333	22.03333333	20.93333333
350	22	21.4	22	19.46666667	21.36666667	20.56666667
351	21.4	21.2	20.7	19.56666667	21.66666667	20.66666667
352	20.53333333	20.33333333	20.03333333	18.93333333	21.13333333	20.53333333
353	20.43333333	20.03333333	19.93333333	18.7	20.7	20.2
354	19.6	19.6	19.3	18.06666667	20.76666667	20.16666667
355	19.4	19.1	18.9	18.3	20.8	19.7
356	19.1	18.4	18.8	18.43333333	20.63333333	19.63333333
357	18.6	18.3	18.3	18.03333333	20.33333333	19.53333333
358	18.4	17.8	17.8	17.73333333	19.93333333	19.03333333
359	17.53333333	17.33333333	17.13333333	17.4	19.8	18.9
360	16.8	16.7	16.6	17.23333333	19.33333333	18.63333333
361	16.76666667	16.36666667	16.56666667	17.06666667	19.66666667	18.46666667
362	15.93333333	15.53333333	15.93333333	16.63333333	18.73333333	18.13333333
363	15.8	15.5	15.5	16.06666667	18.16666667	17.46666667
364	15.1	15	15	15.83333333	17.93333333	17.33333333
365	14.1	14.1	14.4	15.2	17.1	16.5
366	13.7	13.6	13.4	14.8	16.8	16.4
367	13.1	13.1	13.2	14.69	16.49	15.59
368	12.64333333	12.54333333	12.34333333	14	15.8	15.3
369	11.88666667	11.48666667	11.38666667	13.61	15.41	14.81
370	11.55666667	11.30666667	11.36666667	13.14	14.74	14.24
371	11.00666667	10.68666667	10.74666667	12.16666667	13.83666667	13.53666667
372	10.30666667	10.16666667	10.39666667	11.96666667	13.54666667	12.87666667
373	9.743333333	9.603333333	9.773333333	11.48333333	13.16333333	12.73333333
374	9.266666667	9.236666667	9.546666667	11.11666667	12.61666667	12.10666667
375	8.93	8.85	8.97	10.62333333	12.31333333	11.77333333
376	8.68	8.53	8.86	10.4	11.82	11.41
377	8.116666667	8.016666667	8.246666667	10.13666667	11.66666667	11.05666667
378	7.896666667	7.886666667	7.846666667	10.04333333	11.11333333	10.66333333
379	7.413333333	7.363333333	7.433333333	9.573333333	10.44333333	10.17333333
380	7.353333333	7.093333333	7.023333333	9.243333333	10.54333333	10.20333333
381	7.066666667	6.646666667	6.866666667	8.796666667	10.20666667	9.606666667
382	6.57	6.35	6.48	8.573333333	9.713333333	9.483333333
383	6.326666667	6.246666667	6.136666667	8.18	9.29	9.2
384	6.176666667	5.756666667	5.886666667	7.993333333	9.013333333	8.523333333
385	5.85	5.66	5.72	7.986666667	8.726666667	8.236666667
386	5.526666667	5.456666667	5.676666667	7.443333333	8.163333333	8.083333333
387	5.18	5.16	5.05	7.106666667	8.026666667	7.636666667
388	5.04	4.81	5.12	7.073333333	7.743333333	7.373333333
389	4.803333333	4.563333333	4.693333333	6.6	7.31	7.19
390	4.523333333	4.313333333	4.313333333	6.48	7.23	6.89
391	4.31	4.23	4.35	6.19	6.86	6.74
392	4.023333333	4.013333333	4.173333333	5.793333333	6.383333333	6.313333333
393	4.08	3.85	3.99	5.676666667	6.226666667	5.976666667
394	3.603333333	3.623333333	3.703333333	5.386666667	6.126666667	5.796666667
395	3.576666667	3.596666667	3.616666667	5.08	5.84	5.66
396	3.326666667	3.356666667	3.356666667	4.9	5.61	5.43
397	3.243333333	3.143333333	3.263333333	4.58	5.28	5.08
398	3.18	3.04	3.09	4.663333333	5.123333333	5.133333333
399	3.07	2.92	2.98	4.333333333	4.743333333	4.713333333
400	2.886666667	2.846666667	2.766666667	4.24	4.66	4.53

PB

Wavelength (nm)	0M Guanidine, 0.75uM protein (corrected)			4M Guanidine, 0.75uM protein (corrected)		
313	8.766666667	9.966666667	10.46666667	-3.833333333	-5.733333333	-7.533333333
314	11.2	12.5	11.6	-2.8	-2.2	-4.3
315	13.3	14.8	14.4	-0.566666667	0.433333333	-3.966666667
316	15.6	17.1	17.3	0.666666667	-1.033333333	-3.633333333
317	15.73333333	17.53333333	17.53333333	3.3	1.7	-0.7
318	17.63333333	19.43333333	19.13333333	3.4	1.7	-0.6
319	18.26666667	19.86666667	19.96666667	4.2	3.3	0.5
320	20.46666667	20.96666667	20.66666667	5.5	4.4	1.6
321	22.4	22.9	21.6	5.933333333	5.633333333	3.233333333
322	23.23333333	24.83333333	23.73333333	7	6.9	5
323	23.93333333	23.83333333	23.03333333	9.7	7.2	4.8
324	24.26666667	26.46666667	24.46666667	9.4	8.4	5.6
325	25.36666667	25.66666667	24.66666667	10.6	9.6	8.2
326	26.7	26.2	26.3	11.3	10.7	7.8
327	26.66666667	27.16666667	25.86666667	12.36666667	10.66666667	9.166666667
328	27.16666667	28.16666667	26.16666667	12.96666667	11.86666667	9.166666667
329	27.96666667	28.16666667	26.56666667	13.13333333	12.93333333	9.833333333
330	28.2	27.4	26.8	14.33333333	13.63333333	11.13333333
331	28.6	28.3	27.7	15.63333333	14.43333333	12.83333333
332	27.9	28	27.6	15.66666667	14.86666667	12.56666667
333	28.66666667	28.36666667	27.56666667	16.63333333	14.73333333	13.73333333
334	28.36666667	27.86666667	26.76666667	16.83333333	16.03333333	14.63333333
335	28.66666667	27.26666667	27.16666667	17.6	15.4	13.4
336	27.83333333	27.53333333	26.53333333	17.46666667	15.56666667	14.36666667
337	27.7	28	27.3	17.76666667	16.36666667	15.26666667
338	27.4	26.9	25.9	18.2	16.4	15.9
339	28.33333333	27.63333333	26.33333333	17.9	17.1	15.4
340	27.2	27	26	18.66666667	17.56666667	16.16666667
341	26.8	26.1	25.4	18.6	16.9	15.7
342	25.6	25.1	24.5	18.4	17.2	16
343	25.56666667	25.06666667	24.26666667	18.3	17.4	15.9
344	24.83333333	24.53333333	24.13333333	18.33333333	16.83333333	16.13333333
345	24.83333333	24.03333333	24.03333333	18.2	17.4	16.5
346	24.43333333	23.83333333	23.53333333	17.66666667	16.56666667	15.26666667
347	23.66666667	22.86666667	22.26666667	18.36666667	17.16666667	16.06666667
348	23.46666667	22.26666667	21.76666667	18.03333333	16.73333333	15.43333333
349	22.8	22	21.5	17.33333333	16.33333333	15.23333333
350	22.1	21.3	20.9	17.46666667	16.16666667	15.56666667
351	21.6	20.5	20.6	17.76666667	16.26666667	14.86666667
352	20.33333333	20.13333333	19.83333333	16.93333333	15.93333333	15.13333333
353	19.53333333	19.53333333	19.23333333	17	15.6	14.7
354	19.8	18.5	18.9	16.76666667	15.66666667	14.36666667
355	19.3	18.5	18.7	16.2	15.5	14.2
356	18.8	18.2	18	16.43333333	15.03333333	15.03333333
357	18.4	17.7	17.3	16.13333333	15.23333333	14.23333333
358	18.1	17.4	17.1	15.83333333	14.63333333	14.33333333
359	17.43333333	16.63333333	16.63333333	15.5	14.5	13.9
360	16.4	16.4	16.2	15.53333333	14.43333333	13.93333333
361	16.36666667	16.06666667	15.86666667	14.66666667	14.16666667	13.66666667
362	15.63333333	15.33333333	14.83333333	14.53333333	14.23333333	13.13333333
363	15.6	14.6	14.7	14.16666667	13.46666667	12.26666667
364	15.1	14.5	14.2	14.03333333	13.33333333	12.83333333
365	14.1	13.8	13.5	13.5	12.6	12.1
366	13.7	13	12.7	12.9	12.4	11.6
367	12.9	12.6	12.4	12.79	11.89	11.29
368	12.14333333	12.14333333	11.94333333	12.6	11.5	11.1
369	11.48666667	11.38666667	11.08666667	12.21	11.11	10.81
370	11.04666667	11.03666667	10.87666667	11.62	11.03	10.34
371	10.71666667	10.41666667	10.25666667	10.82666667	10.35666667	9.846666667
372	10.00666667	9.606666667	9.726666667	10.59666667	9.556666667	9.656666667
373	9.613333333	9.373333333	9.443333333	10.32333333	9.773333333	9.593333333
374	9.376666667	9.026666667	8.996666667	10.27666667	9.186666667	9.086666667
375	9.01	8.86	8.54	9.743333333	8.813333333	8.633333333
376	8.48	8.31	8.23	9.29	8.62	8.57
377	8.226666667	7.866666667	7.716666667	8.966666667	8.606666667	8.286666667
378	7.816666667	7.556666667	7.426666667	8.863333333	8.473333333	7.733333333
379	7.443333333	7.033333333	7.163333333	8.423333333	7.813333333	7.593333333
380	7.093333333	6.933333333	6.723333333	8.413333333	7.693333333	7.533333333
381	6.856666667	6.646666667	6.616666667	7.956666667	7.266666667	7.146666667
382	6.44	6.33	6.09	7.773333333	6.993333333	6.923333333
383	6.286666667	6.306666667	6.016666667	7.3	6.85	6.75
384	6.126666667	5.786666667	5.866666667	7.033333333	6.323333333	6.363333333
385	5.91	5.66	5.6	6.946666667	6.456666667	5.876666667
386	5.556666667	5.566666667	5.236666667	6.803333333	6.053333333	5.783333333
387	5.23	5.2	4.86	6.306666667	5.886666667	5.696666667
388	5.06	5.06	4.98	5.973333333	5.623333333	5.323333333
389	4.843333333	4.543333333	4.513333333	5.92	5.38	5.31
390	4.643333333	4.483333333	4.333333333	5.44	5.21	5.02
391	4.41	4.31	4.26	5.54	5.19	4.97
392	4.333333333	4.163333333	3.953333333	5.073333333	4.743333333	4.613333333
393	4.1	3.94	3.66	4.926666667	4.476666667	4.246666667
394	3.753333333	3.453333333	3.453333333	4.886666667	4.476666667	4.246666667
395	3.686666667	3.496666667	3.426666667	4.64	4.3	4.08
396	3.566666667	3.446666667	3.216666667	4.52	4.24	3.93
397	3.223333333	3.183333333	3.013333333	4.25	3.91	3.74
398	3.04	2.87	2.92	4.163333333	3.723333333	3.633333333
399	3.02	2.92	2.9	3.843333333	3.473333333	3.443333333
400	2.986666667	2.756666667	2.676666667	3.75	3.56	3.3

B

Wavelength (nm)	0M Guanidine, 0.75uM protein (corrected)			4M Guanidine, 0.75uM protein (corrected)		
313	14.6	9.6	21.1	-12.33333333	-8.933333333	-10.63333333
314	15.9	12.3	22.6	-8.7	-6.8	-6.9
315	17.6	12.3	23.2	-6.166666667	-4.366666667	-4.266666667
316	18.13333333	15.23333333	24.83333333	-4.833333333	-2.933333333	-3.733333333
317	20.4	16.3	26.9	-2.1	-0.6	-1.1
318	21.76666667	18.16666667	27.56666667	-1.6	0.7	-0.1
319	23.46666667	20.66666667	29.06666667	-0.5	1.9	1.2
320	23.83333333	20.93333333	30.83333333	2	3.8	2.8
321	24.36666667	22.26666667	31.26666667	4.033333333	5.833333333	4.633333333
322	25.13333333	23.33333333	31.83333333	4.8	6.5	5.4
323	26.16666667	25.16666667	30.96666667	6.3	8.7	6.6
324	25.93333333	25.63333333	32.53333333	7.5	9.1	7.8
325	28.3	27.5	33.4	8.4	9.9	9.2
326	27.76666667	26.36666667	33.86666667	10.4	11	10.6
327	29.93333333	28.93333333	34.73333333	10.16666667	11.66666667	11.26666667
328	29.43333333	28.03333333	34.43333333	11.96666667	12.96666667	11.76666667
329	30.23333333	28.73333333	34.23333333	12.53333333	13.63333333	12.33333333
330	29.63333333	28.93333333	35.03333333	13.23333333	14.73333333	13.93333333
331	30.73333333	28.93333333	35.03333333	14.73333333	15.93333333	14.23333333
332	30.16666667	29.16666667	34.66666667	14.66666667	16.86666667	15.36666667
333	30.9	29.2	35.1	15.73333333	17.33333333	15.73333333
334	30.1	29.5	34.6	16.13333333	17.63333333	16.83333333
335	30.4	29.7	34.1	16.9	18.4	16.6
336	30.13333333	29.53333333	34.33333333	17.56666667	18.86666667	16.86666667
337	29.8	28.5	33.4	17.86666667	19.06666667	17.66666667
338	29.73333333	28.13333333	33.23333333	18.5	19.6	18.1
339	29.2	27.7	32.6	18.5	19.6	18.1
340	28.46666667	27.46666667	31.96666667	18.96666667	20.16666667	18.56666667
341	27.8	28.3	31.9	18.2	19.4	18.9
342	27.93333333	26.83333333	30.83333333	19.1	19.6	18.8
343	27.86666667	26.36666667	30.36666667	19.1	19.9	18.9
344	26.86666667	26.56666667	30.16666667	19.43333333	20.63333333	19.33333333
345	26.26666667	25.96666667	29.76666667	19.2	20.1	19.7
346	26.06666667	24.96666667	29.66666667	19.26666667	19.56666667	18.26666667
347	24.93333333	24.33333333	28.23333333	18.76666667	19.56666667	19.16666667
348	24.53333333	24.13333333	27.53333333	18.93333333	20.23333333	19.33333333
349	23.3	22.9	26.7	17.83333333	19.13333333	18.33333333
350	23.53333333	22.63333333	26.03333333	18.06666667	18.76666667	18.66666667
351	22.16666667	22.16666667	24.96666667	18.66666667	18.66666667	18.16666667
352	21.13333333	21.03333333	24.33333333	18.23333333	18.43333333	18.13333333
353	21.03333333	20.43333333	23.93333333	17.2	18.7	17.8
354	20.96666667	20.46666667	23.86666667	18.06666667	18.06666667	17.66666667
355	19.76666667	19.16666667	23.06666667	17.2	17.5	17.3
356	19.7	19	22.2	17.53333333	17.83333333	17.33333333
357	19.26666667	18.96666667	21.86666667	17.23333333	17.83333333	17.43333333
358	18.3	18.1	21	17.03333333	17.83333333	17.13333333
359	17.76666667	17.86666667	20.36666667	16.5	16.6	16.7
360	17.53333333	17.33333333	19.63333333	16.33333333	16.83333333	16.03333333
361	17.16666667	16.76666667	19.46666667	16.26666667	16.26666667	16.26666667
362	16.16666667	15.96666667	18.06666667	16.03333333	16.23333333	16.13333333
363	15.63333333	15.13333333	17.53333333	15.36666667	15.56666667	15.66666667
364	15.1	14.7	16.9	14.63333333	14.93333333	15.03333333
365	14.5	14.2	16.3	14.4	14.7	14.5
366	13.56666667	13.66666667	15.16666667	13.8	14.4	14
367	13.39666667	12.99666667	14.99666667	13.29	13.99	13.59
368	13.00333333	12.60333333	14.30333333	13.2	13.8	13.2
369	12.05333333	11.95333333	13.55333333	12.71	13.01	12.61
370	11.19666667	11.34666667	12.74666667	12.44	12.94	12.54
371	10.78333333	10.96333333	12.49333333	11.58666667	12.07666667	11.80666667
372	10.26333333	10.18333333	11.86333333	11.30666667	11.76666667	11.31666667
373	9.836666667	9.866666667	11.18666667	10.84333333	11.44333333	11.05333333
374	9.39	9.34	10.88	10.57666667	11.04666667	10.38666667
375	8.923333333	8.943333333	10.59333333	10.05333333	10.59333333	10.12333333
376	8.826666667	8.426666667	9.786666667	9.71	10.39	10.11
377	8.396666667	8.376666667	9.506666667	9.636666667	9.996666667	9.846666667
378	7.856666667	7.776666667	8.996666667	9.273333333	9.663333333	9.623333333
379	7.47	7.44	8.61	8.903333333	9.093333333	9.093333333
380	6.97	7.24	8.26	8.663333333	9.103333333	9.093333333
381	7.106666667	6.886666667	7.936666667	8.346666667	8.636666667	8.566666667
382	6.516666667	6.276666667	7.426666667	8.073333333	8.343333333	8.183333333
383	6.29	6.07	7.2	7.72	8.17	7.85
384	5.873333333	5.573333333	6.653333333	7.293333333	7.733333333	7.353333333
385	5.476666667	5.566666667	6.436666667	7.046666667	7.626666667	7.286666667
386	5.743333333	5.343333333	6.483333333	6.903333333	7.243333333	7.053333333
387	5.153333333	5.163333333	6.123333333	6.646666667	6.836666667	6.816666667
388	5.24	4.78	5.59	6.373333333	6.493333333	6.493333333
389	4.743333333	4.553333333	5.343333333	6.16	6.4	6.33
390	4.526666667	4.176666667	5.056666667	5.97	6.32	5.93
391	4.193333333	4.223333333	4.963333333	5.81	6.15	5.83
392	3.886666667	3.756666667	4.476666667	5.483333333	5.643333333	5.673333333
393	3.946666667	3.746666667	4.456666667	5.166666667	5.266666667	5.116666667
394	3.656666667	3.516666667	4.046666667	5.006666667	5.346666667	5.096666667
395	3.573333333	3.503333333	3.963333333	4.75	5	4.91
396	3.36	3.3	3.88	4.67	4.88	4.71
397	3.223333333	2.883333333	3.653333333	4.33	4.65	4.52
398	3.053333333	2.853333333	3.483333333	4.173333333	4.443333333	4.253333333
399	2.853333333	2.793333333	3.273333333	3.993333333	4.123333333	4.023333333
400	2.656666667	2.576666667	3.066666667	3.84	4.09	3.89

Fig. S6e (CD spectra)

Wavelength (nm)	Barrel	POTRA Barrel	Linker POTRA Barrel
260	346.8402	169.2032	135.0152
259.9	264.5626	200.7682	303.1854
259.8	358.701	31.67565	271.4718
259.7	322.0648	73.99282	175.6975
259.6	345.368	88.02345	63.6829
259.5	171.0553	56.32613	8.432806
259.4	157.0109	193.4781	24.33035
259.3	81.01349	61.94715	-114.215
259.2	55.57838	-4.62489	14.63842
259.1	158.8855	-101.595	222.0535
259	282.0141	-218.598	257.4605
258.9	485.3449	-30.2949	184.9624
258.8	449.1961	7.023524	-114.56
258.7	88.2425	-7.65669	-187.828
258.6	90.82614	44.47625	-152.378
258.5	-36.6089	-173.569	36.80866
258.4	-0.44879	10.53256	7.394295
258.3	123.9984	53.89308	48.80203
258.2	170.4677	15.24643	50.28014
258.1	306.1543	14.92885	67.56016
258	255.3974	-242.283	222.873
257.9	236.3193	-247.423	-67.4606
257.8	329.3222	-4.08239	63.13289
257.7	456.4337	201.6483	-19.4908
257.6	302.73	56.08285	206.1141
257.5	-7.41614	-27.283	1.572215
257.4	-312.577	-76.1828	-21.0377
257.3	-501.223	-191.622	-22.324
257.2	-325.068	42.03224	31.04266
257.1	-105.07	190.2648	142.6138
257	42.36699	-9.44031	-34.7716
256.9	-148.637	-171.188	98.49792
256.8	-209.872	-319.182	57.90961
256.7	-263.201	-426.218	175.4117
256.6	-212.592	-321.145	241.0068
256.5	176.0687	-304.093	13.1422
256.4	222.602	-263.922	172.9564
256.3	225.8919	-127.149	240.9959
256.2	282.4006	-45.1041	454.4418
256.1	101.7505	-2.35509	404.2342
256	112.9812	14.89982	79.84469
255.9	79.9244	118.4927	-83.5029
255.8	130.5255	5.19461	-289.147
255.7	-4.99877	-117.87	-20.0227
255.6	126.1938	-236.666	22.72566
255.5	93.98461	-475.142	-3.07207
255.4	80.21316	-199.014	23.69181
255.3	72.21976	-198.885	-1.11086
255.2	-22.2656	-132.661	40.51934
255.1	184.2741	-45.848	21.92238
255	246.9584	14.04705	-192.809
254.9	370.9509	-161.084	-206.04
254.8	409.0346	45.9296	-121.884
254.7	443.4232	43.86111	23.15265
254.6	494.0719	227.42	215.6559
254.5	324.0761	-4.3237	55.89607
254.4	209.978	-221.396	-32.3943
254.3	346.3601	-337.792	-72.6802
254.2	549.8656	-286.849	57.26927
254.1	761.367	-180.402	10.34152
254	422.0623	-321.089	-27.6377
253.9	3.939217	-72.3507	-188.36
253.8	-201.956	-42.8142	148.8774
253.7	-461.01	398.1934	59.0242
253.6	-353.211	536.101	-29.4577
253.5	-188.124	503.3324	-31.9436
253.4	108.1297	391.1799	-256.404
253.3	488.7249	90.95378	-211.556
253.2	422.0722	120.6301	-431.958
253.1	233.8957	243.0618	-508.306
253	50.17502	192.4329	-289.638
252.9	95.4405	212.023	-29.939
252.8	153.1769	-35.1576	180.4176
252.7	114.1838	-109.847	227.8882
252.6	149.4542	-281.84	326.973
252.5	75.70417	-403.349	291.0852
252.4	108.5209	-316.576	294.4289
252.3	63.0777	-237.259	372.9602
252.2	87.42519	-250.099	534.447
252.1	130.253	-282.074	655.6143
252	128.8003	-331.58	696.8323
251.9	160.0093	-60.9056	539.272
251.8	270.8992	-18.6719	497.6315
251.7	331.9243	-48.8853	485.4609
251.6	422.5178	-113.303	479.5528
251.5	383.5847	-74.1506	416.0698
251.4	91.88028	-185.729	277.8485
251.3	177.9175	-136.653	118.2907
251.2	70.63388	-356.375	59.88528
251.1	78.63565	-316.776	73.76398
251	238.804	-117.663	-79.7886
250.9	223.5099	-3.91182	-290.782
250.8	-49.3821	184.5257	-335.593
250.7	-66.6404	-51.6268	-459.056
250.6	6.999776	-79.7227	-435.654
250.5	257.0492	-9.84672	-306.165
250.4	434.4229	146.1604	-284.337
250.3	350.2301	98.40196	-229.77
250.2	452.5121	-115.864	-289.944
250.1	521.7004	-46.0385	-215.882
250	275.0941	-24.8845	-31.5475
249.9	197.6428	74.43749	-119.527
249.8	-61.4911	-92.253	-64.7793
249.7	-61.4709	-271.157	-138.532
249.6	210.17	-409.398	95.43292
249.5	402.8112	-310.666	127.335
249.4	543.2065	-192.934	77.97757
249.3	407.2276	7.98878	-219.884
249.2	245.7866	42.31887	-179.933
249.1	152.9258	-131.163	-86.6002
249	265.0082	-28.2936	160.7658
248.9	348.6201	-99.6975	144.4703
248.8	518.9654	-124.165	206.067
248.7	457.7458	-49.4441	-44.3983
248.6	513.1137	-5.14561	-98.3838

248.5	277.8808	103.6329	-8.95929
248.4	169.435	269.9683	94.51381
248.3	-235.393	238.1884	-82.1785
248.2	-225.381	-1.1213	-162.056
248.1	-315.11	-300.151	-383.135
248	-184.239	-341.65	-501.774
247.9	-267.559	-167.254	-424.671
247.8	-362.408	169.3176	-306.471
247.7	-308.675	36.07029	-319.975
247.6	-107.596	-52.0822	-212.52
247.5	-115.575	-259.148	-157.866
247.4	-1.65992	-249.79	-85.8912
247.3	-299.722	-370.5	-107.522
247.2	-169.35	-386.882	-23.9686
247.1	-91.5457	-239.411	-180.628
247	-20.2419	-71.6377	-144.682
246.9	12.38741	23.07548	-143.511
246.8	-87.3235	-115.646	-164.654
246.7	-224.993	-298.195	-148.801
246.6	-178.249	-300.465	-158.978
246.5	-85.1195	-574.953	-139.015
246.4	52.71459	-663.803	-121.727
246.3	57.7445	-739.687	-125.656
246.2	-30.5207	-625.915	-141.299
246.1	-279.284	-481.498	-454.041
246	-586.568	-360.362	-534.78
245.9	-655.927	-212.032	-528.299
245.8	-651.611	-397.205	-351.98
245.7	-875.655	-592.922	-72.8014
245.6	-575.759	-543.672	-272.113
245.5	-262.886	-470.038	-326.827
245.4	16.23364	-64.6559	-435.919
245.3	176.241	17.17324	-335.28
245.2	-80.6563	-36.5564	-364.481
245.1	-272.28	-78.0407	-401.262
245	-447.921	-270.331	-362.115
244.9	-559.255	-152.56	-35.5911
244.8	-258.642	-289.255	-99.3374
244.7	-157.196	-380.002	-175.12
244.6	222.9415	-408.222	-519.022
244.5	60.87416	-396.316	-485.798
244.4	107.8284	-568.762	-326.577
244.3	91.04992	-455.154	-202.085
244.2	183.4762	-598.526	-239.029
244.1	112.7318	-514.875	-317.755
244	-184.795	-555.403	-280.872
243.9	-374.37	-496.236	-343.777
243.8	-448.746	-390.415	-320.771
243.7	-363.152	-398.727	-372.484
243.6	-300.002	-476.579	-280.262
243.5	-334.056	-600.197	-275.377
243.4	-287.27	-984.609	-359.333
243.3	-448.379	-1020.13	-276.331
243.2	-363.578	-814.128	-225.219
243.1	-475.874	-636.384	-496.357
243	-657.022	-815.878	-727.439
242.9	-555.589	-697.117	-784.982
242.8	-672.913	-874.855	-821.941
242.7	-602.304	-595.031	-498.705
242.6	-746.301	-600.255	-604.705
242.5	-874.2	-643.361	-490.223
242.4	-755.316	-704.167	-517.828
242.3	-652.697	-771.82	-778.887
242.2	-707.038	-1084.16	-956.384
242.1	-740.959	-1055.14	-1114.21
242	-872.226	-988.461	-956.561
241.9	-967.731	-1016.34	-1070.23
241.8	-973.745	-959.122	-986.128
241.7	-970.205	-1119.57	-967.981
241.6	-883.016	-875.698	-1128.72
241.5	-567.088	-867.342	-873.304
241.4	-858.536	-786.237	-976.009
241.3	-733.17	-1164.33	-1068.74
241.2	-828.222	-1297.07	-1302.46
241.1	-919.448	-1267.83	-1226.86
241	-778.762	-871.412	-1018.44
240.9	-846.471	-819.668	-997.451
240.8	-906.418	-802.826	-1003.39
240.7	-888.988	-859.17	-1213.53
240.6	-1050.63	-862.83	-1316.56
240.5	-910.31	-1150.95	-1271.72
240.4	-1139.69	-1297.01	-1302.75
240.3	-1224.33	-1384.85	-1376.35
240.2	-1419.92	-1245.88	-1593.95
240.1	-1503.01	-957.563	-1442.11
240	-1440.43	-830.967	-1161.59
239.9	-1002.45	-1048.1	-1095.24
239.8	-762.034	-1305.78	-971.947
239.7	-931.602	-1498.11	-1038.75
239.6	-1198.31	-1424.1	-1055.29
239.5	-1510.05	-1291.7	-1239.71
239.4	-1454.44	-1352	-1480.6
239.3	-1667.3	-1310.65	-1788.04
239.2	-1800.43	-1631.24	-1903.61
239.1	-1633.48	-1711.35	-2006.61
239	-1522.12	-1714.13	-1680.66
238.9	-1415.36	-1685.56	-1584.09
238.8	-1475.25	-1584.46	-1472.5
238.7	-1677.57	-1523.78	-1532.81
238.6	-1618.12	-1447.07	-1791.91
238.5	-1663.33	-1700.71	-1795.39
238.4	-1706.23	-1618.4	-2069.54
238.3	-1687.91	-1606.79	-2076.59
238.2	-1699.23	-1521.05	-2116.92
238.1	-1599.92	-1416.46	-1810.2
238	-1467.73	-1588.18	-2039.06
237.9	-1741.93	-1773.17	-2164.69
237.8	-1803.84	-1811.33	-2077.99
237.7	-2097.66	-1802.92	-2181.79
237.6	-2299.82	-1927.01	-2157.71
237.5	-2338.89	-2108.32	-2388.32
237.4	-2244.9	-2125.24	-2614.09
237.3	-2269.54	-1883.79	-2501.83
237.2	-2302.8	-1721.47	-2253.53
237.1	-2241.37	-1804.61	-2188.78
237	-2022.28	-2198.28	-2370.69
236.9	-1969.88	-2579.19	-2708.57
236.8	-2008.47	-2602.93	-2759.25
236.7	-2186.59	-2391.68	-2610.45

236.6	-2227.29	-2302.42	-2454.55
236.5	-2392.14	-2436.21	-2750.05
236.4	-2447.53	-2784.92	-2905.95
236.3	-2771.32	-2771.64	-3057.47
236.2	-2626.45	-2546.82	-3112.74
236.1	-2593.53	-2471.23	-3073.48
236	-2442.26	-2614.64	-3070.32
235.9	-2174.07	-2752.55	-3059.27
235.8	-2333.26	-2647.86	-3185.42
235.7	-2530.76	-2498.85	-3093.68
235.6	-2819.85	-2622.46	-3035.04
235.5	-3042.76	-2875.99	-2822.63
235.4	-3183.77	-2992.72	-2853.06
235.3	-3125.11	-3043.17	-2968.28
235.2	-3219.15	-3005.65	-3187.85
235.1	-3246.47	-3234.51	-3193.12
235	-3187.66	-3322.93	-3194.67
234.9	-3315.33	-3461.6	-3181.44
234.8	-3215.06	-3640.83	-3398.79
234.7	-3385.74	-3760.28	-3557.29
234.6	-3534.92	-3821.89	-3693.47
234.5	-3564.9	-3697.3	-3789.63
234.4	-3706.06	-3570.99	-3927.07
234.3	-3878.78	-3516.05	-4216.82
234.2	-3693.43	-3665.67	-4264.02
234.1	-3573.86	-3829.03	-4372.96
234	-3386.18	-3979.04	-4338.55
233.9	-3398.14	-3989.22	-4076.19
233.8	-3646.91	-3876.46	-4206.64
233.7	-3850.3	-3855.69	-4321.91
233.6	-3928.68	-3721.22	-4368.17
233.5	-4298.64	-3823.59	-4393.38
233.4	-4203.05	-3696.35	-4184.47
233.3	-4319.44	-3665.08	-4365.6
233.2	-4229.93	-3970.73	-4426.77
233.1	-4441.84	-4294.55	-4267.98
233	-4584.55	-4676.71	-4392.26
232.9	-4620.24	-4732.21	-4600.17
232.8	-4607	-4545.94	-4903.22
232.7	-4729.33	-4608.89	-5035.23
232.6	-5071.73	-4487.15	-5274.39
232.5	-5413.13	-4482.45	-5161.21
232.4	-5560.55	-4482.45	-5257.51
232.3	-5236.85	-4355.52	-5266.74
232.2	-5401.83	-4681.75	-5403.89
232.1	-5194.13	-4928.9	-5401.9
232	-5179.78	-5179.27	-5560.98
231.9	-5113.76	-5154.33	-5656.55
231.8	-5582.48	-5235.6	-5770.51
231.7	-5895.47	-5396.18	-5582.54
231.6	-6261.17	-5474.71	-5465.16
231.5	-6156.62	-5619.85	-5454.03
231.4	-6248.88	-5563.99	-5794.87
231.3	-6024	-5718.21	-6092.09
231.2	-6036.33	-5736.48	-6342.84
231.1	-6083.48	-5780.72	-6528.99
231	-6345.04	-5995.39	-6590.11
230.9	-6430.32	-6115.86	-6424.63
230.8	-6435.95	-6312.46	-6540.5
230.7	-6663.35	-6383.42	-6510.13
230.6	-6580.53	-6457.16	-6541.48
230.5	-6736.56	-6430.54	-6641.01
230.4	-6565.86	-6444.89	-6579.77
230.3	-6743.04	-6210.2	-6781.85
230.2	-6920.75	-6362.87	-6835.54
230.1	-7251.12	-6448.22	-6969.61
230	-7441.2	-6222.11	-6963.2
229.9	-7590.55	-6192.96	-7139.42
229.8	-7544.74	-6350.86	-7174.58
229.7	-7165.75	-6517.43	-7243.25
229.6	-7074.2	-6861.29	-7308.36
229.5	-7348.76	-7151.13	-7455.17
229.4	-7757.22	-7239.41	-7586.57
229.3	-8054.97	-7285.99	-7496.06
229.2	-8012.28	-7375.78	-7675.88
229.1	-8110.68	-7521.8	-7711.16
229	-7825.9	-7702.76	-7778.28
228.9	-8017.89	-7607.42	-7704.56
228.8	-7980.33	-7396.43	-7826.73
228.7	-8255.04	-7512.57	-7947.34
228.6	-8589.47	-7410.24	-8310.93
228.5	-8966.47	-7794.5	-8223.75
228.4	-9253.02	-7854.23	-8483.12
228.3	-9251.12	-7952.52	-8384.75
228.2	-9183.5	-7984.49	-8537.37
228.1	-8931.61	-8008.04	-8474.33
228	-8995.64	-8088.75	-8551.74
227.9	-8974.99	-8144.17	-8673.18
227.8	-9246.1	-8075.69	-8772.87
227.7	-9016.84	-7867.85	-8892.63
227.6	-8899.46	-7788.14	-8878.79
227.5	-8903.96	-7833	-8890.73
227.4	-9208.07	-8225.8	-8855.31
227.3	-9767.85	-8313.42	-9104.26
227.2	-10097.5	-8388.43	-9215.18
227.1	-10259.7	-8468.74	-9445.58
227	-10354.1	-8632.68	-9437.39
226.9	-10322.7	-8747.53	-9792.46
226.8	-10363.8	-8605.04	-9621.92
226.7	-10199.3	-8675.79	-9538.14
226.6	-10197.5	-8961.34	-9387.44
226.5	-10023	-9128.89	-9140.61
226.4	-10000.9	-9252.38	-9329.99
226.3	-10226.3	-9216.73	-9391.74
226.2	-10191.1	-9011.05	-9502.42
226.1	-10495.1	-9102.03	-9658.85
226	-10377.1	-9294.09	-9694.7
225.9	-10505.9	-9242.42	-9703.05
225.8	-10642.9	-9659.89	-10020.5
225.7	-10987.4	-9661.28	-10137.2
225.6	-11234.9	-9852.57	-10374.9
225.5	-11046.3	-9890.87	-10323.8
225.4	-11047.9	-9604.25	-10311.9
225.3	-11185.7	-9557.54	-10230.7
225.2	-11238.7	-9486.7	-10239.8
225.1	-11578.9	-9317.45	-10256.1
225	-11527.7	-9642.31	-10281.6
224.9	-11379.4	-9821.15	-10645.4
224.8	-11117.7	-9901.73	-10759.1

224.7	-10926.1	-9969.63	-10982.6
224.6	-11152.6	-9713.47	-10842.4
224.5	-11610	-9829.16	-10961.6
224.4	-12007.9	-9749.24	-10992.9
224.3	-12134.9	-10148.9	-11107.5
224.2	-11939.5	-10333.1	-11074.7
224.1	-11769.6	-10527.3	-10739.8
224	-11660.3	-10496.7	-10698.3
223.9	-11510.6	-10105.5	-10776.8
223.8	-11707.3	-9802.6	-10771
223.7	-11443.2	-9773.67	-10906.5
223.6	-11548.7	-9788.91	-10855.1
223.5	-11519.2	-10052.1	-11206.3
223.4	-11490.8	-10082.6	-11179.4
223.3	-11747.2	-10156.1	-11171.1
223.2	-12131.1	-10110.6	-11034.9
223.1	-12394.5	-10188.9	-10654.9
223	-12479.6	-10468.1	-10870.6
222.9	-12224.2	-10706	-11006.7
222.8	-12096.3	-10926	-11060.3
222.7	-12237.1	-11022.1	-11297.1
222.6	-12290.9	-10873.5	-11017.8
222.5	-12384.6	-10762.5	-10905.8
222.4	-12563.1	-10418.8	-11000.6
222.3	-12356.9	-10139.1	-11063.9
222.2	-12411.4	-10114.8	-11342.3
222.1	-12349.4	-9983.58	-11408.1
222	-12655	-10332.7	-11636.3
221.9	-12778.8	-10729.3	-11398.4
221.8	-12763.1	-10923.7	-11364.5
221.7	-12669.2	-11117.9	-11094
221.6	-12434.4	-11148.9	-11085.4
221.5	-12542.1	-11079.7	-11264.8
221.4	-12611.6	-11115.1	-11406.1
221.3	-12644.4	-10954.3	-11419
221.2	-12788.8	-10769	-11474.2
221.1	-12800.6	-10824.7	-11604.4
221	-12855	-10819.6	-11680.1
220.9	-12899.9	-11306	-11722.1
220.8	-13113.4	-11148.4	-11566.7
220.7	-13181.3	-11341.6	-11716.7
220.6	-13171.6	-11248.2	-11761.2
220.5	-13091.7	-11264.8	-11992.7
220.4	-12746.2	-11122.5	-11917.2
220.3	-12842.9	-11080.3	-11932.4
220.2	-13156.9	-11136.6	-11914
220.1	-13116	-11230.7	-11743.9
220	-13113.6	-11482.6	-11898.9
219.9	-13102.6	-11557.4	-11840.2
219.8	-12924.9	-11393.8	-11710.8
219.7	-13080.2	-11139	-11556.8
219.6	-13286	-11234.8	-11648.4
219.5	-13192.2	-11340.3	-11718.2
219.4	-13303.5	-11533.7	-12028.7
219.3	-13077.4	-11699.6	-12128.9
219.2	-13077.7	-11493.5	-11832
219.1	-13291.5	-11336	-11781.2
219	-13391.2	-11187	-11885
218.9	-13257.3	-11284.1	-11602.8
218.8	-13042.5	-11321.3	-11497.6
218.7	-12722.7	-11405.2	-11211.5
218.6	-12852.3	-11274.3	-11180.8
218.5	-13114.8	-11343.7	-11611.7
218.4	-13175.2	-10886.7	-11673.9
218.3	-12981.1	-10981.6	-11872.3
218.2	-12895.7	-10747.2	-11825.9
218.1	-12970.5	-11145	-11789.1
218	-12757.9	-11362.5	-11680.5
217.9	-12422.9	-11439.5	-11537.2
217.8	-12318.2	-11399.9	-11576.9
217.7	-12351.8	-10788.2	-11637.8
217.6	-12834.5	-10570.2	-12031.4
217.5	-13324.7	-10538.7	-12053.7
217.4	-13410.4	-10700.4	-11725.4
217.3	-13142.3	-10734.1	-11398.7
217.2	-12927.7	-10792.4	-11093.3
217.1	-12730.8	-10637.8	-11329.4
217	-12602.7	-10856.6	-11698.8
216.9	-12679.9	-10964.8	-11720.5
216.8	-12943.7	-11230.7	-11627.9
216.7	-13064.5	-11276.8	-11574.8
216.6	-13045.4	-11023.9	-11885.7
216.5	-12856.5	-10874.3	-12131.5
216.4	-12382.2	-10771.3	-11857
216.3	-12462	-11024.1	-11522.6
216.2	-12581.4	-11268	-11250
216.1	-12620	-11424.1	-11042.2
216	-12851	-11480.5	-11271.5
215.9	-12707.9	-11327.9	-10946.3
215.8	-12497.2	-11139.8	-10990.6
215.7	-12274.2	-10933.8	-10763.6
215.6	-12531.7	-10761.4	-10882.9
215.5	-12245.6	-10768.3	-11156.7
215.4	-12194.4	-10877.8	-11274
215.3	-11934.5	-10937.1	-11181.4
215.2	-11944.9	-11022.4	-11012
215.1	-12353.4	-10851.5	-10720.4
215	-12330.1	-10647.4	-10591.3
214.9	-12879.4	-10577.4	-10717.7
214.8	-12720.5	-10437.9	-10854.1
214.7	-12607.9	-10354.3	-10713.2
214.6	-12651.1	-10248.8	-10972
214.5	-12075	-10293	-10775.2
214.4	-11859.2	-10304.7	-10857.7
214.3	-11089	-10506.9	-10724.2
214.2	-10850.8	-10344.5	-10664.2
214.1	-11129.6	-10675.5	-10852.6
214	-11488.9	-10649.3	-10627.4
213.9	-11730.1	-10758.6	-10629.7
213.8	-11671	-10389.4	-10137.8
213.7	-11387.1	-10145.1	-10006
213.6	-11261.6	-9992.57	-10012.3
213.5	-11068.8	-9693.96	-10168.8
213.4	-10764.9	-10067.2	-10339.8
213.3	-10625.5	-10128.7	-10375.4
213.2	-10384	-10068.2	-10438.8
213.1	-10490.9	-9935.32	-10058.4
213	-10733.6	-9723.99	-9945.08
212.9	-10760.6	-10053.8	-9547.25

212.8	-10797.4	-10143.8	-9907.34
212.7	-10891.8	-10227.3	-10076.5
212.6	-10856.4	-10185.9	-10044.7
212.5	-10684.3	-10175.5	-10056.1
212.4	-10546.7	-10136.8	-10137.7
212.3	-10158.3	-9896.26	-10129.7
212.2	-9773.32	-9597.52	-10069.4
212.1	-9168.53	-9330.91	-9967.47
212	-9450.85	-9260.58	-9862.93
211.9	-9533.56	-9268.69	-9436.74
211.8	-10120.3	-9319.1	-9416.1
211.7	-10316.5	-9468.53	-9198.69
211.6	-10024.6	-9508.22	-9450.73
211.5	-9727.11	-9238.84	-9701.81
211.4	-9410.67	-9125.38	-9576.97
211.3	-9265.8	-8910.35	-9615.24
211.2	-9248.46	-8890.06	-9436.3
211.1	-9132.07	-8716.72	-9109.31
211	-8628.19	-8973.59	-9042.99
210.9	-8585.21	-8996.41	-9054.91
210.8	-8276.75	-9098.86	-9275.11
210.7	-8299.4	-9191.74	-9249.61
210.6	-8271.58	-9120.51	-9220.72
210.5	-8162.97	-9025.05	-9242.16
210.4	-8152.87	-8675.2	-9293.25
210.3	-7980.11	-8307.21	-9398.18
210.2	-8039.07	-8223.68	-9491.13
210.1	-7761.75	-8397.19	-9351.89
210	-7570.91	-8717.53	-8956.57
209.9	-7192.11	-9038.98	-8673.81
209.8	-7142.63	-8837.29	-8346.87
209.7	-7591.88	-8730.04	-8252.94
209.6	-7831.98	-8662.54	-8290.92
209.5	-8206.15	-8570.27	-7933.35
209.4	-8112.53	-8194.83	-7783.24
209.3	-7934.74	-7631.77	-7771.07
209.2	-6726.79	-7502.54	-7977.48
209.1	-6324.17	-7835.02	-8003.99
209	-6279.8	-8126.35	-8110.62
208.9	-6567.93	-8048.86	-7709.22
208.8	-6446.17	-7900.65	-7613.5
208.7	-6203.17	-8025.67	-7372.71
208.6	-5563.13	-7774.65	-7172.91
208.5	-5172.89	-7624.86	-7141.58
208.4	-5257.03	-7270.6	-7140.25
208.3	-5279.58	-7127.9	-6812.91
208.2	-5572.49	-7196.09	-6515.44
208.1	-5120.26	-6931.96	-6476.25
208	-4515.25	-6662.65	-6074.66
207.9	-4215.38	-6681.3	-6084.79
207.8	-3843.31	-6352.81	-5735.5
207.7	-3620.34	-6117.98	-5728.17
207.6	-3407.2	-5923.94	-5495.29
207.5	-3163.41	-5386.22	-5603.59
207.4	-3048.42	-5370.56	-5556.02
207.3	-2486.37	-5317.96	-5408.48
207.2	-2010.36	-5094.38	-5305.5
207.1	-1538.24	-4681.63	-4988.65
207	-1300.56	-4373.45	-4192.68
206.9	-1478.96	-3905.78	-3943.85
206.8	-1327.54	-3944.19	-3915.25
206.7	-1141.38	-3957.39	-3671.23
206.6	-528.987	-3791.81	-3904.95
206.5	-211.718	-3447.84	-3156.96
206.4	167.8902	-3183.08	-2780.65
206.3	454.7991	-2834.01	-2230.27
206.2	1150.358	-2606.73	-1632.95
206.1	1610.428	-2344.08	-1238.02
206	1824.794	-2016.58	-1108.25
205.9	2250.845	-1895.59	-1268.33
205.8	2341.926	-1760.99	-1510.08
205.7	2912.335	-2031.5	-1196.92
205.6	3260.431	-1657.4	-695.834
205.5	3671.942	-1676.97	-177.11
205.4	4091.257	-1247.68	206.932
205.3	4499.838	-662.181	508.6317
205.2	4694.565	-436.782	877.3715
205.1	4998.546	206.9482	1343.088
205	5446.934	359.9066	1582.577
204.9	5788.482	665.1858	1436.927
204.8	6587.305	973.7283	1746.458
204.7	6783.238	1218.175	1732.395
204.6	7349.767	1353.436	2569.894
204.5	7680.901	1929.898	3070.051
204.4	8048.79	2298.192	3564.696
204.3	8336.129	2682.125	4067.597
204.2	8720.486	3094.167	4335.195
204.1	9109.817	3393.897	4497.236
204	9458.602	3621.226	4031.221
203.9	9688.828	3715.494	4257.878
203.8	10068.26	4014.517	4405.835
203.7	10861.4	4499.788	5343.037
203.6	11255.46	4912.317	6061.595
203.5	11783.85	5626.038	6045.204
203.4	12455.84	6093.896	6272.323
203.3	12775.88	6331.673	6397.672
203.2	13459.49	6602.544	7209.817
203.1	13769.97	6896.989	8117.37
203	13947.26	6895.893	8567.649
202.9	14221.8	7110.609	8697.782
202.8	14602.79	7251.39	8892.119
202.7	15293.41	7776.354	9285.431
202.6	16081.9	8124.131	9890.077
202.5	16314.91	8412.366	10349.03
202.4	16467.73	8442.295	10427.73
202.3	17196.62	8734.768	10450.7
202.2	17242.43	9377.598	10596.93
202.1	17477.21	9880.572	10747.87
202	17076.21	10096.6	11297.69
201.9	17520.24	10085.5	11932.59
201.8	18203.68	10439.36	12484.38
201.7	19074.86	11190.66	12840.29
201.6	19557.73	12112.1	13016
201.5	19978.65	12353.73	13401.08
201.4	20053.17	12654.97	13664.65
201.3	20780.4	13299.43	14420.99
201.2	21121.41	13840.83	14671.38
201.1	21629.74	14547	14884.29
201	22043.05	14173.51	15182.78

200.9	22354.51	14008.79	15455.96
200.8	22958.02	13821.57	16147.2
200.7	23702.61	14208.24	16366.67
200.6	24551.23	14909.83	16713.19
200.5	24991.99	15402.82	17119.05
200.4	25311.78	16086.68	17209.19
200.3	25395.23	16449.68	17774.31
200.2	25828.26	16650.66	18067.57
200.1	26577.87	16893.36	18516.47
200	27106.17	17491.96	18687.76
199.9	27358.99	17994.84	18948.29
199.8	27776.26	18736.71	19191.39
199.7	27912.4	19060.16	19565.71
199.6	28275.27	19043.48	19844.04
199.5	29147.72	19010.75	20228.61
199.4	29744.95	19003.88	20604.68
199.3	29994.08	18994.41	21669.42
199.2	29901.76	19287.49	21542.32
199.1	29865.58	19799.96	22142.33
199	30461.33	20222.55	22385.28
198.9	30789.73	21344.83	23086.33
198.8	31429.55	21620.09	23878.98
198.7	31704.28	22239.44	23695.47
198.6	32366.01	22495.33	23837.04
198.5	32312.34	23000.57	23459.56
198.4	32671.76	23186.5	23715.07
198.3	33096.17	23805.62	24374.55
198.2	33874.59	24161.01	25350.97
198.1	34361.53	24353.86	25761.59
198	34523.51	24557.45	26152.79
197.9	34694.36	24832.39	26576.29
197.8	34623.21	24820.21	26671.96
197.7	35017.34	25002.61	26801.63
197.6	35882.66	24952.69	27090.36
197.5	35902.85	25180.87	26817.66
197.4	35802.16	25344.76	27132.37
197.3	35291.34	25458.24	27106.63
197.2	35298.72	25663.29	26839.51
197.1	36066.8	25812.81	27488.36
197	36621.69	25856.4	27929.58
196.9	37025.91	25585.85	28134.53
196.8	37519	25690.81	28267.35
196.7	37846.17	26020.54	28237.24
196.6	38004.71	25826.94	28519.41
196.5	38366.84	26772.3	28712.22
196.4	37714.72	27361.87	29071.61
196.3	37853.31	27556.2	28452.88
196.2	37934.31	27887.39	28054.39
196.1	38557.38	27828.46	28646.9
196	38678.5	28005.26	29718.99
195.9	39180.46	28838.11	30679.39
195.8	39469.47	28454.56	30813.67
195.7	40001.22	28631.36	30463.27
195.6	39378.14	28609.93	30468.61
195.5	38537.94	28663.99	30394.3
195.4	38111.55	28315.75	29453.82
195.3	38639.36	28556.11	29320.99
195.2	39371.74	28198.37	29264.17
195.1	39696.2	28437.27	29567.47
195	39778.43	28218.59	29934.87
194.9	40204.81	27964.59	29524.73
194.8	39815.6	28332.07	29898.93
194.7	39389.71	28973.51	30765.1
194.6	39489.66	29375.81	30914.2
194.5	39877.14	29360.23	30738.39
194.4	40674.27	28000.15	30774.09
194.3	40174.28	27729.83	29793.06
194.2	39651.4	27256.67	29943.12
194.1	39433.53	27894.7	29559.94
194	38993.36	27939.02	29284.33
193.9	38179.99	28349.12	29376.84
193.8	37916.83	28167.45	28729.22
193.7	37114.04	28149.43	28687.69
193.6	36778.01	27969.71	28239.43
193.5	36531.34	27845.51	28328.3
193.4	37181.25	27911.26	29226.53
193.3	36674.12	27245.71	29845.26
193.2	37181.74	26408.96	29103.9
193.1	36827.74	26698.02	28332.92
193	37445.4	26945.93	27539.11
192.9	37236.64	27466.83	27627.5
192.8	37604.43	27463.91	27751.58
192.7	37359.48	26961.27	26954.37
192.6	37577.59	27008.27	27165.15
192.5	38192.55	27142.94	26677.79
192.4	37672.87	27200.17	27720.02
192.3	38209.53	26337.85	27837.55
192.2	37209.56	25858.11	27567.52
192.1	36318.15	24410.6	26806.49
192	35068.06	24587.64	25982.32
191.9	34605.98	24677.75	25656.69
191.8	34439.81	25622.86	25583.35
191.7	33613.64	26181.27	26141.38
191.6	33645.64	25779.21	26128.51
191.5	33921.36	25660.61	26354.83
191.4	33637.02	24906.17	26155.7
191.3	33217.29	23879.45	25897.09
191.2	32236.52	23669.97	25810.64
191.1	32032.44	23459.98	25095.27
191	32200.57	23868.18	24979.92
190.9	32268.77	24079.56	24104.28
190.8	31364.8	24620.03	23197.43
190.7	30957.13	24742.53	22962.98
190.6	30327.41	25424.88	22424.26
190.5	30208.5	25003.58	23550.6
190.4	29828.16	24087.64	24059.21
190.3	29600.94	21692.51	23762.74
190.2	28566.74	20061.26	23960.67
190.1	28753.1	21005.14	23255.76
190	28336.56	21417.16	23448.17
